# Supplementary material for: Glyco-Decipher enables glycan database-independent peptide matching and in-depth characterization of site-specific N-glycosylation
Source: Nat Commun. 2022 Apr 7;13:1900. doi: 10.1038/s41467-022-29530-y (PMC8990002; doi:10.1038/s41467-022-29530-y)
Supplement: Supplementary file 1 — Supplementary Information [file 41467_2022_29530_MOESM1_ESM.pdf]

Supplementary Information for

Glyco-Decipher enables glycan database-independent peptide matching and in-depth characterization of site-specific N-glycosylation

M.-M. Dong, M.-L. Ye et al.

The Supplementary Information includes:

Supplementary Table 1 Page S3

Supplementary Table 2 Page S4

Supplementary Table 3 Page S5

Supplementary Figure 1-44 Page S6-S78

Supplementary Note 1 (Supplementary Figure 45-48) Page S79-S86

Supplementary Note 2 (Supplementary Figure 49-52) Page S87-S93

Supplementary References Page S94

Supplementary Table 1 Spectrum identification rate achieved by pGlyco 2.0 and Glyco-Decipher on the mouse tissue dataset.

Dataset	PRIDE Accession	#Raw File	#Spectrum (MS2)	#Spectrum (GlycoPeptide)	#Spectrum pGlyco 2.0	ID Rate (%)	#Spectrum This study	ID Rate (%)
Mouse Brain	PXD005411	5	233,623	208,653	17,792	8.53	45,326	21.72
Mouse Heart	PXD005413	5	318,339	265,112	5,890	2.22	20,537	7.75
Mouse Kidney	PXD005412	5	254,511	244,423	22,176	9.07	57,820	23.66
Mouse Liver	PXD005553	5	226,702	220,492	19,048	8.64	44,646	20.25
Mouse Lung	PXD005555	5	353,669	343,583	17,144	4.99	46,681	13.59
Average			1,386,844	1,282,263	82,050	**6.40**	215,010	**16.77**

The spectrum identification rate of Glyco-Decipher was compared with pGlyco 2.0, the work which firstly introduced the dataset of mouse tissues.

Supplementary Table 2 Oxonium ions considered in Glyco-Decipher.

Oxonium Ion	*m/z*	Oxonium Ion	*m/z*
HexNAc_$C_4H_8O_4$	84.0444	NeuAc_H_2O	274.0874
Hex_$C_2H_6O_3$	85.0284	NeuGc_H_2O	290.0823
Hex_CH_6O_3	97.0284	NeuAc	292.1027
Hex_4H_2O	109.027	NeuGc	308.0976
Hex_$C_5H_7O_3$	115.039	HexHex	325.1129
HexNAc_$C_2H_6O_3$	126.0550	HexNAcHex	366.1395
Hex_2H_2O	127.039	HexNeuAc	454.156
HexNAc_$C_1H_6O_3$	138.0550	HexNAcHexFuc	512.197
HexNAc_$C_2H_4O_2$	144.064	HexNAcHex(2)	528.1917
Hex_H_2O	145.067	HexNAcHexNeuAc	657.2349
Hex	163.060	HexNAcHex(3)	690.2445
HexNAc_2H_2O	168.066	HexNAcHexNeuAcFuc	803.293
HexNAc_H_2O	186.076	HexNAc(2)Hex(3)	893.3239
HexNAc	204.0867	HexNAc(2)Hex(3)Fuc	1039.3823

Supplementary Table 3 Spectrum expansion results of peptide backbone "MHLNGSNVQVLHR" in the "MouseLiver-Z-T-1" LC-MS/MS file.

File Name	Scan	RT (min)	Peptide	Glycan
MouseLiver-Z-T-1	14293	68.79	MHLNGSNVQVLHR	H(9)N(2)
MouseLiver-Z-T-1	14302	68.79	MHLNGSNVQVLHR	H(10)N(2)
MouseLiver-Z-T-1	14339	68.99	MHLNGSNVQVLHR	H(9)N(2)
MouseLiver-Z-T-1	14387	69.19	MHLNGSNVQVLHR	H(9)N(2)
MouseLiver-Z-T-1	14420	69.35	MHLNGSNVQVLHR	H(8)N(2)
MouseLiver-Z-T-1	14434	69.39	MHLNGSNVQVLHR	H(8)N(2)
MouseLiver-Z-T-1	14475	69.59	MHLNGSNVQVLHR	H(7)N(2)
MouseLiver-Z-T-1	14593	70.12	MHLNGSNVQVLHR	H(5)N(4)
MouseLiver-Z-T-1	16897	80.15	MHLNGSNVQVLHR	H(5)N(4)G(1)
MouseLiver-Z-T-1	16985	80.56	MHLNGSNVQVLHR	H(5)N(4)G(1)
MouseLiver-Z-T-1	16998	80.61	MHLNGSNVQVLHR	H(5)N(4)G(1)
MouseLiver-Z-T-1	17086	81.01	MHLNGSNVQVLHR	H(4)N(4)G(1)
MouseLiver-Z-T-1	17108	81.08	MHLNGSNVQVLHR	H(4)N(4)G(1)
MouseLiver-Z-T-1	17276	81.84	MHLNGSNVQVLHR	H(5)N(4)G(1)
MouseLiver-Z-T-1	17400	82.37	MHLNGSNVQVLHR	H(5)N(4)G(1)
MouseLiver-Z-T-1	19520	91.69	MHLNGSNVQVLHR	H(5)N(4)G(2)
MouseLiver-Z-T-1	19522	91.69	MHLNGSNVQVLHR	H(5)N(4)G(2)
MouseLiver-Z-T-1	19712	92.56	MHLNGSNVQVLHR	H(5)N(4)G(2)
MouseLiver-Z-T-1	19714	92.56	MHLNGSNVQVLHR	H(5)N(4)G(2)
MouseLiver-Z-T-1	19892	93.34	MHLNGSNVQVLHR	H(5)N(4)G(2)
MouseLiver-Z-T-1	19922	93.49	MHLNGSNVQVLHR	H(5)N(4)G(2)
MouseLiver-Z-T-1	19960	93.65	MHLNGSNVQVLHR	H(5)N(4)G(2)
MouseLiver-Z-T-1	19993	93.8	MHLNGSNVQVLHR	H(5)N(4)G(2)
MouseLiver-Z-T-1	20063	94.1	MHLNGSNVQVLHR	H(5)N(4)G(2)
MouseLiver-Z-T-1	20064	94.1	MHLNGSNVQVLHR	H(5)N(4)G(2)

Green: Glycopeptide spectra initially identified by in silico deglycosylation and others were achieved by spectrum expansion.

RT: Retention time. Monosaccharide abbreviation: H: Hex; N: HexNAc; G: NeuGc.

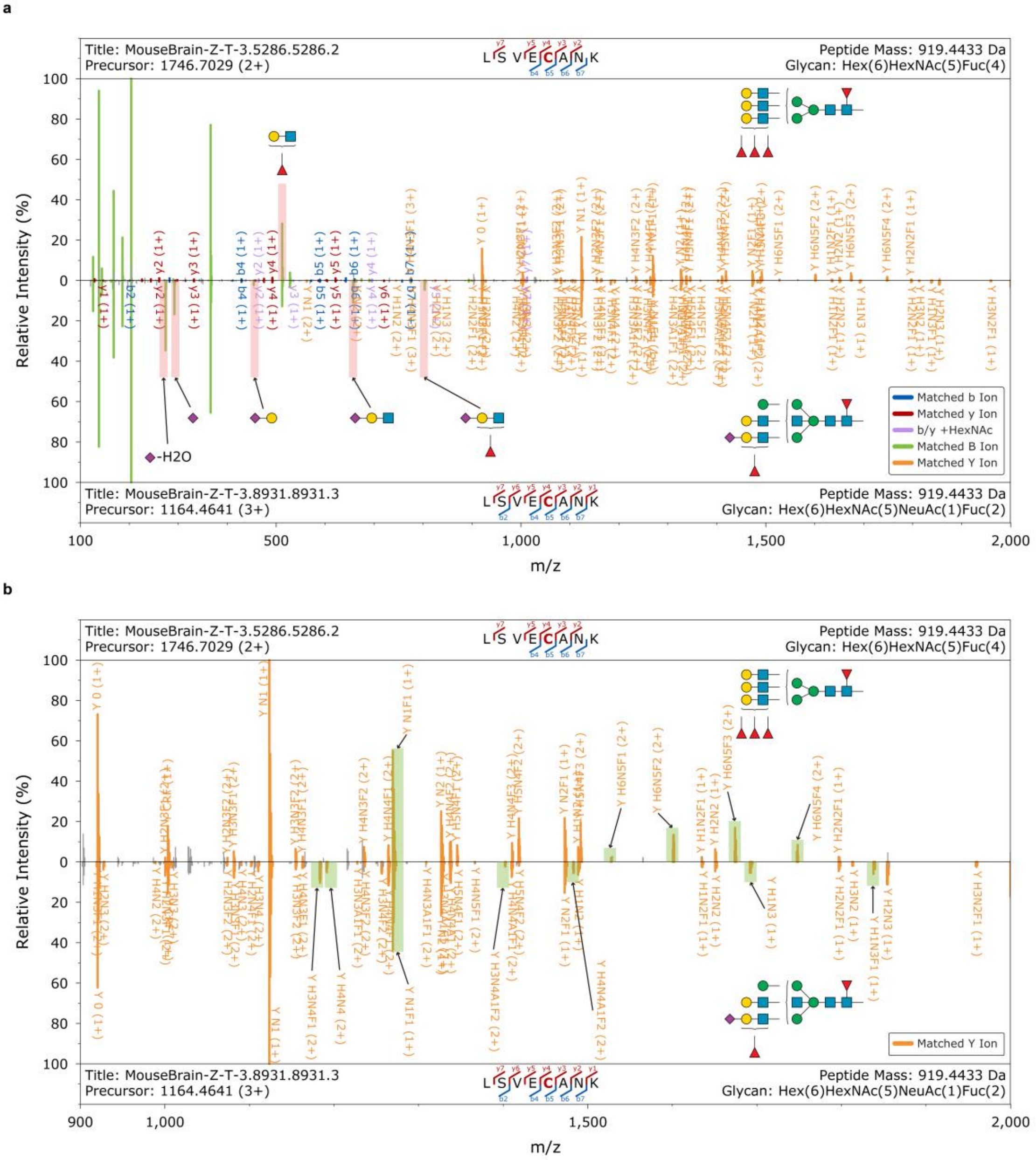

Supplementary Figure 1 Spectrum examples of glycopeptides with the same peptide backbones but different glycan compositions identified by Glyco-Decipher. (a) Spectrum of glycopeptides with the same peptide backbone “LSVECANK” but different glycans (top: Hex(6)HexNAc(5)Fuc(4), bottom: Hex(6)HexNAc(5)NeuAc(1)Fuc(2)). Possible structure illustration of the glycans are shown in the figure. The mass values of glycan parts are close (Mass(NeuAc) + 1 Da = Mass(Fuc)*2) and were differentiated by Glyco-Decipher by precursor correction and

diagnostic glycan ion matching. Diagnostic B ions are labeled in the figure.

(b) High *m/z* section (ranging from 900 to 2,000) of the glycopeptide spectra shown in (a). The selected diagnostic Y ions for glycan composition discrimination are labeled in the figure. And it should be note that the location of branch structures of N-Glycans are unable to be determined by Glyco-Decipher.

a

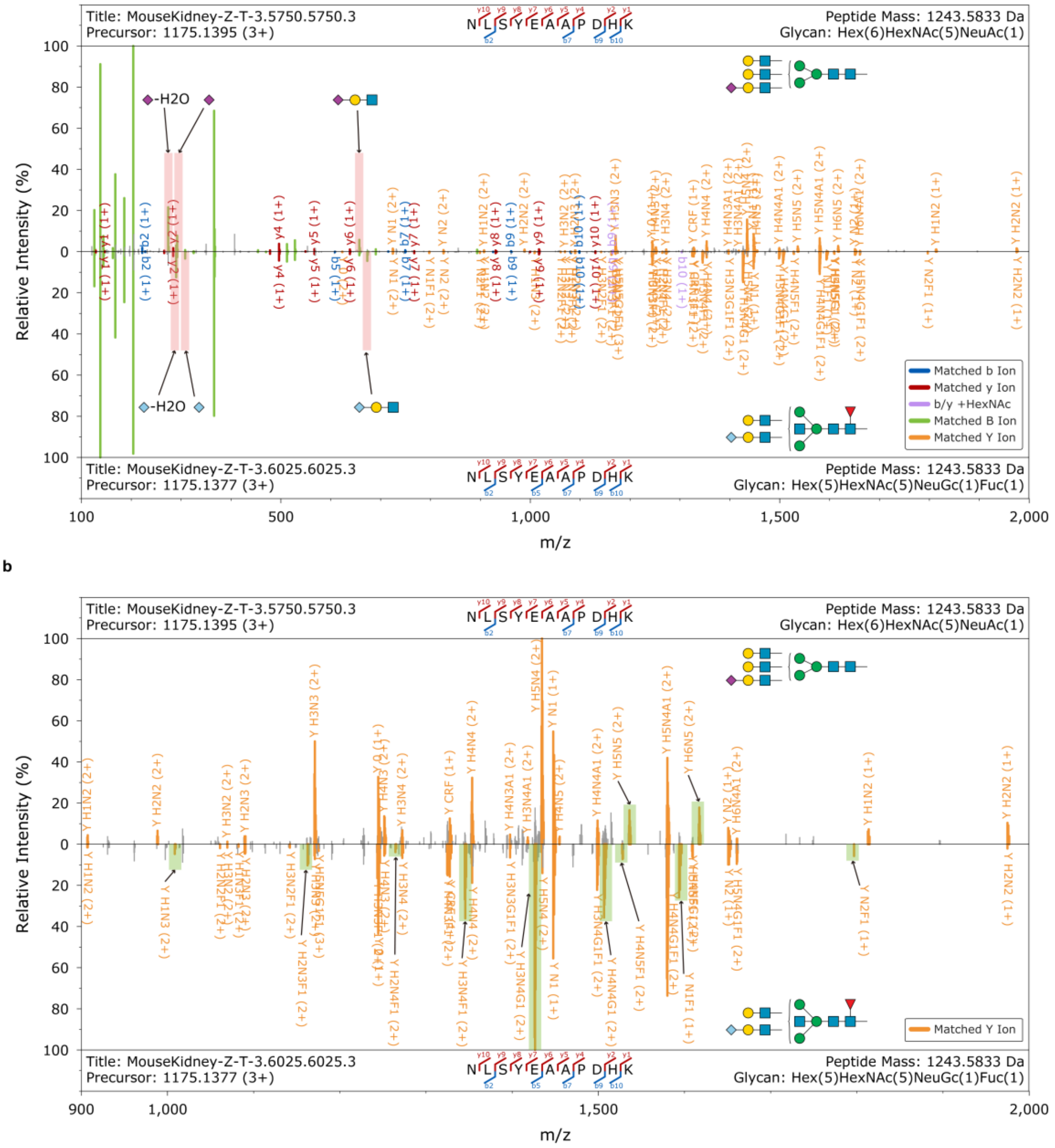

Supplementary Figure 2 Spectrum examples of glycopeptides with the same peptide backbones but different glycan compositions identified by Glyco-Decipher. (a) Spectrum of glycopeptides with the same peptide backbone “NLSYEAAPDHK” but different glycans (top: Hex(6)HexNAc(5)NeuAc(1), bottom: Hex(5)HexNAc(5)NeuGc(1)Fuc(1)). Possible structure illustration of the glycans are shown in the figure. The mass values of glycan parts are identical (Mass(NeuAc) + Mass(Hex) = Mass(NeuGc) + Mass(Fuc)) and were differentiated by Glyco-Decipher

by diagnostic glycan ion matching. Diagnostic B ions are labeled in the figure.

(b) High *m/z* section (ranging from 900 to 2,000) of the glycopeptide spectra shown in (a). The selected diagnostic Y ions for glycan composition discrimination are labeled in the figure.

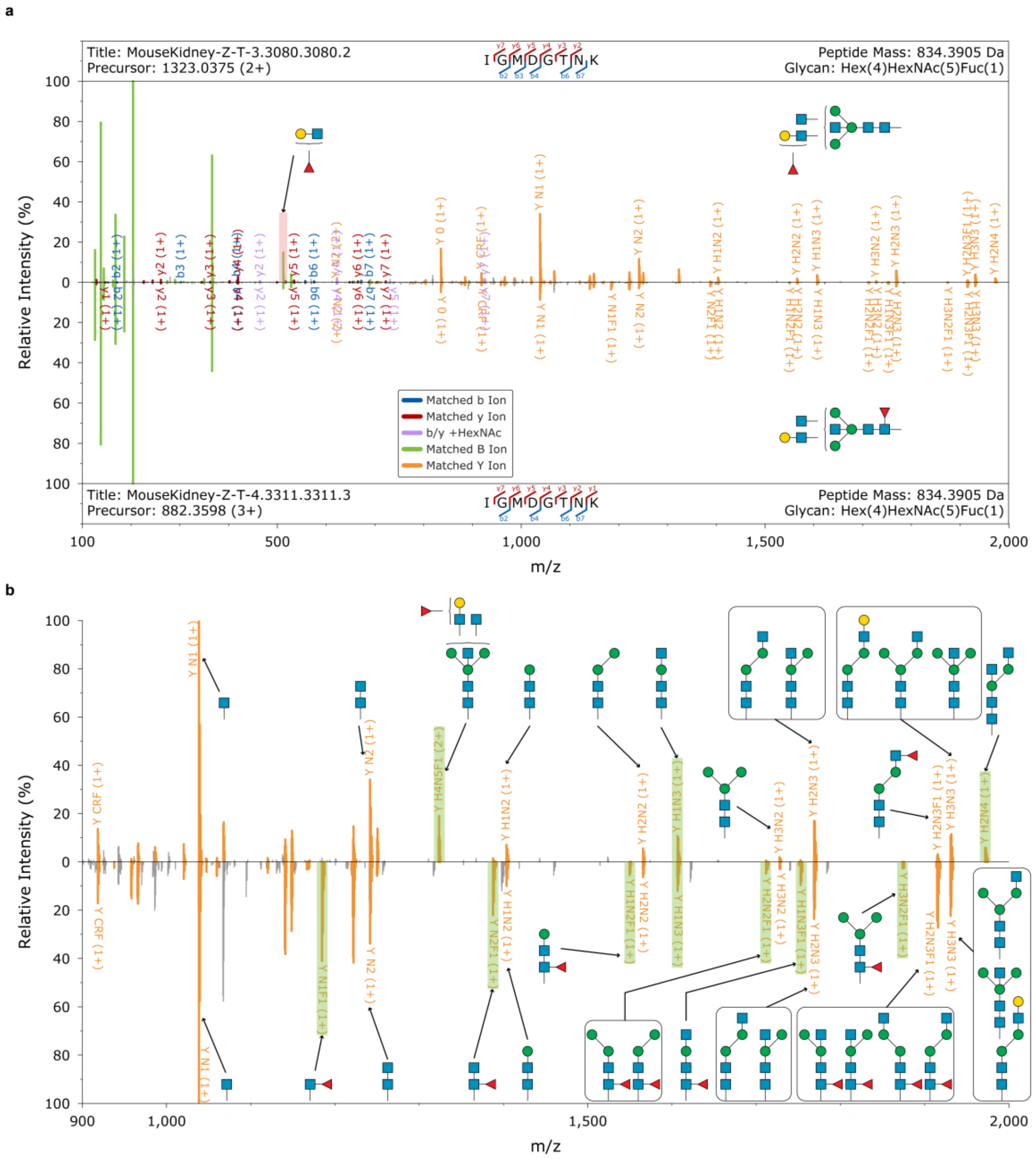

Supplementary Figure 3 Spectrum examples of distinguishing isobaric glycan structures by Glyco-Decipher.

(a) Spectra of glycopeptides with the same peptide backbone “IGMDGTNK” and identical glycan composition (Hex(4)HexNAc(5)Fuc(1)). The isobaric structures of the glycan parts were discriminated by Glyco-Decipher by matching their diagnostic fragment ions. Diagnostic B ion is labeled in the figure.

(b) High *m/z* section (ranging from 900 to 2,000) of the glycopeptide spectra shown in

(a). The selected Y ions for structure discrimination, including core fucosylation and bisected HexNAc, are labeled in the figure. And it is worth to mention that the fine structures of N-Glycans, including location of branch structures and α-2,3/α-2,6 mannose, are unable to be determined by Glyco-Decipher.

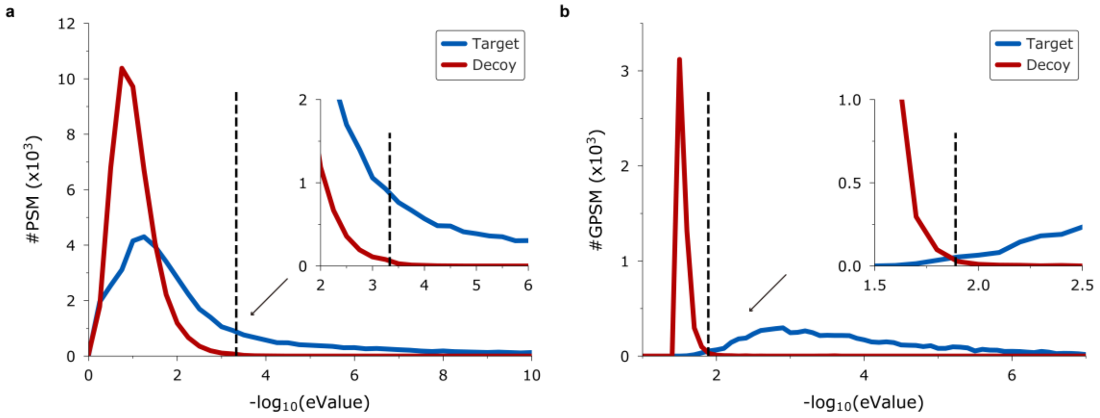

Supplementary Figure 4 Quality control in spectrum expansion and glycan annotation.

(a) e-value distributions of target (blue) and decoy (red) peptide-spectrum matches (PSMs) in spectrum expansion of file "MouseLiver-Z-T-1". FDR threshold was set to 1% at spectrum level in Glyco-Decipher for PSM identification. The e-value threshold for 1% peptide FDR is indicated by dashed line.

(b) e-value distributions of target (blue) and decoy (red) glycopeptide-spectrum matches (GPSMs) in glycan annotation during the identification of the file "MouseLiver-Z-T-1". Decoy spectra were generated by shifting *m/z* values of experimental fragment ions with 1-30 *m/z* randomly and were matched with the theoretical glycan fragment ions to assess the possibility of random matching and false discovery rate of glycan annotation. FDR threshold was set to 1% at spectrum level in Glyco-Decipher for glycan assignment. The e-value threshold for 1% glycan FDR is indicated by dashed line.

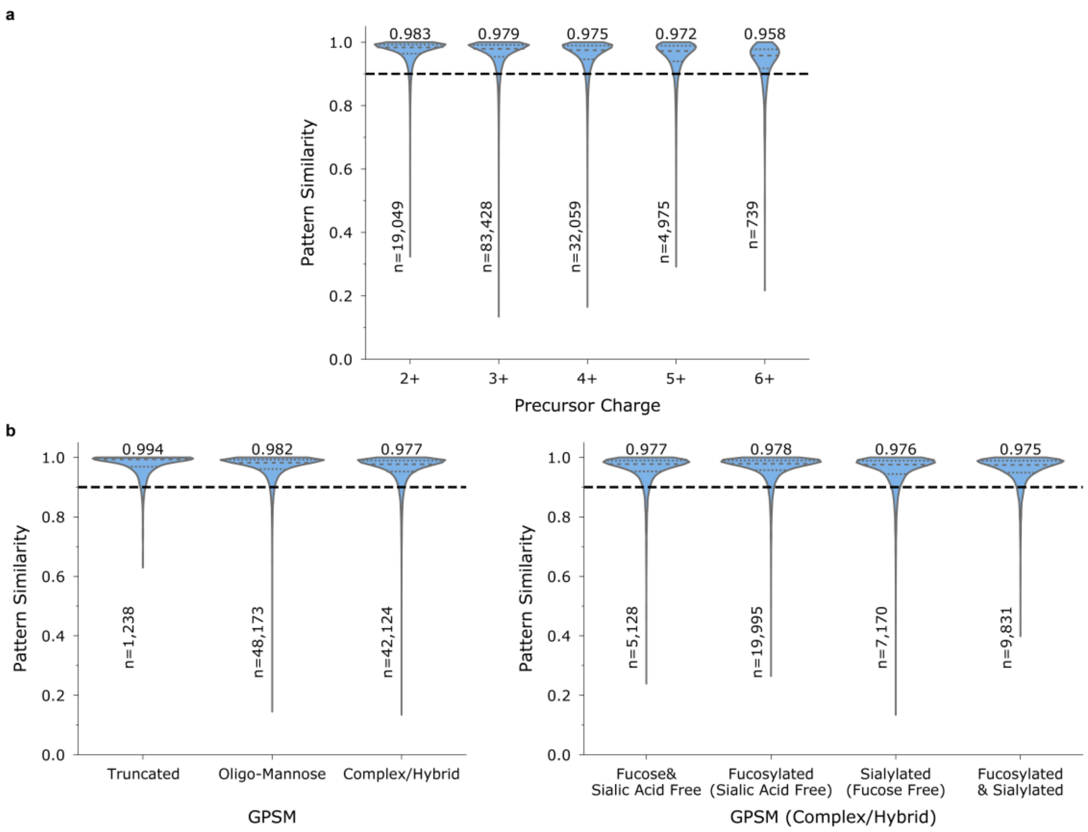

Supplementary Figure 5 Distribution of the similarities of peptide fragmentation pattern.

Cosine similarity of peptide fragmentation patterns between each PSM and the average pattern of corresponding peptide was calculated. The quartiles of the distributions are indicated by inner dashed lines. The medians and the spectrum numbers are labeled in the plot. The similarity value of 0.9 is indicated by outer dashed lines. Source data are provided as a Source Data file.

(a) Distribution of pattern similarities of 140,250 PSMs from in silico deglycosylation results of five mouse tissue datasets. These PSMs were classified by their precursor charge states.

(b) Distribution of pattern similarities of 91,535 GPSMs in which the glycan part was

matched with GlyTouCan database. The GPSMs were classified into three main categories based on the glycan composition (left): GPSMs with truncated glycans (Hex(<4)HexNAc(<3)Fuc(<2)); GPSMs with oligo-mannose glycans (Hex(>3)HexNAc(2)Fuc(<2)) and GPSMs with complex/hybrid glycans. Specifically, the GPSMs with complex/hybrid glycans were further divided into four subgroups based on the glycan compositions (right): GPSMs with fucose and sialic acid free glycans (no fucose and sialic acid in glycan composition); GPSMs with fucosylated but sialic acid free glycans; GPSMs with sialylated but fucose free glycans and GPSMs with glycans containing both fucose and sialic acid.

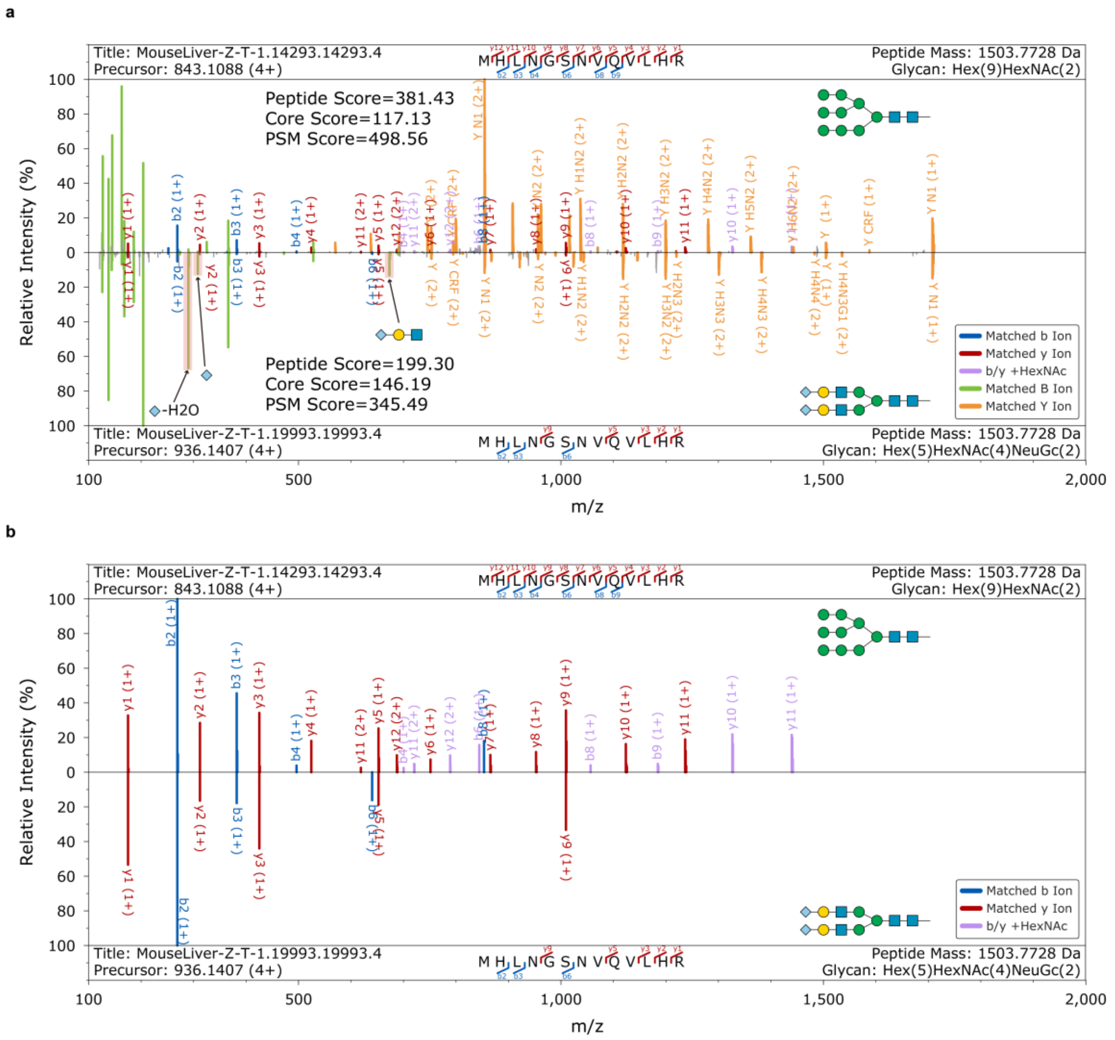

Supplementary Figure 6 Examples of GPSMs from in silico deglycosylation identification and spectrum expansion method.

(a) Intact glycopeptide spectrum matched by in silico deglycosylation (top) and spectrum expansion method (bottom).

(b) Normalized peaks of peptide part of intact glycopeptides shown in (a). Peptide fragmentation pattern was retained after spectrum expansion: b2 ion is still the most abundant ion and followed by b3, y1, y9 ions.

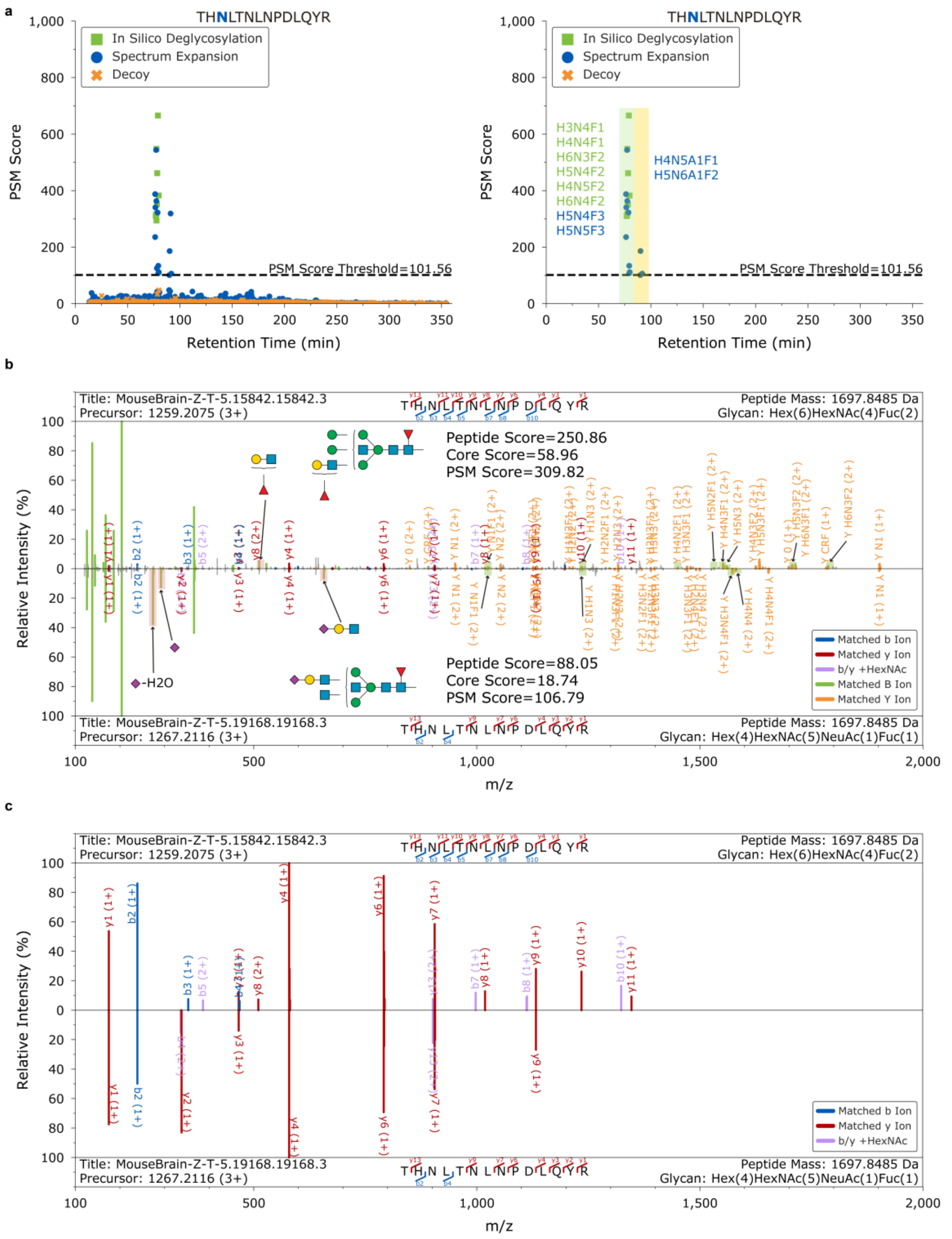

Supplementary Figure 7 Examples of GPSMs with peptide backbone "THNLTNLNPDLQYR" identified from in silico deglycosylation and spectrum expansion.

(a) Score distribution of peptide-spectrum matches in spectrum expansion. Left: score

distribution of all target-decoy PSMs obtained in spectrum expansion. Right: score distribution of PSMs after score filtering and core structure peak matching. And the PSM score threshold was derived from the e-value filtration method. Source data are provided as a Source Data file.

(b) The GPSM initially identified by in silico deglycosylation (top) and the GPSM that passed the score threshold in the spectrum expansion (bottom).

(c) Normalized peaks of peptide part of intact glycopeptides shown in (b). Peptide fragmentation pattern was retained after spectrum expansion: y4 ion is still the most abundant ion and followed by y6, y7, b2 ions.

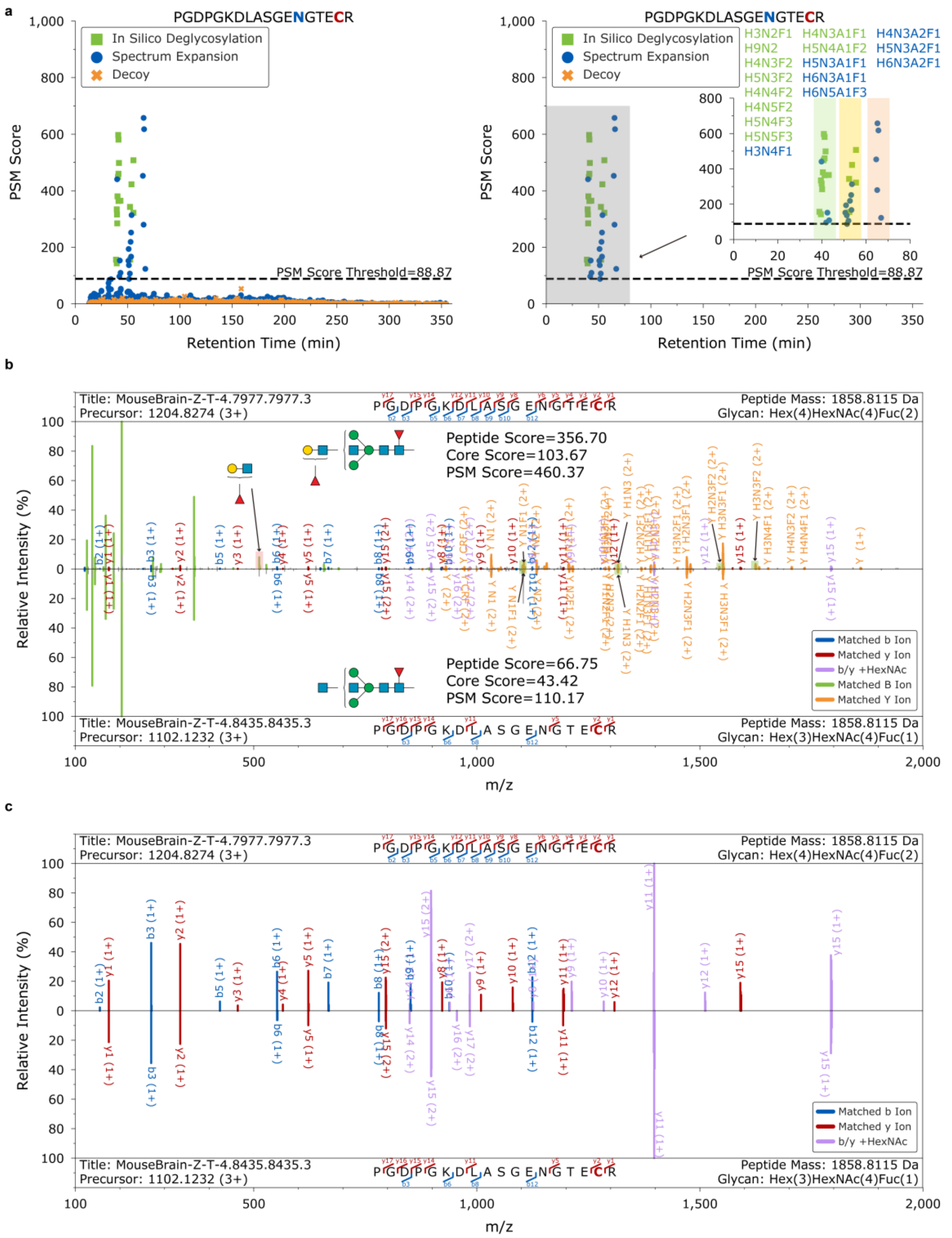

Supplementary Figure 8 Examples of GPSMs with peptide backbone "PGDPGKDLASGENGTECR" identified from in silico deglycosylation and spectrum expansion.

(a) Score distribution of peptide-spectrum matches in spectrum expansion. Left: score

distribution of all target-decoy PSMs obtained in spectrum expansion. Right: score distribution of PSMs after score filtering and core structure peak matching. And the PSM score threshold was derived from the e-value filtration method. Source data are provided as a Source Data file.

(b) The GPSM initially identified by in silico deglycosylation (top) and the GPSM that passed the score threshold in the spectrum expansion (bottom).

(c) Normalized peaks of peptide part of intact glycopeptides shown in (b). Peptide fragmentation pattern was retained after spectrum expansion: (y11+HexNAc) ion is always the most abundant ion and followed by (y15+HexNAc) ion.

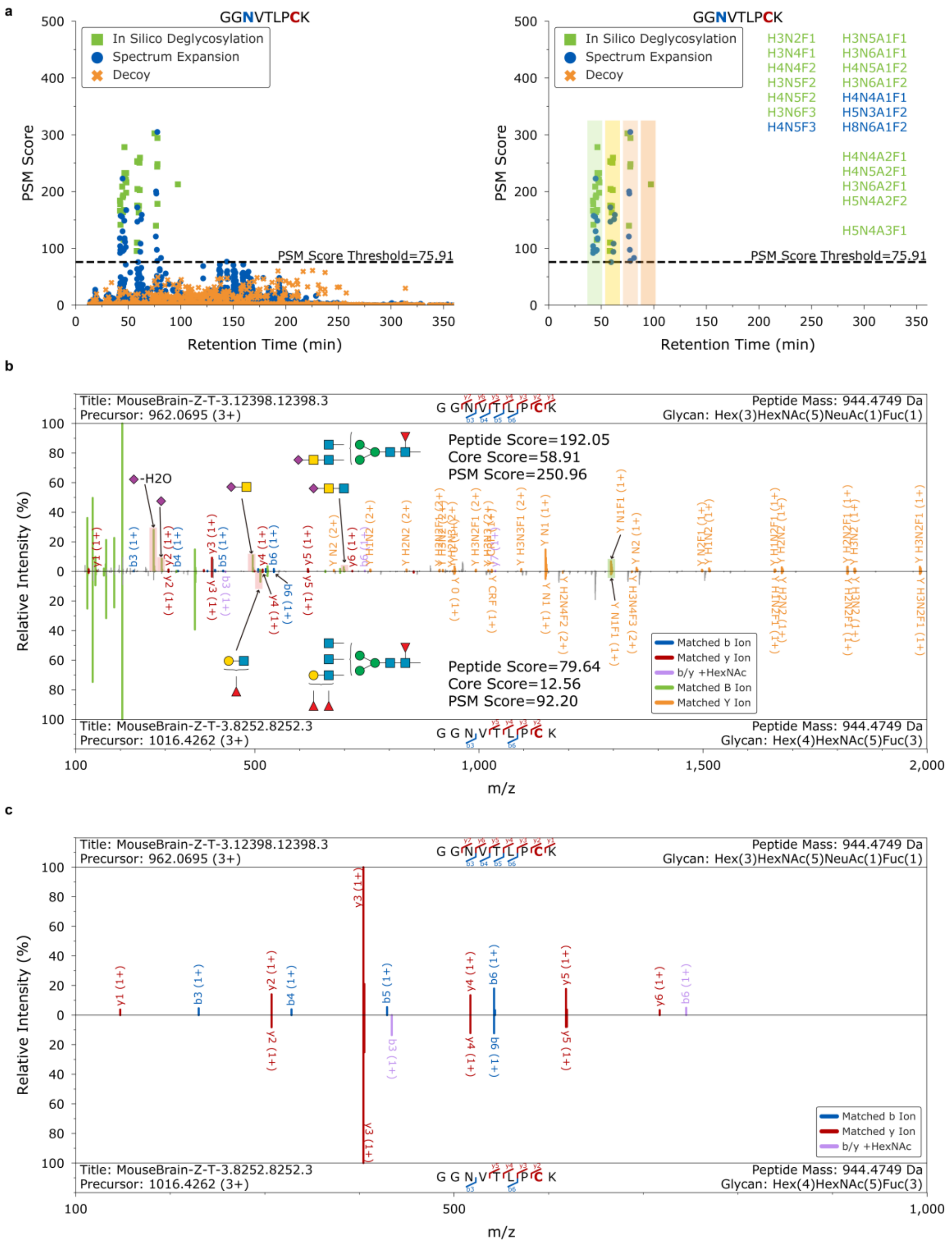

Supplementary Figure 9 Examples of GPSMs with peptide backbone "GGNVTLPCK" identified from in silico deglycosylation and spectrum expansion. (a) Score distribution of peptide-spectrum matches in spectrum expansion. Left: score distribution of all target-decoy PSMs obtained in spectrum expansion. Right: score

distribution of PSMs after score filtering and core structure peak matching. And the PSM score threshold was derived from the e-value filtration method. Source data are provided as a Source Data file.

(b) The GPSM initially identified by in silico deglycosylation (top) and the GPSM that passed the score threshold in the spectrum expansion (bottom).

(c) Normalized peaks of peptide part of intact glycopeptides shown in (b). Peptide fragmentation pattern was retained after spectrum expansion: y3 ion is the most abundant ion and other peptide ions are relatively low abundance.

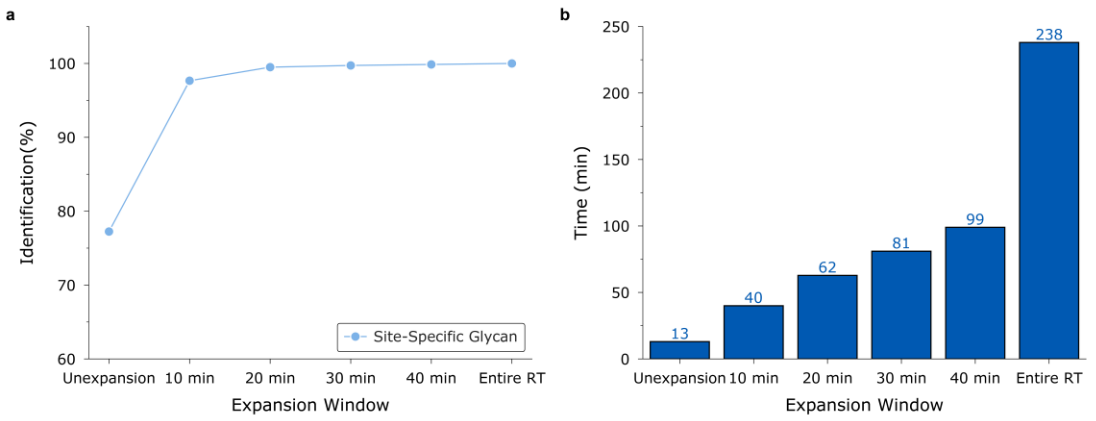

Supplementary Figure 10 Comparison of the performance of Glyco-Decipher with different expansion time windows on the brain data in the dataset of mouse tissues. (a) Identification performance of spectrum expansion with different ranges of window compared to the site-specific glycans identified with entire retention time expansion. (b) Comparison of the time cost for the identification of per file in the dataset of mouse brain when searching with different expansion time windows.

In comparison with entire retention time window, searching with 30 min expansion window only cost 1/3 time for the identification of the brain data in the mouse dataset and over 99.5% (99.73%) site-specific glycans were covered. In this work, the time window of 30 min was adopted if not otherwise stated. In Glyco-Decipher, the size of the expansion time window is a configuration setting and could be changed by users to an absolute value (second/min/hour) or a relative value (relative to the entire data acquisition time) for different liquid chromatography separations.

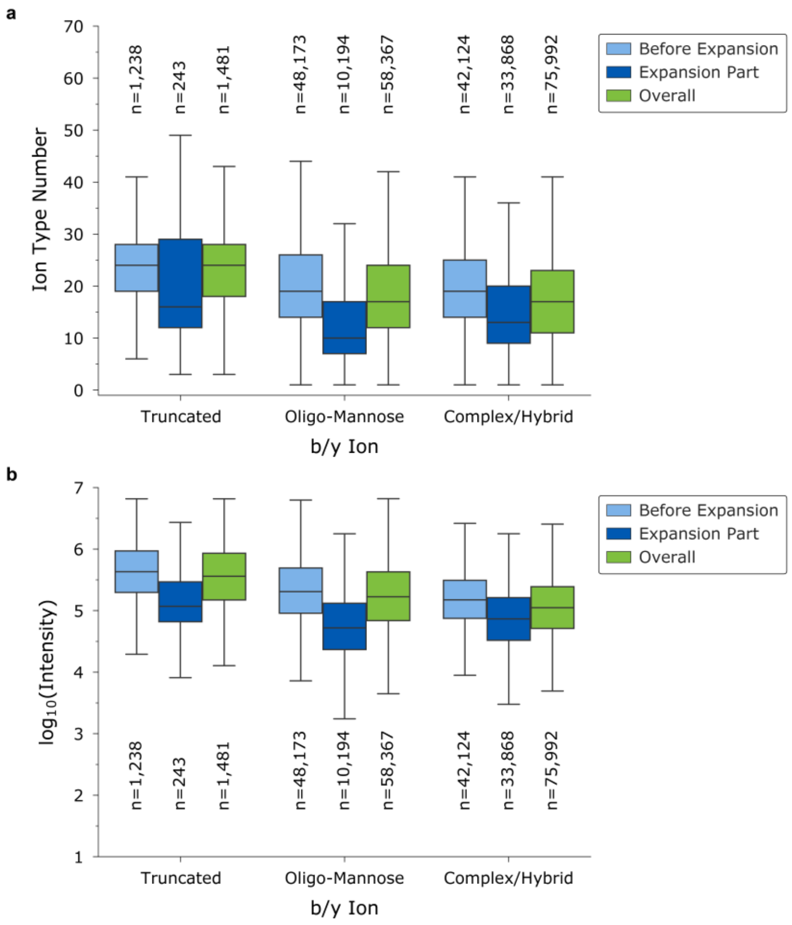

Supplementary Figure 11 Statistics of b/y ions in the PSM identification results originate from in silico deglycosylation and spectrum expansion.

Comparison of (a) number and (b) intensity of peptide b/y ions between in silico deglycosylation identification PSMs (pale blue) and spectrum expansion part PSMs (blue). The boxes show interquartile ranges (IQR), including median (middle line) and 25th/75th percentile (box), and whiskers indicate 1.5 ×IQR values; no outliers are shown and the spectrum numbers are labeled in the plot. Source data are provided as a Source Data file.

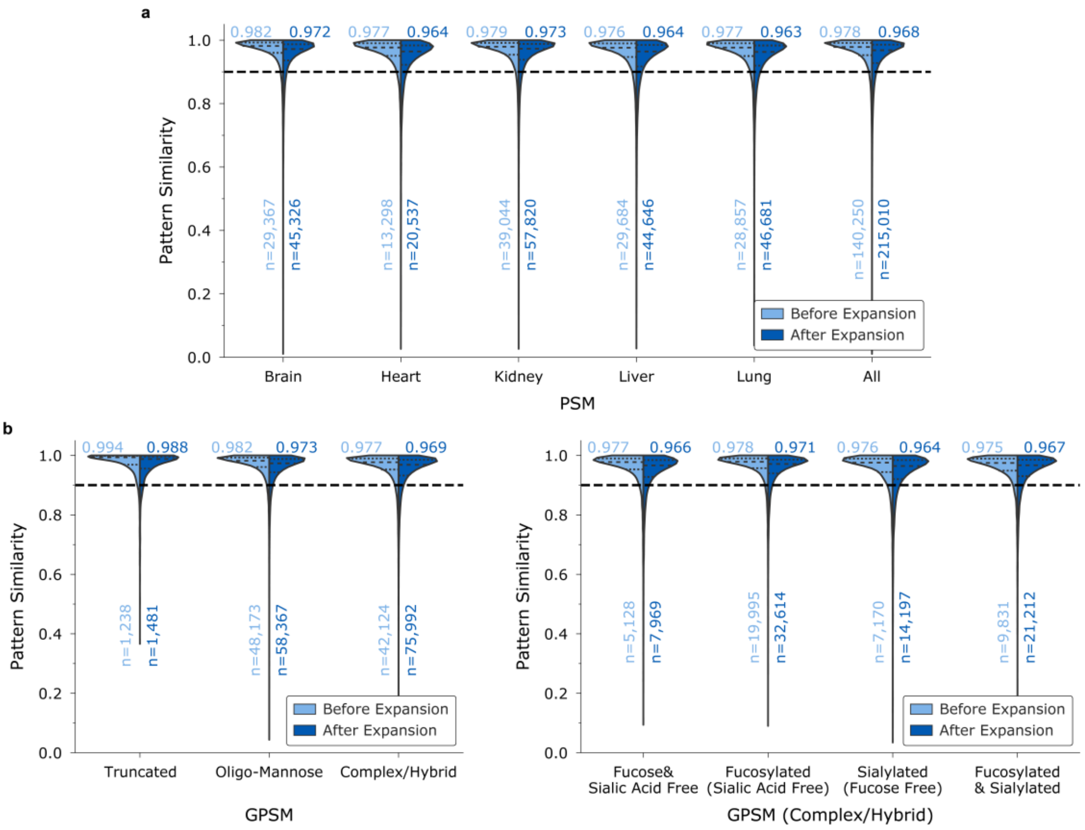

Supplementary Figure 12 Distribution of peptide fragmentation pattern similarities in (a) PSM and (b) GPSM results after spectrum expansion.

(a) By utilizing spectrum expansion strategy, peptides in 74,760 additional glycopeptide spectra were uncovered and resulted in a total of 215,010 PSM identifications. Distributions of fragmentation pattern similarities in spectrum expansion results (blue) are nearly unchanged compared to in silico deglycosylation results (pale blue) in five mouse tissue datasets, indicating high confidence of this method. The quartiles of the distributions are indicated by inner dashed lines. The medians and the spectrum numbers are labeled in the plot. The similarity value of 0.9 is indicated by outer dashed lines. Source data are provided as a Source Data file.

(b) After matching with GlyTouCan database, 91,535 and 135,840 GPSMs were

identified after peptide identification in in silico deglycosylation (before expansion, pale blue) and spectrum expansion (after expansion, blue). The fragmentation patterns of peptide backbones were retained in the results of spectrum expansion method. The quartiles of the distributions are indicated by inner dashed lines. The medians and the spectrum numbers are labeled in the plot. The similarity value of 0.9 is indicated by outer dashed lines. Source data are provided as a Source Data file.

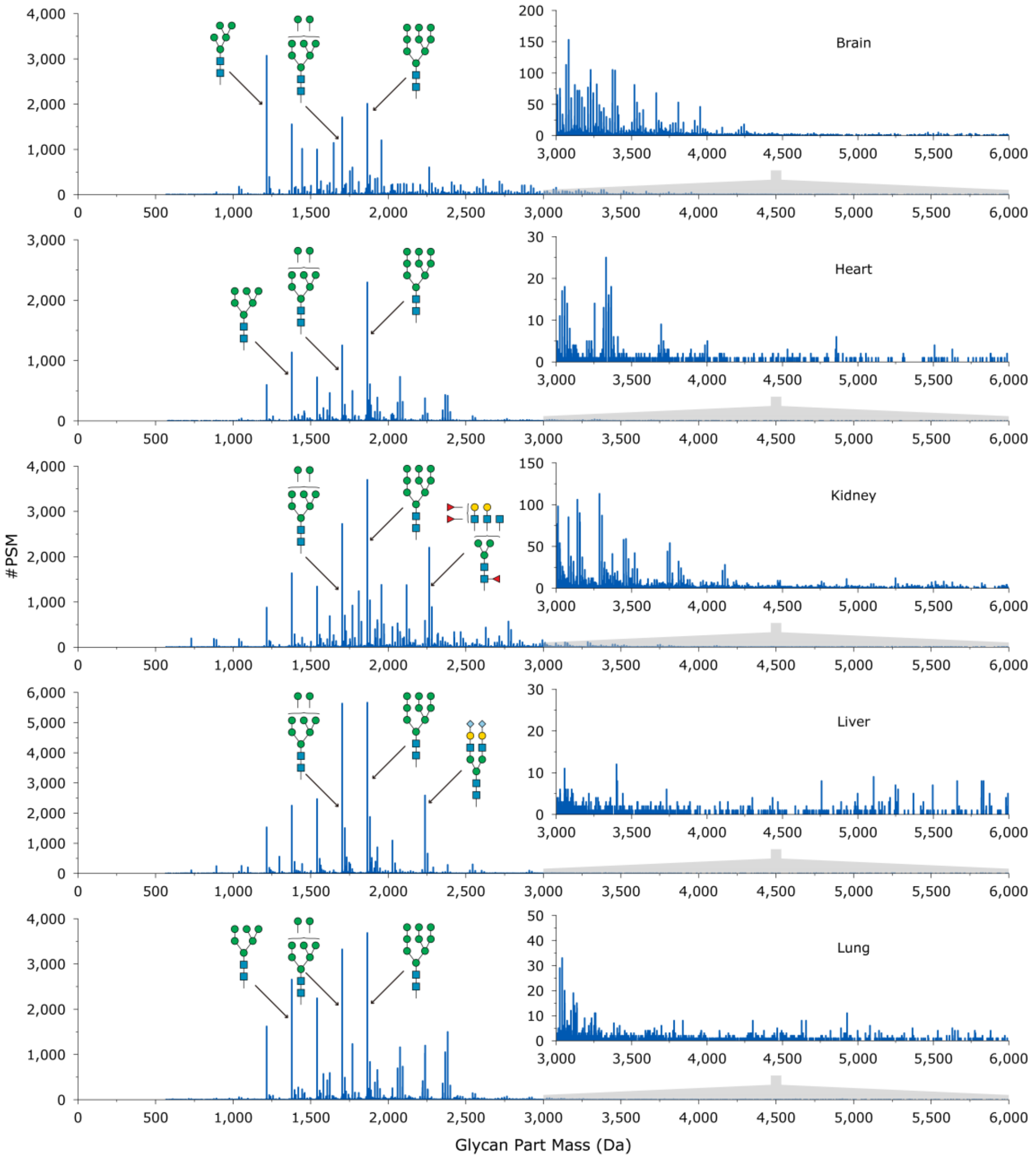

Supplementary Figure 13 Histogram of glycan part masses in the datasets of five mouse tissues.

The glycan part mass values were deduced after peptide part identification and precursor correction. All PSM identifications were validated by fragment ions of N-glycan core structure since corresponding ion matching was performed at the identification stages of in silico deglycosylation and spectrum expansion. So the mass values of glycans in a sample could be profiled without the use of any glycan database.

The glycan part profiles in mouse tissues (brain, heart, kidney, liver and lung) were listed and the glycan compositions of the three most abundant mass values were annotated. Bin width was set to be 1 Da and centered at integer values.

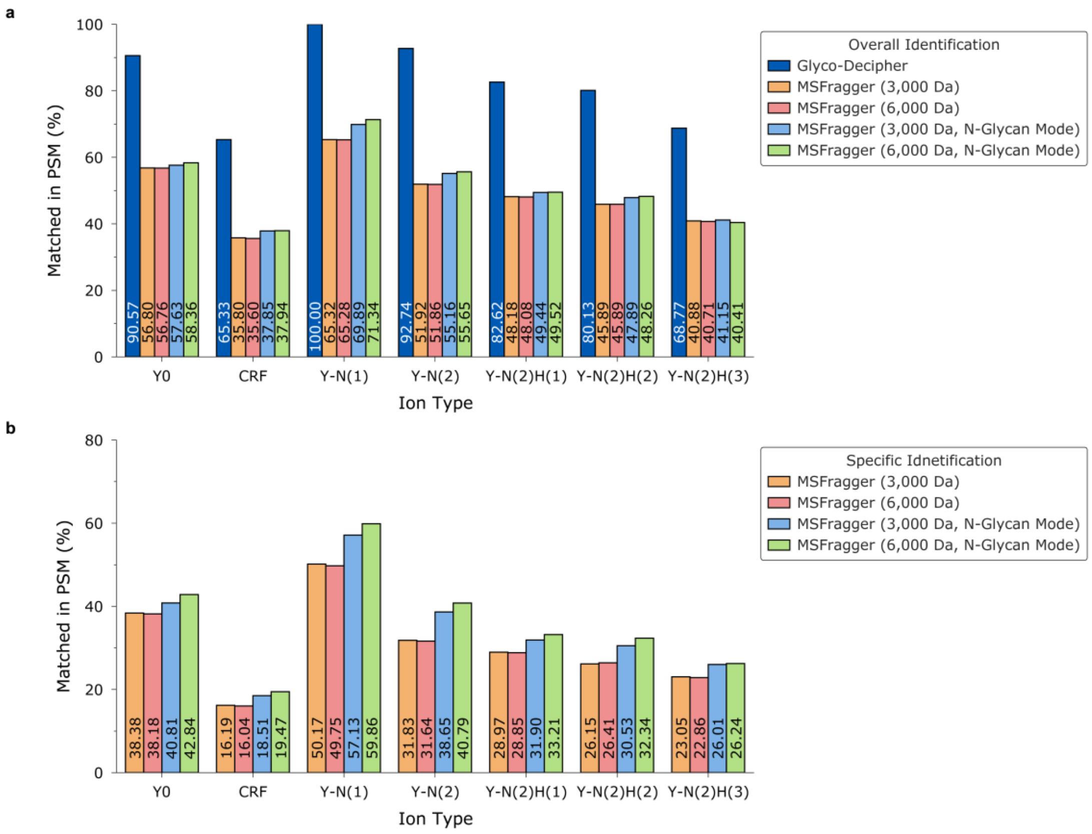

Supplementary Figure 14 Investigation of the Y ions of N-glycan core structure in PSMs identified by Glyco-Decipher and MSFragger.

(a) Percentage of PSMs in which the core structure Y ions were able to be matched based on the peptide results of Glyco-Decipher and MSFragger.

(b) Percentage of PSMs in which the core structure Y ions were able to be matched based on the results that specifically identified in MSFragger (including different identification of common spectra and different spectra).

CRF indicates the Y1 ion with cross ring fragmentation.

Y1 (peptide + HexNAc) ions are usually of high intensity in the low energy collision induced dissociation (CID) of glycopeptides[1]. It is also reported that the optimal range of collision energy for Y1 ions production is between 15% and 25%[2] or under 35%[3] in

stepped-energy higher-energy collisional dissociation (HCD), which is highly overlapped with the energy range used in this dataset[2] (collision energy=30%; stepped collision mode on with energy difference of ±10%). To investigate the peptide identification in Glyco-Decipher and MSFragger, theoretical Y ions of N-glycan core structure (charge state: +1/+2/+3) were deduced based on the peptide identifications of the two software tools and were matched in the corresponding glycopeptide spectra. In comparison with open search provided by MSFragger with different parameters, more peptide identifications of Glyco-Decipher matched corresponding core structure Y ions in glycopeptide spectra. Due to the core structure peak validation after spectrum expansion, all peptide identifications in Glyco-Decipher matched corresponding Y1 ions in MS2. Yet only about 60% (traditional mode) or 70% (N-glycan mode) PSMs matched to corresponding Y1 ions in the results of MSFragger (a) and this value decreased to 50-60% for the specific identification results of MSFragger (b).

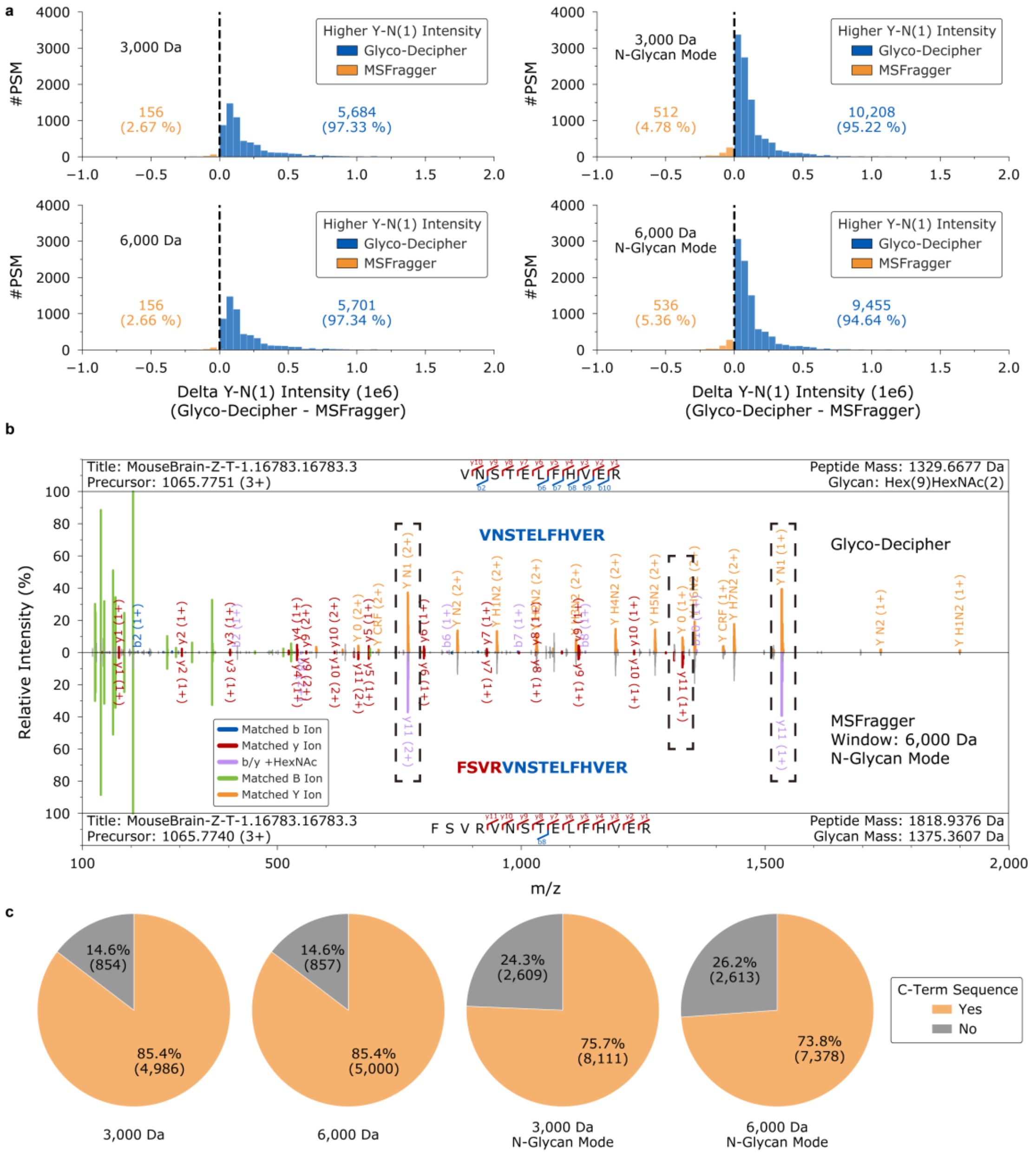

Supplementary Figure 15 Analysis of the glycopeptide spectra that were inconsistently identified in Glyco-Decipher and MSFragger.

(a) Distributions of intensity differences of the Y1 ion based on the distinct peptide identifications in Glyco-Decipher and MSFragger. Intensity of Y1 ion was set to be zero if not matched in the spectra. Y1 ions with higher intensity were matched in most of the glycopeptide spectra (>95%) based on the peptide results of Glyco-Decipher. Source data are provided as a Source Data file.

(b) Spectrum example to demonstrate the incorrect peptide matching of MSFragger. The peptide identified by Glyco-Decipher, “VNSTELFHVER”, is the C-term sequence part of “FSVRVNSTELFHVER”, which was identified by MSFragger. Ten y ions are shared by the two sequences (y1-y10) and were matched in both Glyco-Decipher and MSFragger. Yet glycan ions, including Y0 ion (peptide) and Y1 ion (peptide+HexNAc), were matched as peptide fragment ions incorrectly in MSFragger due to its wider MS1 mass window in open search mode, resulted in the incorrect peptide identification and the lack of corresponding core structure ions in the spectrum.

(c) Analysis of the peptide sequence of the spectra that were inconsistently identified by Glyco-Decipher and MSFragger. In 75-85% of the inconsistently identified glycopeptide spectra, the peptides of Glyco-Decipher are at the C-term of the peptide results of MSFragger.

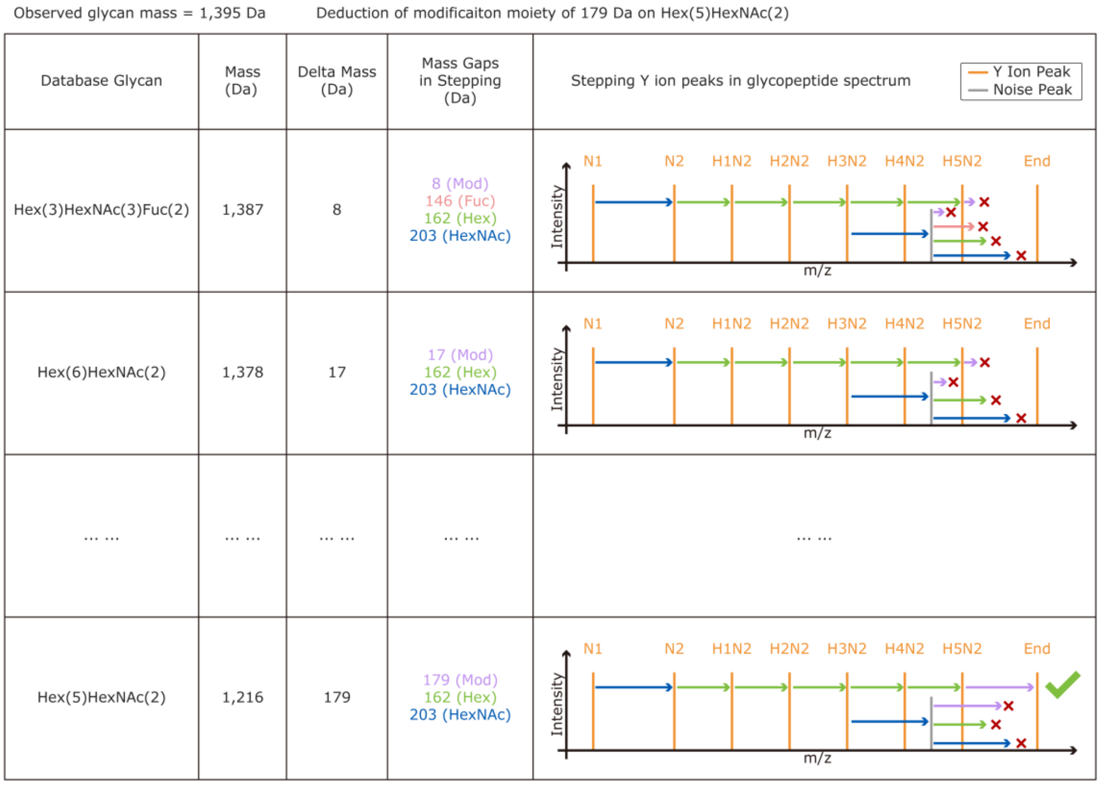

Supplementary Figure 16 Detailed interpretation of monosaccharide stepping method in unveiling modification moieties on glycans.

In this example, a modification moiety of 179 Da happens on the glycan Hex(5)HexNAc(2) can generate a modified glycan with mass values of 1,395 Da that does not match any database entries. By calculating mass differences between this unexpected glycan and database glycans, potential modification moieties on the database glycans (e. g., a modification of 8 Da on Hex(3)HexNAc(3)Fuc(2)) were enumerated. A list of stepping gaps was generated based on the mass difference and the types of monosaccharide in the database glycan and the gaps were used to stepwise match Y ions in glycopeptide spectra. Incorrect modification moieties were filtered out for failing to step the Y ions to the terminal, which is corresponding to the intact

glycopeptide. Notably, random matched peaks (e.g., a HexNAc step after Y-H3N2 ion in this figure) would also generate incorrect stepping route that could not reach to the stepping terminal.

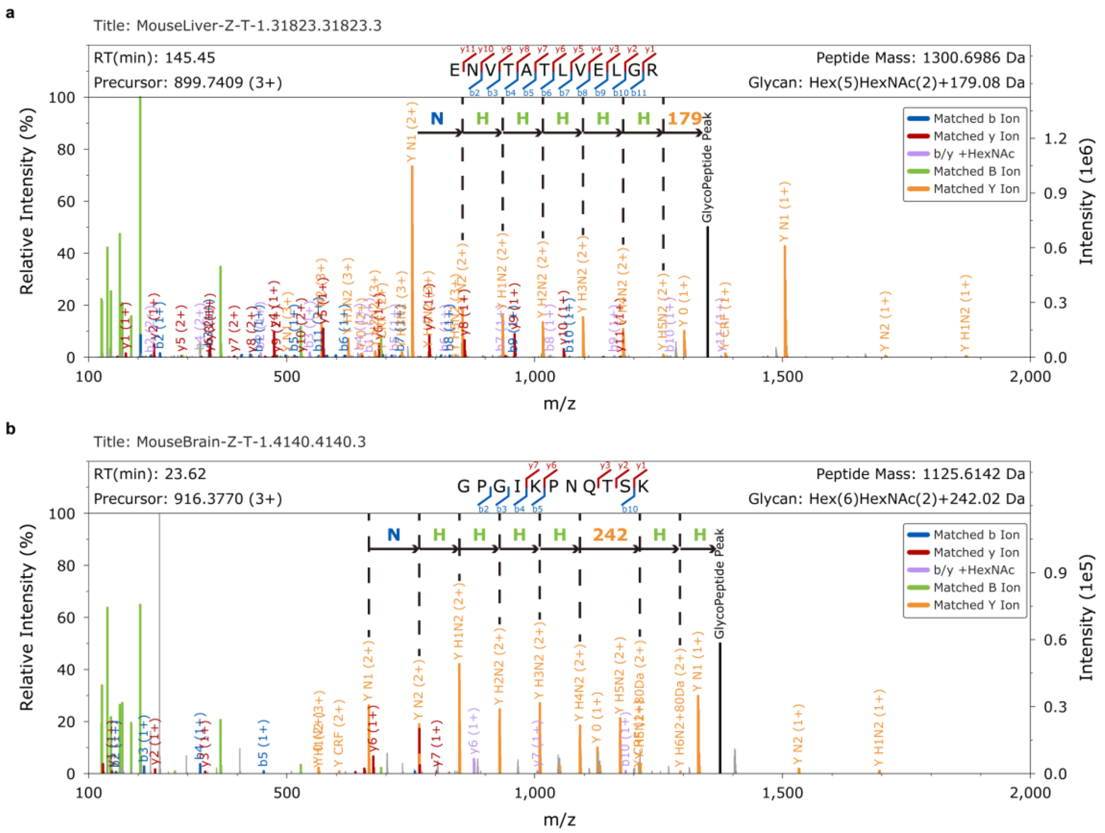

Supplementary Figure 17 Examples of monosaccharide stepping method to discover modification moieties on unmatched modified glycans.

Moieties with mass of (a) 179 Da and (b) 242 Da were deduced by stepping Y ions from the fragmentation of modified glycans that does not match to any entries in GlyTouCan database.

Monosaccharide abbreviation: H: Hex; N: HexNAc.

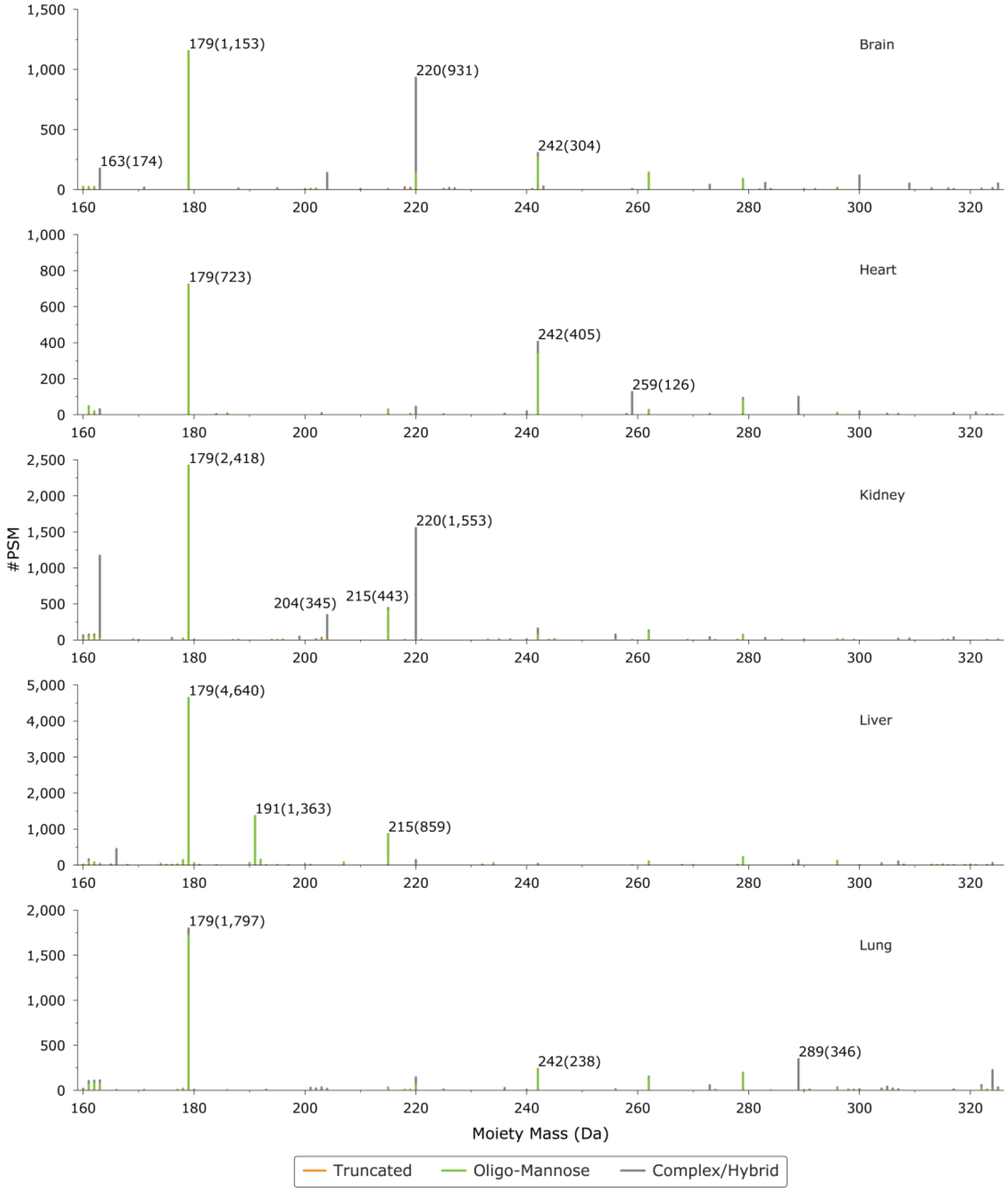

Supplementary Figure 18 Histogram of deduced mass values of modification moieties on modified glycans in five mouse tissue datasets.

Bar color indicates the type of glycan on which the modification linked and number annotation on each bar indicates mass value and GPSM number of corresponding modification moiety.

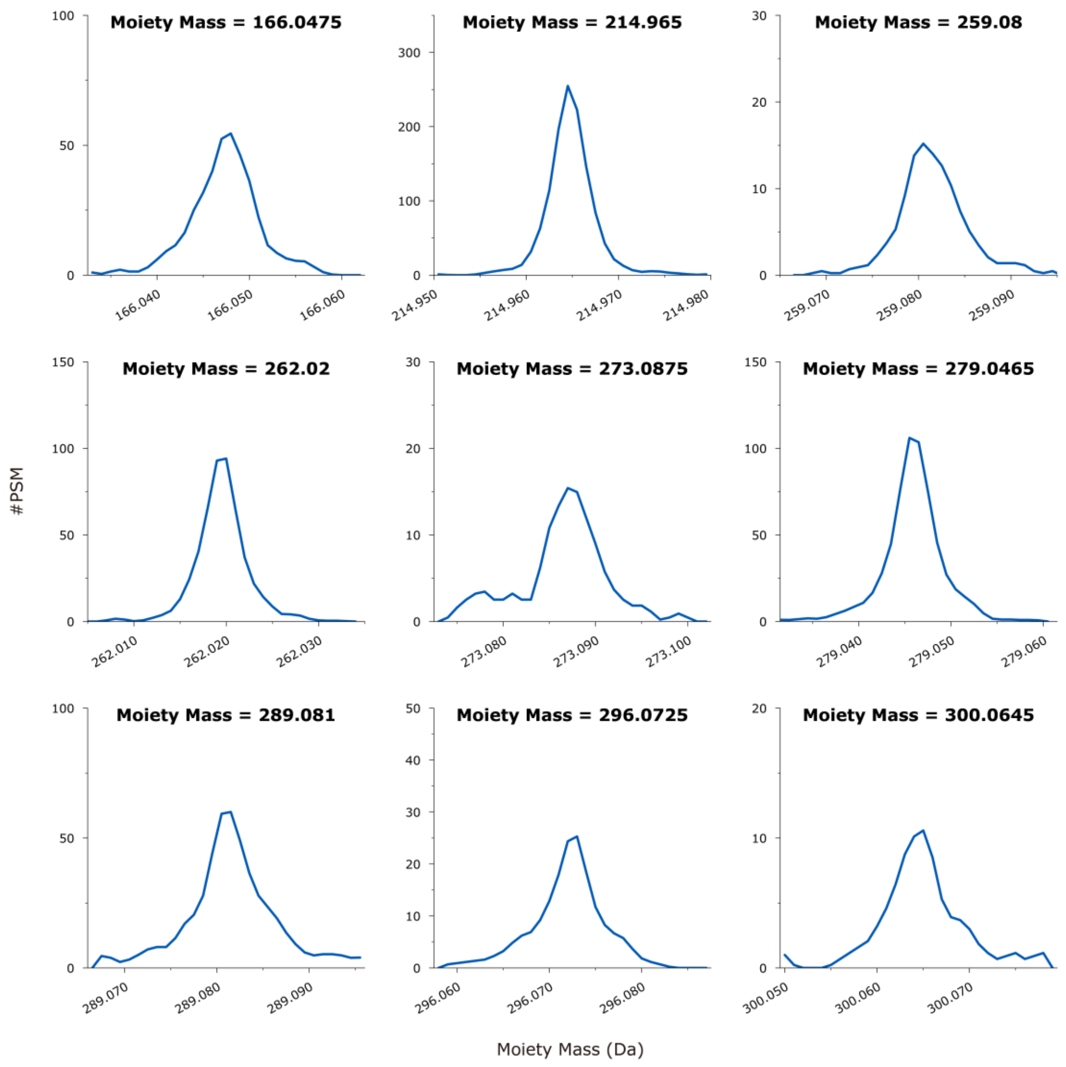

Supplementary Figure 19 Mass profile examples of modification moieties linked on modified glycans in five mouse tissues.

Source data are provided as a Source Data file.

a

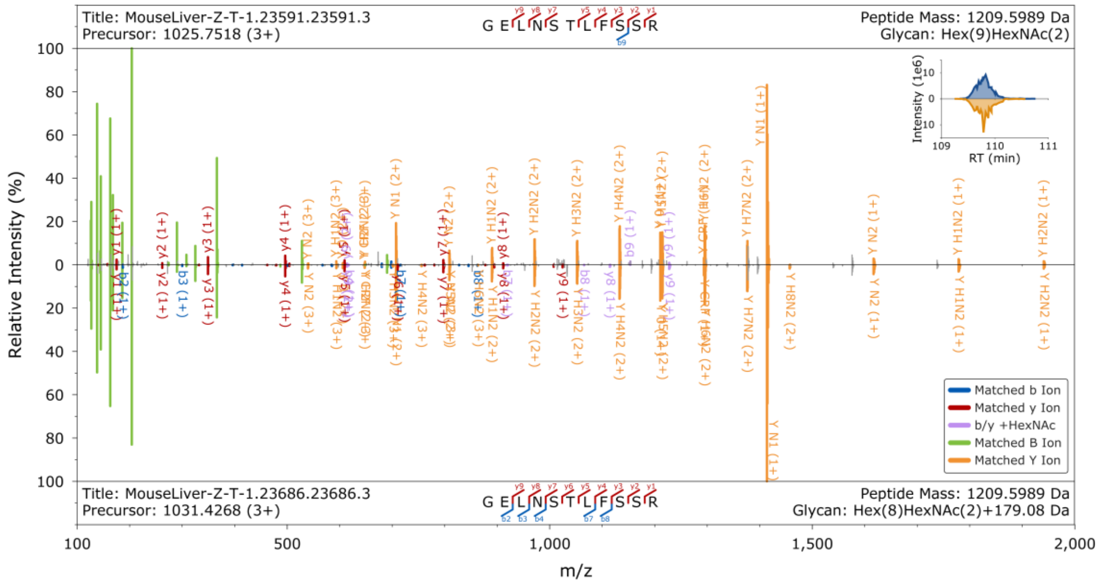

b

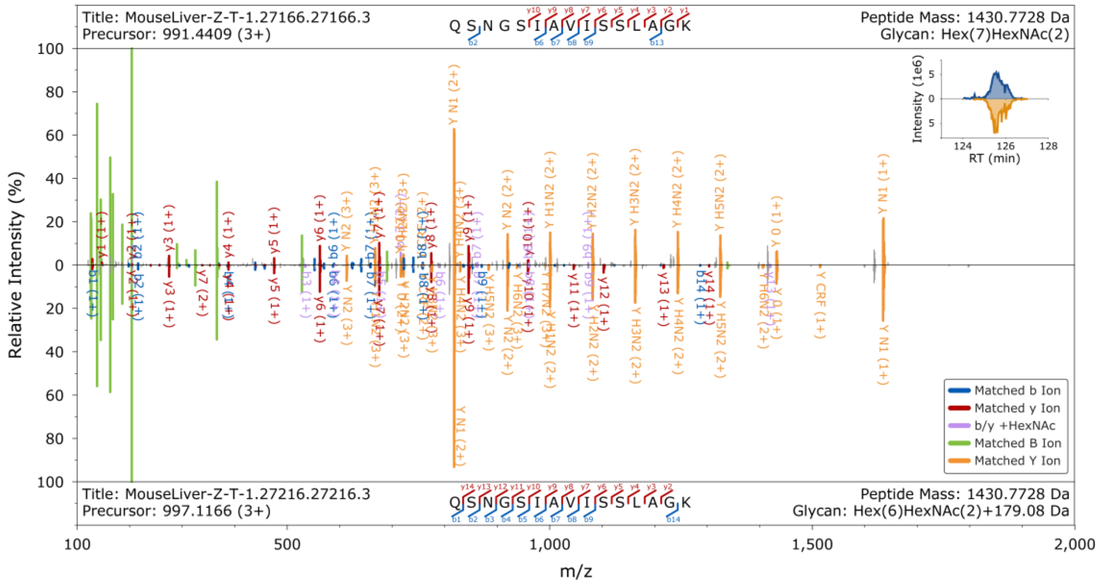

Supplementary Figure 20 Spectrum comparison between glycopeptides with oligo-mannose glycan and their counterparts modified by 179 Da moiety.

The spectrum of two glycopeptides with oligo-mannose glycan were annotated and compared to their 179 Da modified counterpart. The peptide sequence for the two glycopeptides were (a) “GELNSTLFSSR” (b) “QSNGSIAVISSLAGK”. Elution profiles of the two glycopeptides were shown in top right.

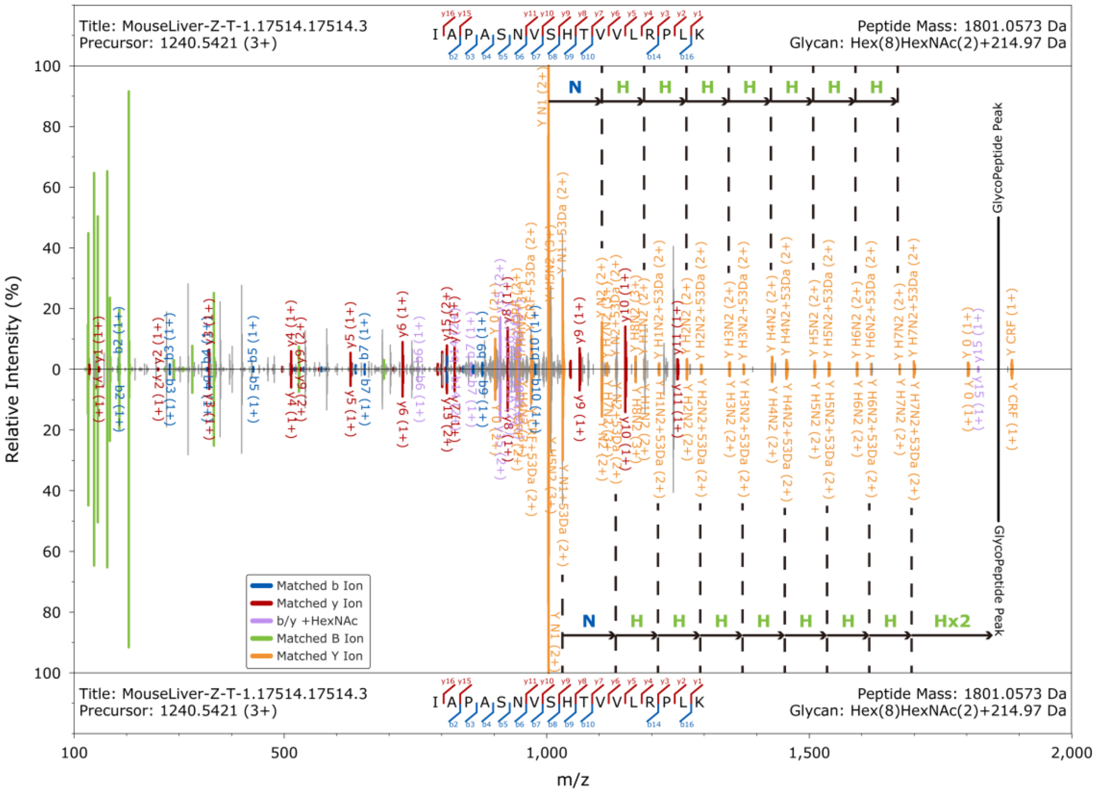

Supplementary Figure 21 Spectrum of glycopeptide deduced with a 215 Da modification moiety (Hex +53 Da).

The spectrum was plotted in a mirror mode. According to the glycopeptide spectrum, two series of Y ions were matched with the mass shift of 53 Da. The 53 Da difference between the two matched series of Y1 ions infers the displacement of three protons by iron ion (3+) may happen on glycopeptide.

Monosaccharide abbreviation: H: Hex; N: HexNAc.

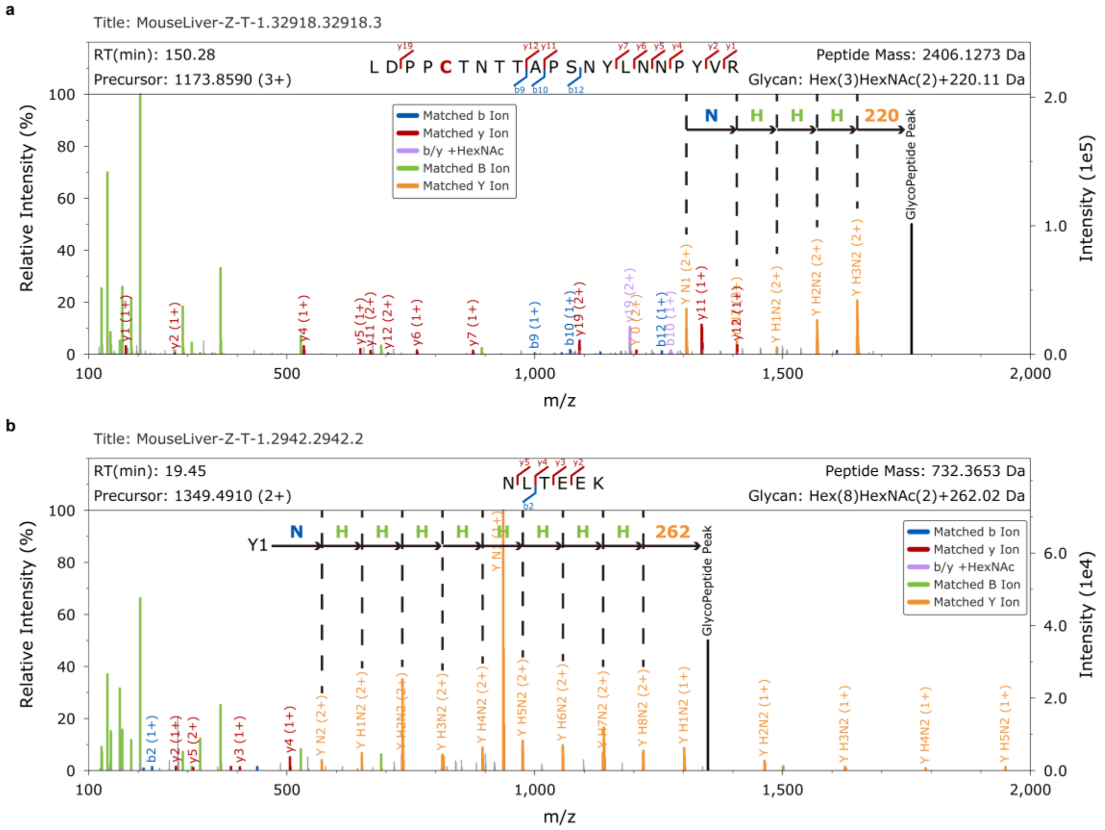

Supplementary Figure 22 Spectrum examples of glycopeptides with unannotated (a) 220 Da and (b) 262 Da modification moiety on glycan part.

Monosaccharide abbreviation: H: Hex; N: HexNAc.

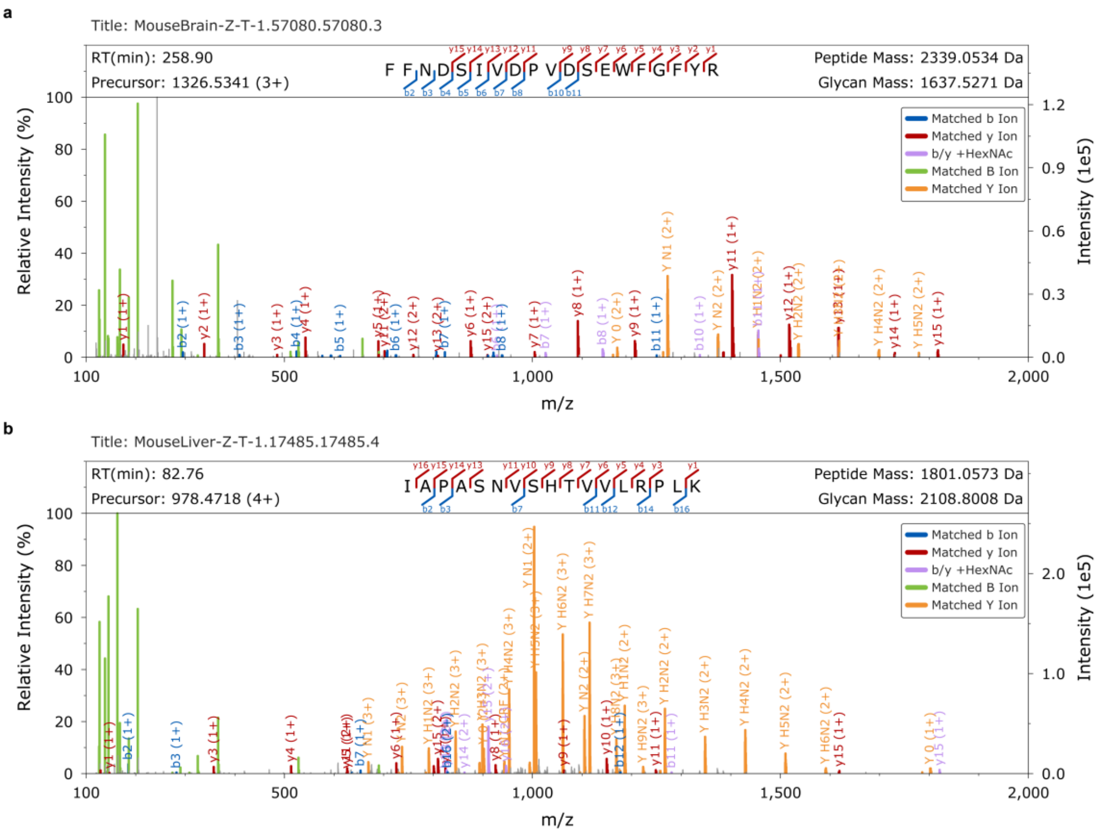

Supplementary Figure 23 Spectrum examples of glycopeptides with unannotated glycan part of (a) 1637.53 Da and (b) 2108.80 Da.

After annotating the glycan composition with database matching and by monosaccharide stepping, many PSMs with confident peptide sequence matching were left with unannotated glycan. To annotate their spectra, the experimental peak list was matched against theoretical peak list of identified peptide backbones, and Y ion peaks were matched by monosaccharide stepping from theoretically deduced Y1 (Y-HexNAc) ions.

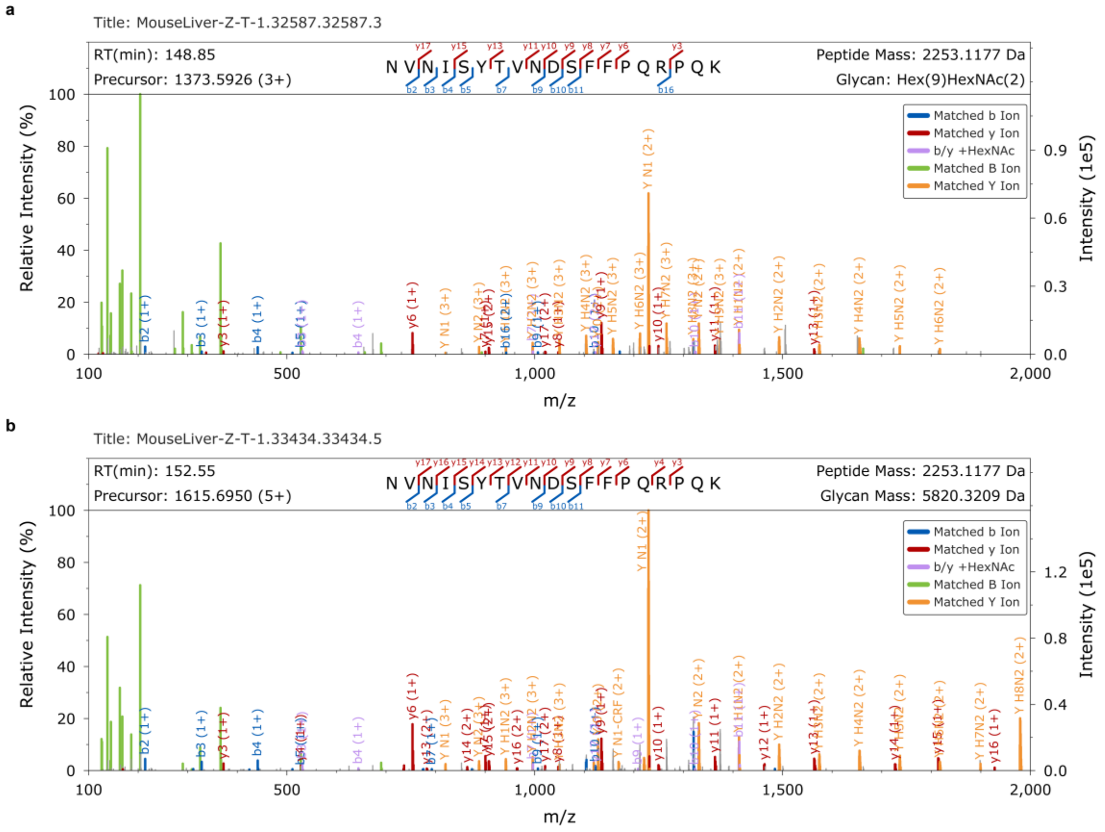

Supplementary Figure 24 Spectrum examples of glycopeptides with the same peptide backbone "NVNISYTVNDSFFPQRPQK" and different glycan parts of (a) Hex(9)HexNAc(2) and (b) 5820.32 Da.

The glycan database-independent workflow enables the identification of glycosylation with high mass glycans. For the two glycopeptides shown above, the shared peptide sequence "NVNISYTVNDSFFPQRPQK" displays similar fragmentation patterns with y6 and y9 ions the most abundant ions in peptide fragments, while the attached glycan exhibit dramatic mass difference with 1,864 Da of Hex(9)HexNAc(2) in (a) and unannotated 5,820 Da in (b). Note that two potential glycosites exist in the peptide sequence and could be co-glycosylated by N-glycans concurrently, which is a potential reason for the high glycan mass. Among the 53,320 PSMs with glycan unannotated,

10,797 results are for glycans of high masses (>3,000 Da) and only 10% (10.2%, 1,106/10,797) of them are matched to peptides containing >1 potential glycosites, indicating that co-glycosylation on peptides is not the major reason of the high glycan masses. The huge difference in glycan parts suggests the high sensitivity of Glyco-Decipher in spectrum interpretation without the limitation of glycan part mass.

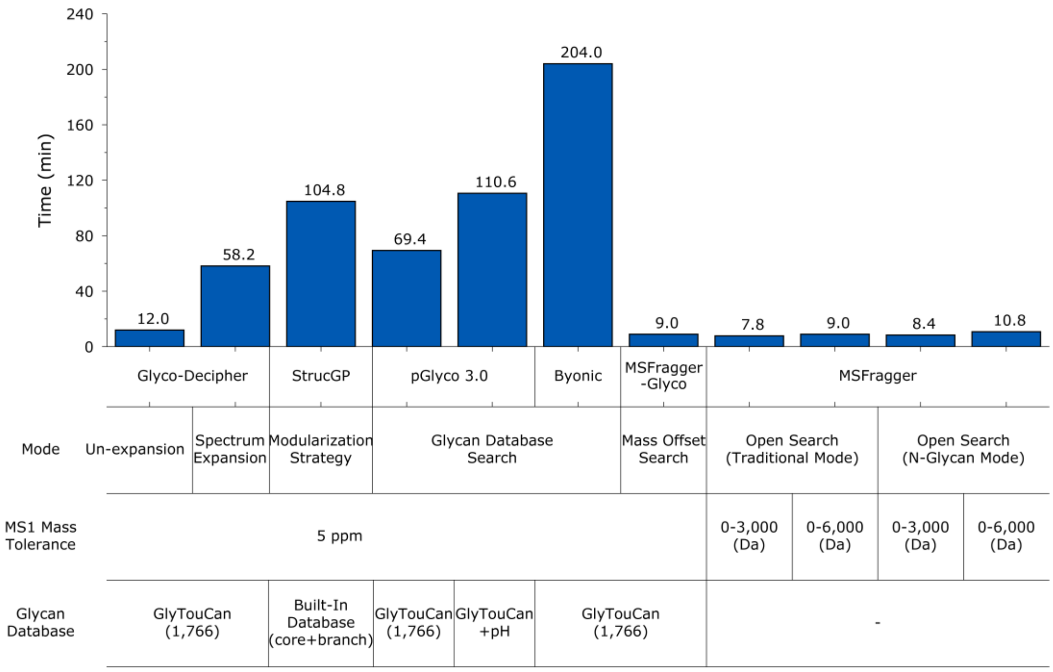

Supplementary Figure 25 Comparison of the average time required for the search of each raw file in the dataset of mouse tissues with different software tools.

(a) The average time required for the tools (Glyco-Decipher, StrucGP, pGlyco 3.0, Byonic, MSFragger-Glyco and its open search mode) to search per raw file in the dataset of mouse tissues. We also investigated the time consumption of different modes of Glyco-Decipher (un-expansion and spectrum expansion) and open search method provided by MSFragger with different parameters. The mass tolerance and the glycan database adopted in glycopeptide identification were identical in this comparison except for StrucGP for which its built-in database of core/branch structures was adopted. All searches were performed on a desktop computer running Windows 10 Pro (v.20H2), with two 2.20 GHz Intel Xeon E5-2650 v4 CPU processors with 64 Gb of installed RAM.

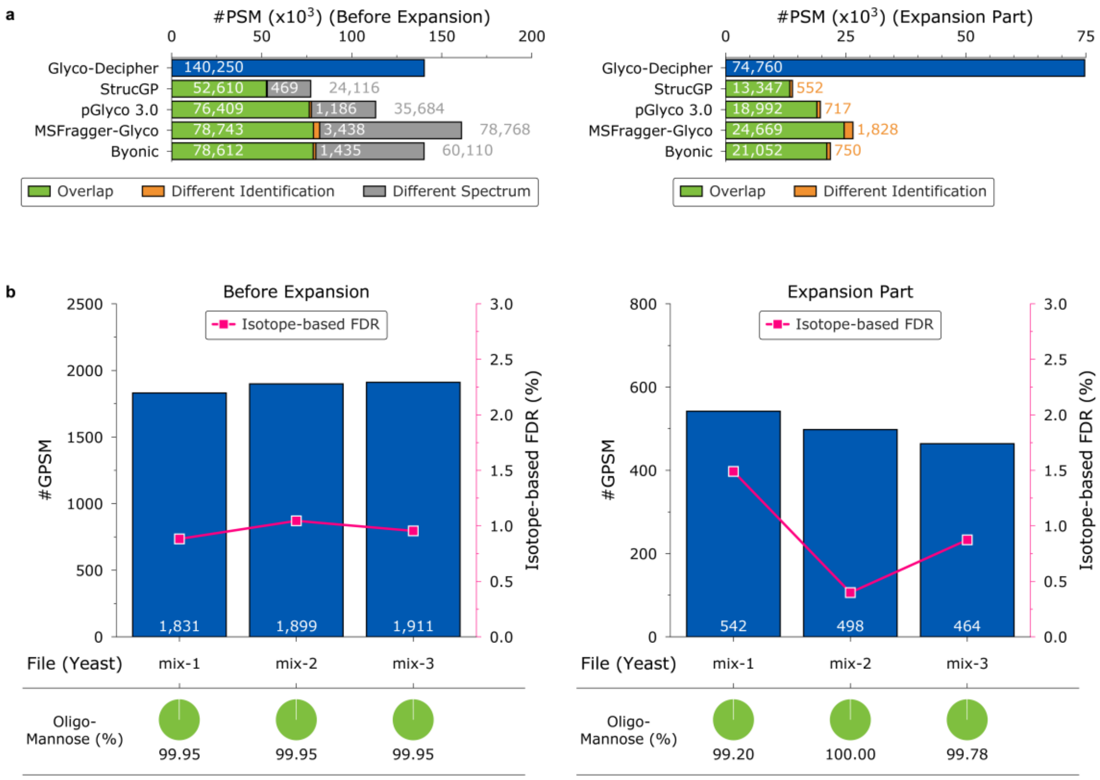

Supplementary Figure 26 Analysis of the identification results from in silico deglycosylation (before expansion) and spectrum expansion.

(a) Comparison of the PSM results achieved by in silico deglycosylation (before expansion, left) and spectrum expansion (expansion part, right) in Glyco-Decipher with other software tools on the dataset of mouse tissues.

(b) FDR analysis of the glycopeptide identification results from in silico deglycosylation (before expansion, left) and spectrum expansion (expansion part, right) on the dataset of $^{13}C/^{15}N$ metabolically labeled yeast dataset. The isotope-based FDR was calculated by matching $^{13}C/^{15}N$ isotopic peaks in MS1 with pQuant[4]. Source data are provided as a Source Data file.

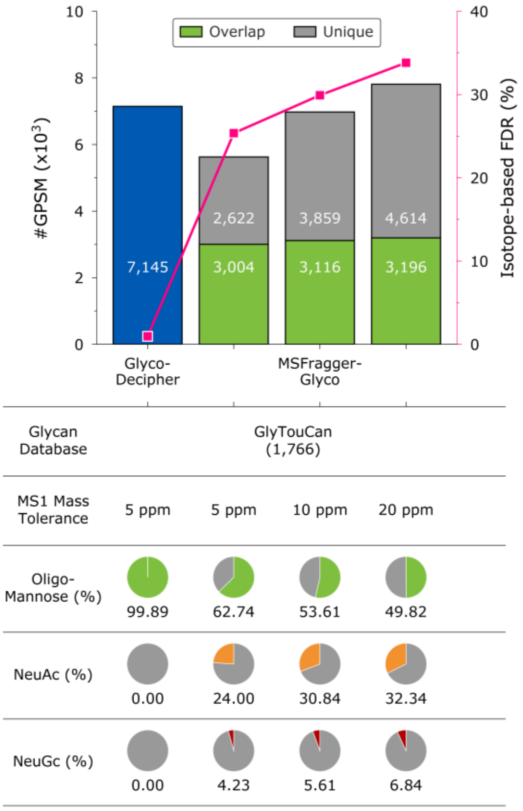

Supplementary Figure 27 FDR Analysis of MSFragger-Glyco with $^{13}C/^{15}N$ metabolically labeled yeast dataset under different MS1 mass tolerance.

The identification performance of MSFragger-Glyco with different MS1 parameter was investigated. Searching with different MS1 mass tolerance, including 5 ppm, 10 ppm and 20 ppm (20 ppm was adopted in the original publication of MSFragger-Glyco), were performed. We found that enlarging MS1 mass tolerance in searching would increase the identification number of MSFragger-Glyco to some extent, while it also elevated the identification error rate. Source data are provided as a Source Data file.

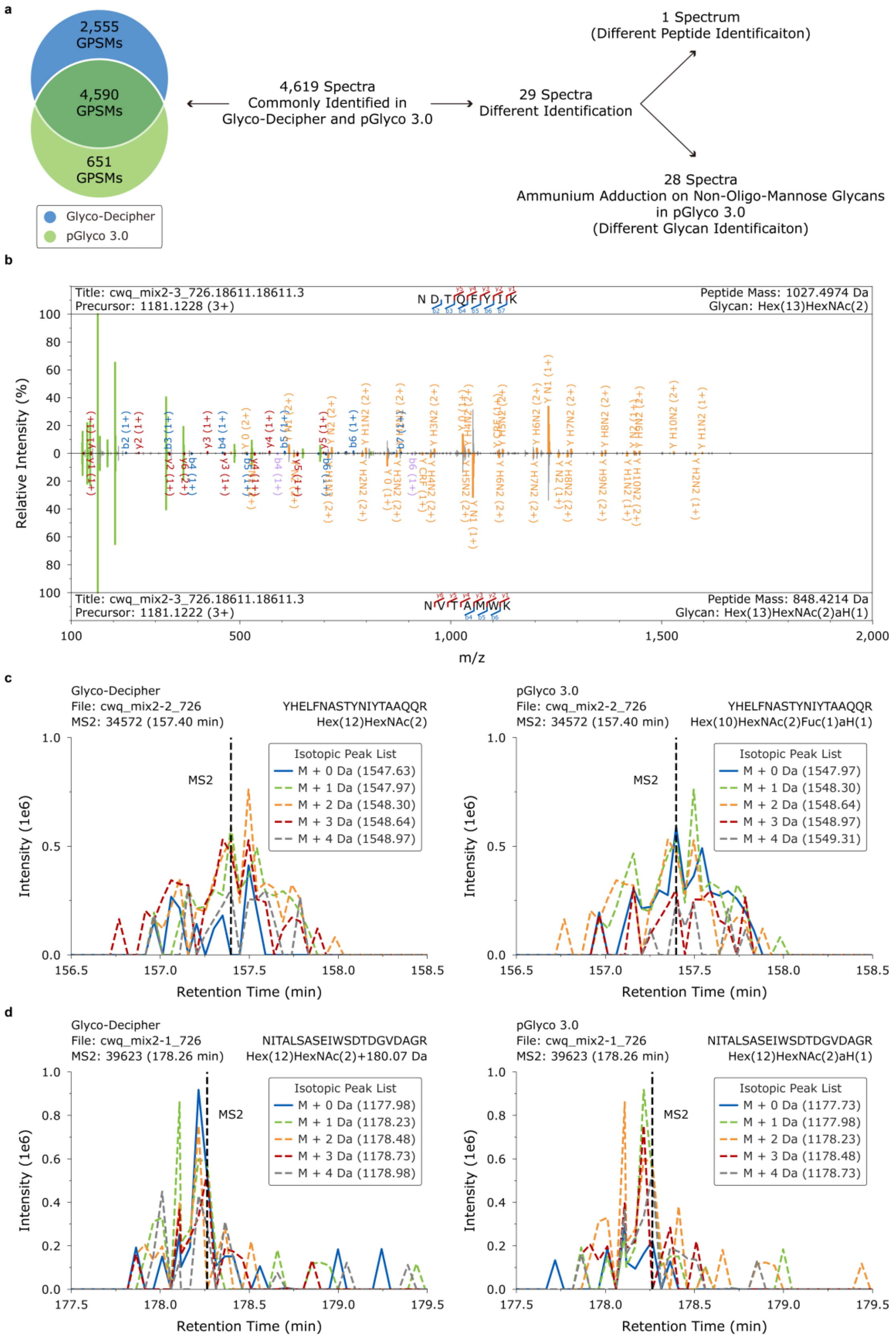

Supplementary Figure 28 Analysis of the identification results of Glyco-Decipher and pGlyco 3.0 on the dataset of yeast.

(a) Analysis of the 4,619 glycopeptide spectra that were commonly identified in Glyco-Decipher and pGlyco 3.0.

(b) Annotated glycopeptide spectrum of "cwq_mix2-3_726.18611.18611.3" that were matched to distinct peptide backbones in Glyco-Decipher and pGlyco 3.0.

(c) The MS1 extracted-ion chromatograms (XICs) of the precursor for spectrum "cwq_mix2-2_726. 34572.34572.3" that was differently identified in Glyco-Decipher (left) and pGlyco 3.0 (right). aH means ammonium adducted hexose in pGlyco 3.0 and "M" means the mono-isotopic peak of the precursor. Source data are provided as a Source Data file.

(d) The MS1 extracted-ion chromatograms of the precursor for spectrum "cwq_mix2-1_726.39623.39623.4" that was matched to the same glycopeptide in Glyco-Decipher (left) and pGlyco 3.0 (right). The precursor of the spectrum was assigned to the (mono-isotope +1 Da) value in Glyco-Decipher. Source data are provided as a Source Data file.

Based on bio-synthesis rules, only oligo-mannose glycans should be identified in the yeast dataset. 4,619 glycopeptide spectra were commonly identified by Glyco-Decipher and pGlyco 3.0 and 4,590 of them were matched to identical glycopeptide results (including results of GlyTouCan glycans and results of ammonium adducted glycans). Among the 29 spectra that were matched to distinct glycopeptides in Glyco-Decipher and pGlyco 3.0, only 1 of them was matched to different peptide sequences: the spectra is likely to be a chimeric spectra since sufficient peptide ions and glycan ions were matched in the spectra to support both identifications (as presented in (b)). For the other

28 spectra, the identification differences were introduced by non-oligo-mannose glycan identifications in pGlyco 3.0 while all of them were assigned with oligo-mannose glycans in Glyco-Decipher. Since the modified glycan identification in pGlyco 3.0 is achieved by enumerating all database entries with provided modification, the increased glycan search space introduced random matches. For example, as presented in (c), the glycan part was incorrectly matched to an ammonium adducted non-oligo-mannose glycan in pGlyco 3.0, because the mass difference between Fuc(1)aH(1) and Hex(2) is 1 Da and the precursor with poor isotopic pattern was assigned to the (mono-isotope +1 Da) value. In contrast, all the ammonium adducted glycans were reported to be oligo-mannose glycans by monosaccharide stepping method in Glyco-Decipher.

However, incorrect precursor assignments were also observed in Glyco-Decipher for MS1 spectra with poor quality. Among the results of Glyco-Decipher, the precursors of 18 spectra (0.25% out of the 7,145 GPSMs) were matched to (mono-isotope+1) values, resulting in the identification of oligo-mannose glycans but isotope-shifted modification part, e.g. ammonium adducted hexose were matched to the 180 Da moiety as presented in (d). And these results were excluded when compared with pGlyco 3.0 in this study.

The above analysis indicates that random matches of modified glycans are potential to be introduced by the enumeration method in pGlyco 3.0 due to the increase of candidate glycans in search spaces. Also, improvements for the detection of precursor, especially for those with inferior isotopic patterns in MS1, are needed for glycoproteomics tools.

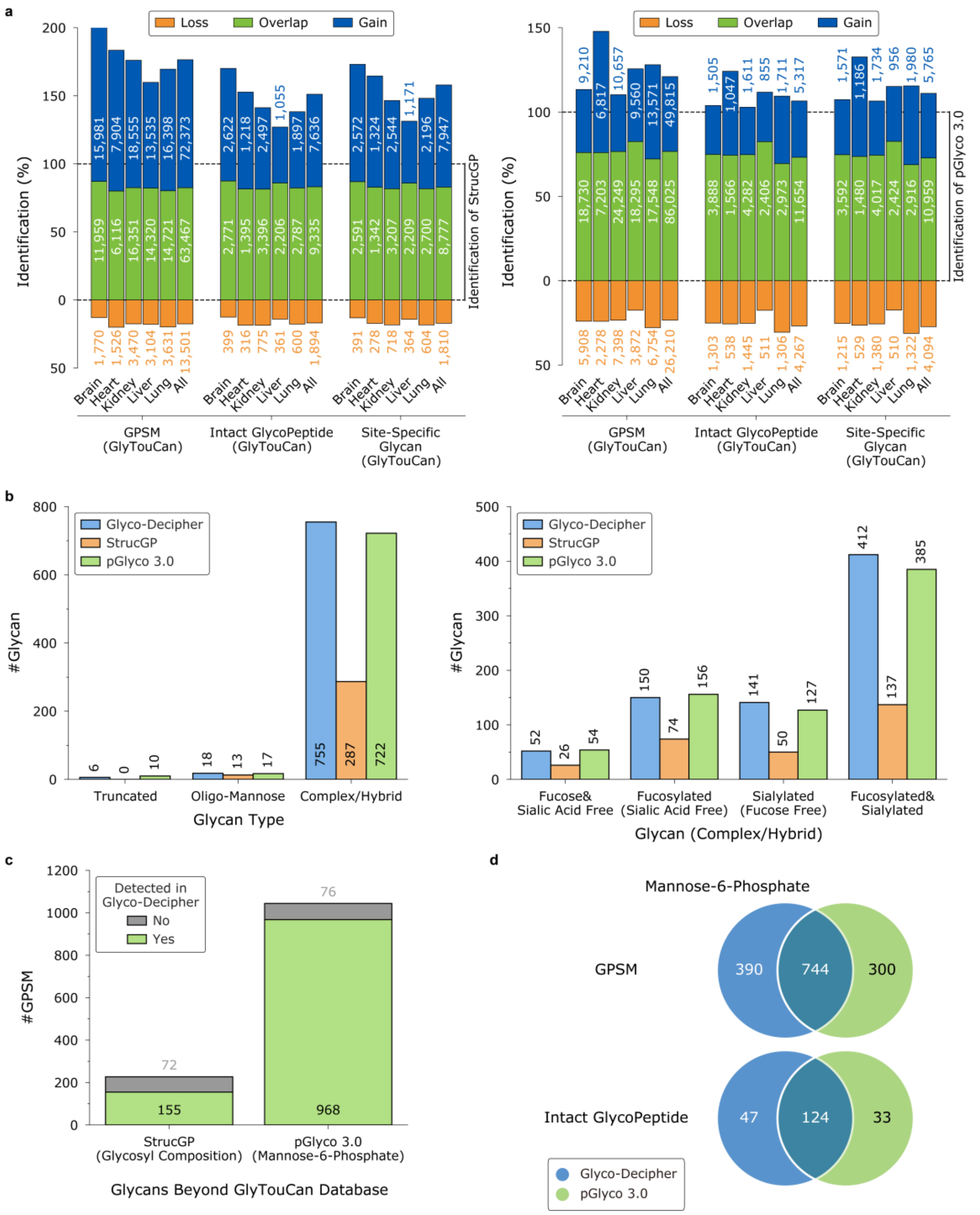

Supplementary Figure 29 Analysis of the identification results of Glyco-Decipher, StrucGP and pGlyco 3.0 on the dataset of mouse tissues.

(a) Identification performance evaluation of Glyco-Decipher in comparison with StrucGP (left) and pGlyco 3.0 (right) when only identification results of GlyTouCan glycans were considered. The additional (gain), overlap and lost identifications of

Glyco-Decipher compared to other tools are indicated by blue, green and orange bars, respectively. Compared to StrucGP, 76.5% more glycopeptide spectra and 58.0% more site-specific glycans were identified by Glyco-Decipher when the glycan search space was restricted to GlyTouCan database glycans. In comparison with pGlyco 3.0, identical glycan database (GlyTouCan) were adopted in glycopeptide identification, and increases of 21% and 11.1% in the number of identified glycopeptide spectra and site-specific glycans were provided by Glyco-Decpher.

(b) Comparison of the glycan identifications in different categories between Glyco-Decipher and StrucGP/pGlyco 3.0.

(c) Investigation of the glycopeptide spectra of unexpected glycans identified by StrucGP and pGlyco 3.0.

(d) Comparison of identification performance between Glyco-Decipher and pGlyco 3.0 in the identification of M6P glycosylation.

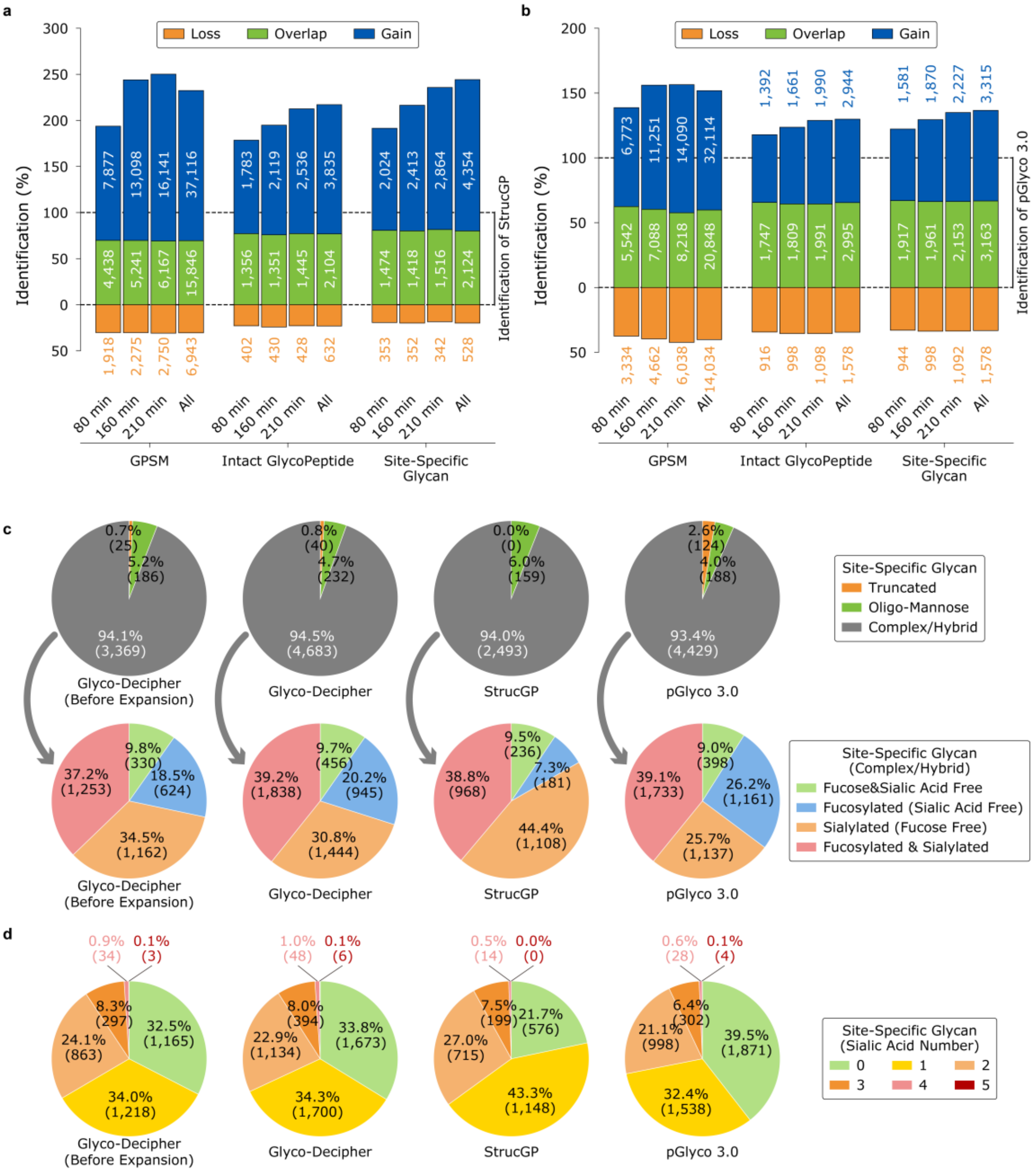

Supplementary Figure 30 Comparison of the identification performance between Glyco-Decipher and StrucGP/pGlyco 3.0 on the dataset of human serum.

(a) Comparison of identification performance between Glyco-Decipher and StrucGP. Featured with complex/hybrid glycans, especially sialic acid containing glycans, the dataset of human serum contains glycopeptide mass spectra acquired with LC separation gradients of 80, 160 and 210 min, respectively. The additional (gain), overlap and lost identifications of Glyco-Decipher compared to StrucGP are indicated by blue,

green and orange bars, respectively. In comparison with StrucGP, Glyco-Decipher identified 132.4% more glycopeptide spectra from the human serum sample separated with listed LC conditions. In total, an increase of 144.3% in the number of site-specific glycan identifications were yielded by Glyco-Decipher in human serum compared to StrucGP.

(b) Comparison of identification performance between Glyco-Decipher and pGlyco 3.0. The additional (gain), overlap and lost identifications of Glyco-Decipher compared to pGlyco 3.0 are indicated by blue, green and orange bars, respectively. In comparison with pGlyco 3.0, Glyco-Decipher enabled the spectrum interpretation for 51.8% more glycopeptide spectra, thus introducing an increase of 36.6% in the number of identified site-specific glycans.

(c) Distributions of the number of site-specific glycans in different categories revealed by Glyco-Decipher (before and after expansion, database glycan only), StrucGP and pGlyco 3.0.

(d) Distributions of the number of site-specific glycans with different number of sialic acids in the results of Glyco-Decipher (before and after expansion, database glycan only), StrucGP and pGlyco 3.0. Much more site-specific glycans of multi-sialylation were revealed by Glyco-Decipher after spectrum expansion. Note that most site-specific glycans with 5 sialic acid glycans were identified by Glyco-Decipher yet were lost by StrucGP.

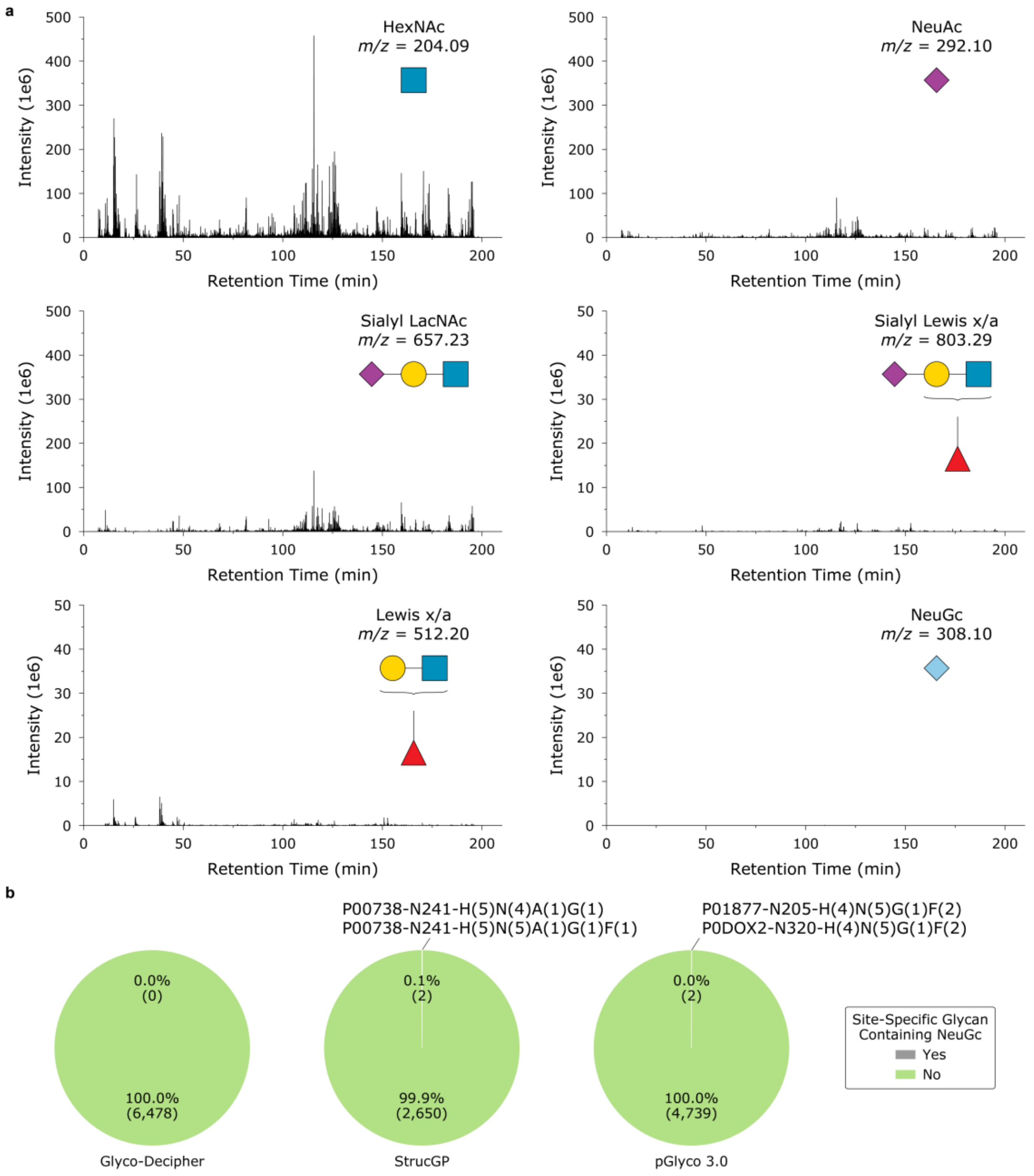

Supplementary Figure 31 Analysis of the glycan compositions in human serum.

(a) The XICs of diagnostic oxonium/B ions in the glycopeptide spectra of human serum. The extracted ion chromatograms were performed at the MS2 level. The XIC results suggested the presence of NeuAc and antenna fucosylation in the glycopeptides of human serum. In contrast, no diagnostic oxonium ions of NeuGc were observed in the dataset of human serum. All the mass spectrometry data with different LC gradients were considered for the XIC analysis and only data from 210 min LC gradient of were

plotted in this figure (similar patterns were observed in data with different LC gradients and were not shown).

(b) The distributions of site-specific glycans containing NeuGc in the results of Glyco-Decipher, StrucGP and pGlyco 3.0.

Monosaccharide abbreviation: H: Hex; N: HexNAc; A: NeuAc; G: NeuGc; F: Fuc.

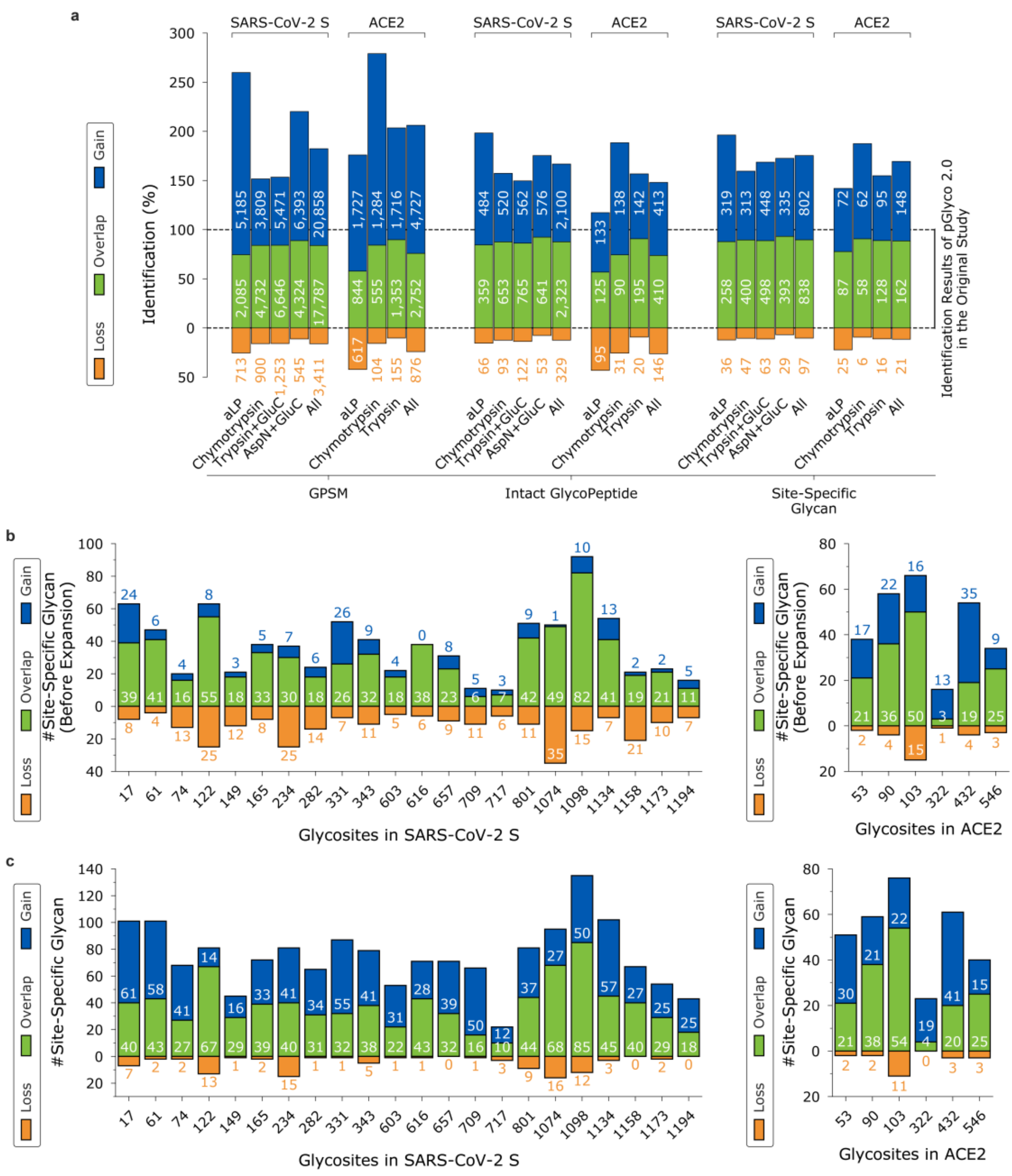

Supplementary Figure 32 Comparison of identification results of published protein glycosylation datasets of SARS-CoV-2 Spike and ACE2 between Glyco-Decipher and the original study.

(a) Comparison of the identification performance of Glyco-Decipher with original study[5] at the levels of GPSM, intact glycopeptide and site-specific glycan.

(b) Comparison of site-specific glycans on SARS-CoV-2 S (left) and ACE2 (right) between the results reported by Glyco-Decipher (before expansion) and in the original

study.

(c) Comparison of site-specific glycans on SARS-CoV-2 S (left) and ACE2 (right) between the results reported by Glyco-Decipher (after expansion) and in the original study. Much more site-specific glycans were recovered in the spectrum expansion stage compared to the in silico deglycosylation results. For example, on the site of N1158 in SARS-CoV-2 S, 40 site-specific glycans were reported in the original study and only 19 of them were detected by Glyco-Decipher before spectrum expansion, result in a loss of 21 site-specific glycan results. Yet spectrum expansion recovered all the lost results and provided information on 27 additional site-specific glycan identifications. The additional (gain), overlap and lost identifications of Glyco-Decipher compared to the original study are indicated by blue, green and orange bars, respectively.

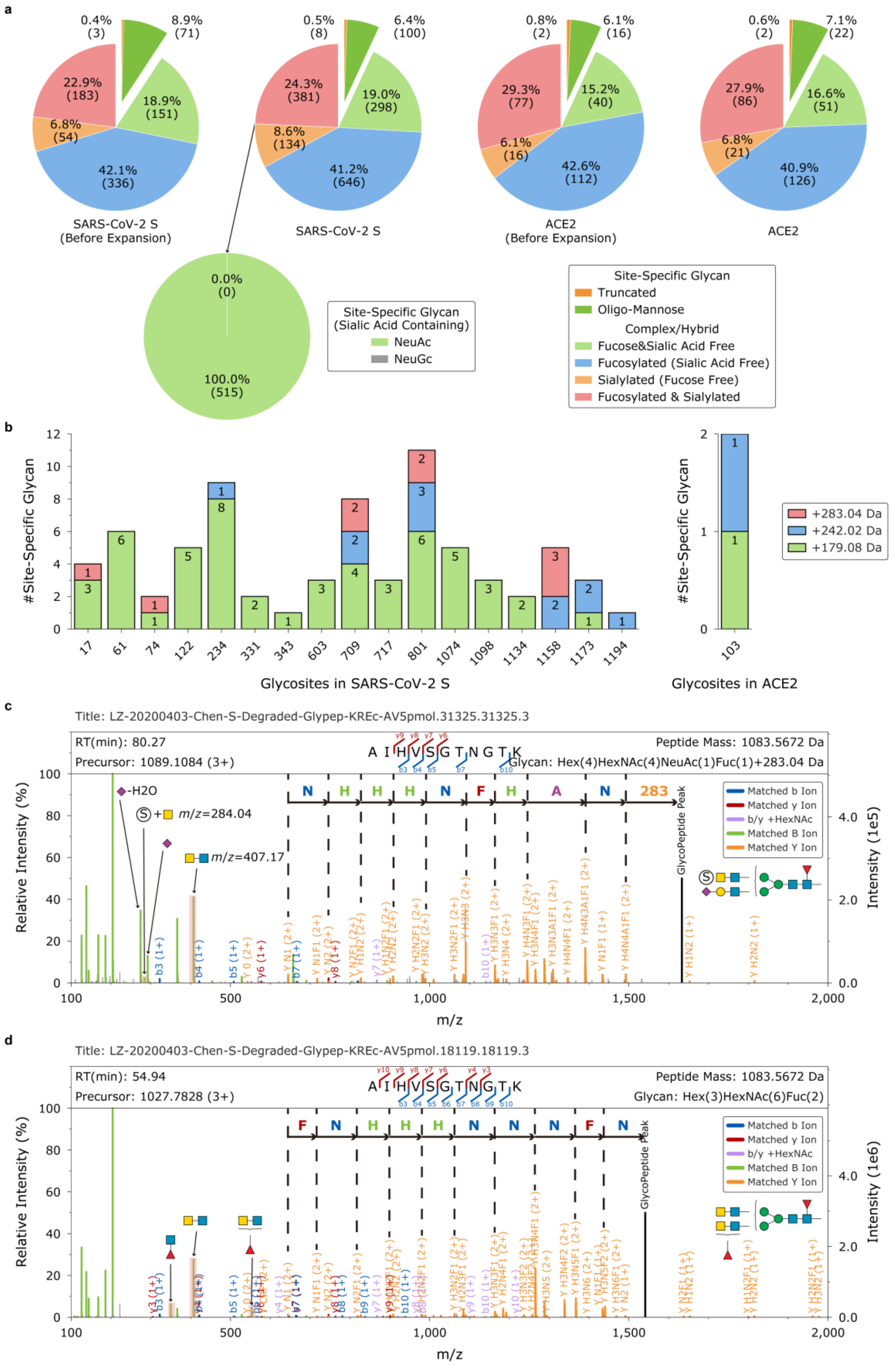

Supplementary Figure 33 Distributions of the number of site-specific glycans in

different categories provided by Glyco-Decipher on the datasets of SARS-CoV-2 Spike and ACE2.

(a) Distributions of the number of site-specific glycans in different categories on the datasets of SARS-CoV-2 S (left) and ACE2 (right). It is worth noting that no site-specific glycans with NeuGc were detected in the results of Glyco-Decipher, which is in line with the glycosylation rule of SARS-CoV-2 Spike protein cultured in HEK293 T cell. This result also indicates the high confidence of Glyco-Decipher in glycan assignment and glycopeptide identification.

(b) The distribution of modified glycans, including glycans linked with moieties of 179 Da (Hex + 17 Da), 242 Da (Hex + 80 Da) and 283 Da (HexNAc + 80 Da), on the sites of SARS-CoV-2 S (left) and ACE2 (right).

(c) Spectrum example of the glycopeptide with the peptide backbone of "AIHVSGTNGTK" for glycosite N74 in SARS-CoV-2 spike and the glycan part of Hex(4)HexNAc(4)NeuAc(1)Fuc(1) +283 Da modification moiety identified in Glyco-Decipher. The glycan with sulfated LacDiNAc terminal was identified by the monosaccharide stepping method. The diagnostic ions of sulfated HexNAc (*m/z* = 284.04) and LacDiNAc (*m/z* = 407.17) were also observed in the glycopeptide spectrum.

(d) Spectrum example of the glycopeptide with the peptide backbone of "AIHVSGTNGTK" for glycosite N74 in SARS-CoV-2 spike and the glycan part of Hex(3)HexNAc(6)Fuc(2) identified in Glyco-Decipher. This glycan with fucosylated LacDiNAc terminal was identified by glycan database searching in Glyco-Decipher.

The diagnostic ions of fucosylated HexNAc (*m/z* = 284.04) and LacDiNAc (*m/z* = 407.17) were also observed in the glycopeptide spectrum.

Glycans with terminal LacDiNAc are significantly produced by HEK293 T cell[6, 7], which was used to express the SAR-CoV-2 Spike protein in the original study[5]. For example, based on prior studies of glycosylation on SARS-CoV-2 spike[5, 7], N74 is the site carrying the most amount of sulfation and additional fucosylation due to the presence of LacDiNAc. The detection of oxonium ion at *m/z* 407.17 in the glycopeptide spectra indicated the presence of LacDiNAc terminal on the glycans identified by Glyco-Decipher. In addition, a modification moiety of 283.04 Da, which matches to the mass of sulfated HexNAc, was identified by monosaccharide stepping method (as presented in (c)). The diagnostic ion of HexNAc(1)S(1) at *m/z* 284.04 in the glycopeptide spectrum supports the sulfate modification identification on the glycan. Similarly, ions of HexNAc(1)Fuc(1) at *m/z* 350.15 and HexNAc(2)Fuc(1) at *m/z* 553.23 also supported the presence of fucosylated LacDiNAc terminal (as presented in (d)), indicating the capability of Glyco-Decipher in distinguishing terminal structures of glycans.

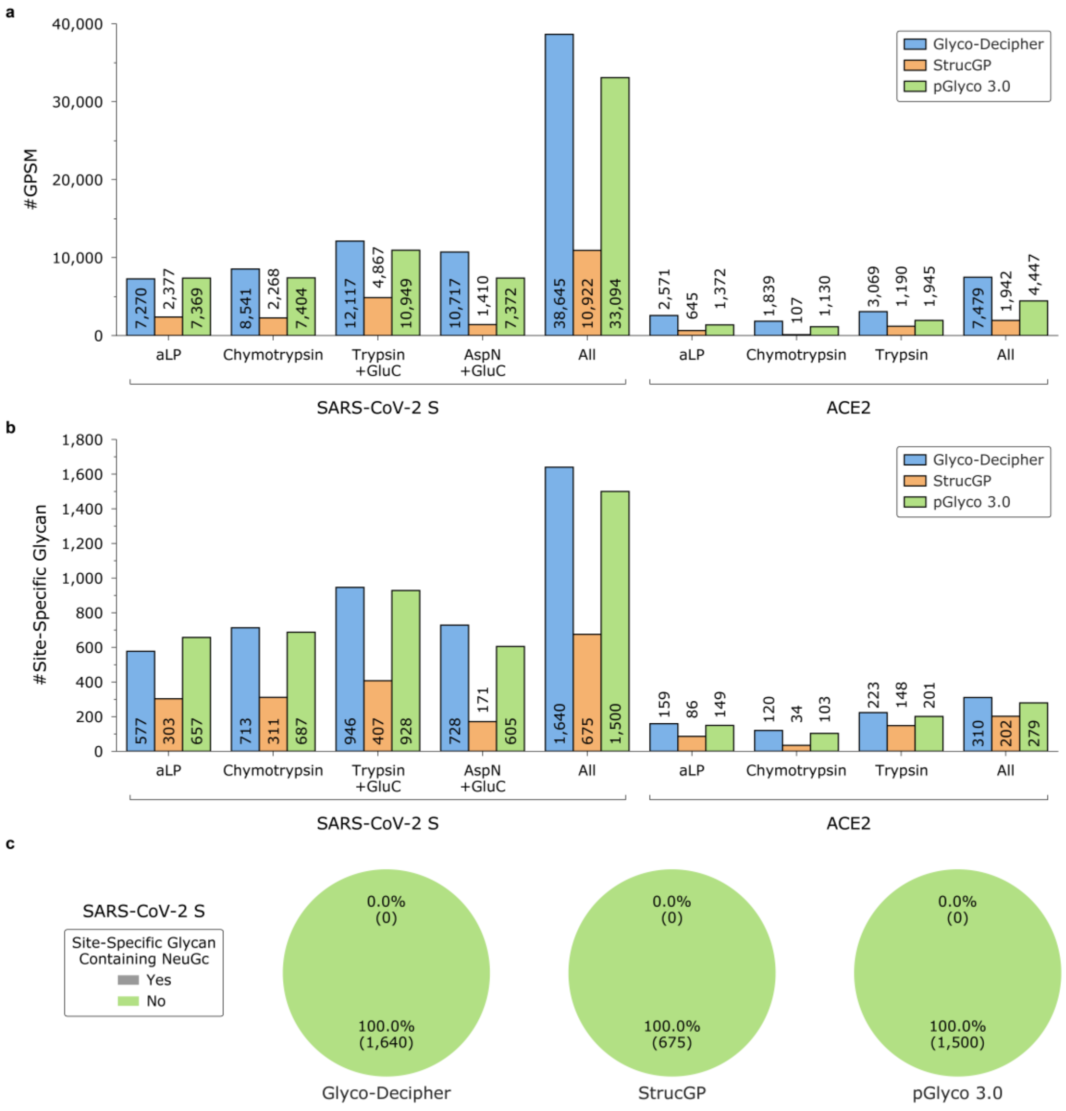

Supplementary Figure 34 Comparison of the identification results of Glyco-Decipher and StrucGP/pGlyco 3.0 on the datasets of SARS-CoV-2 Spike and ACE2.

(a) Comparison of the identified glycopeptide spectra by Glyco-Decipher and StrucGP/pGlyco 3.0.

(b) Comparison of the site-specific glycan results provided by Glyco-Decipher and StrucGP/pGlyco 3.0.

(c) The distributions of site-specific glycans containing NeuGc in the results of Glyco-

Decipher, StrucGP and pGlyco 3.0.

In the performance comparison between Glyco-Decipher and StrucGP/pGlyco 3.0, the glycan database of GlyTouCan was used in Glyco-Decipher and pGlyco 3.0 and the built-in database of N-Glycan core/branch structures was adopted in StrucGP. In comparison the results of StrucGP, Glyco-Decipher provided 143.0% and 53.5% more site-specific glycans on SARS-CoV-2 S and ACE2, respectively. In addition, 95.7% (SARS-CoV-2 S) and 89.1% (ACE2) of the results of StrucGP were covered by Glyco-Decipher. In comparison with pGlyco 3.0, +80 Da on Hex and +80 Da on HexNAc were added into the monosaccharide modification list of pGlyco 3.0 to search modified glycans. In total, 74.8% (SARS-CoV-2 S) and 79.2% (ACE2) of the results of pGlyco 3.0 were covered by Glyco-Decipher. And the increases of 9.3% and 11.1% in the number of total identified site-specific glycans were provided by Glyco-Decipher on the data of SARS-CoV-2 S and ACE2 compared to pGlyco 3.0. Specifically, 3 site-specific glycans with (Hex +80 Da) and 1 site-specific glycan with (HexNAc + 80 Da) were identified on SARS-CoV2 spike protein by pGlyco 3.0, including one sulfated site-specific glycan (Hex(3)HexNAc(6)Fuc(1)S(1) at N1173) with diagnostic ions in MS2. In contrast, 11 site-specific glycans with (Hex +80 Da) and 9 site-specific glycans with (HexNAc +80 Da) on SARS-CoV-2 spike protein were unveiled by Glyco-Decipher, including Hex(4)HexNAc(5)NeuAc(1)Fuc(1)S(1) at N74, which was reported to account for high occupancy at the glycosite[5, 7].

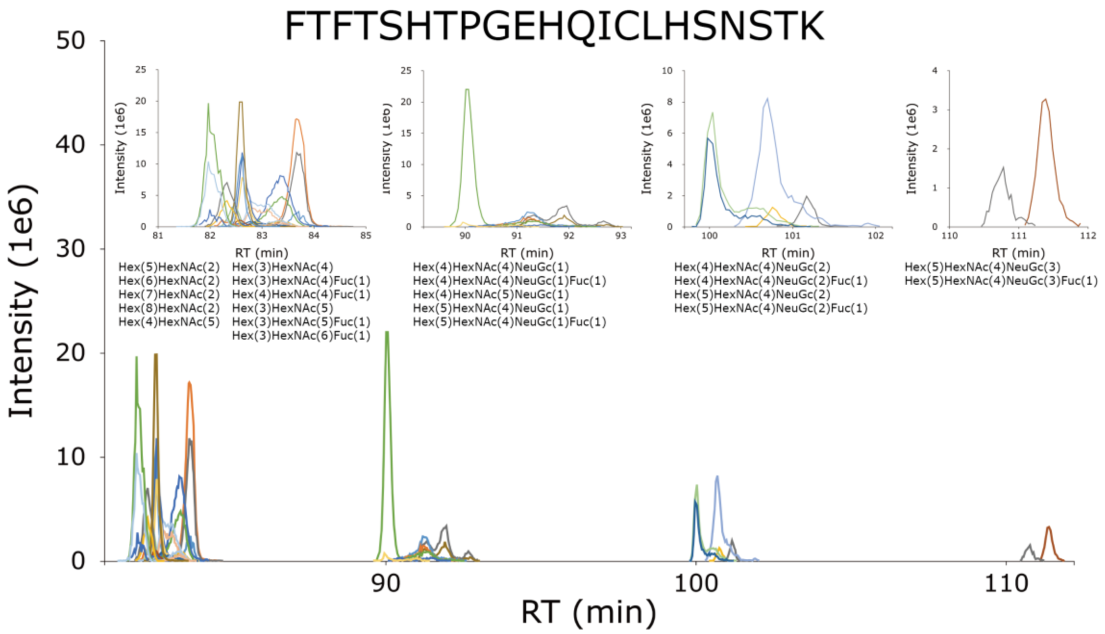

Supplementary Figure 35 Example of elution profiles of glycopeptides with the same peptide backbone.

Elution profiles of 22 unique glycopeptides with the same peptide sequence "FTFTSHTPGEHQICLHSNSTK" but with different glycans were extracted by Glyco-Decipher. Detailed elution profiles of glycopeptides with 0-3 sialic acids were shown in the top. Source data are provided as a Source Data file.

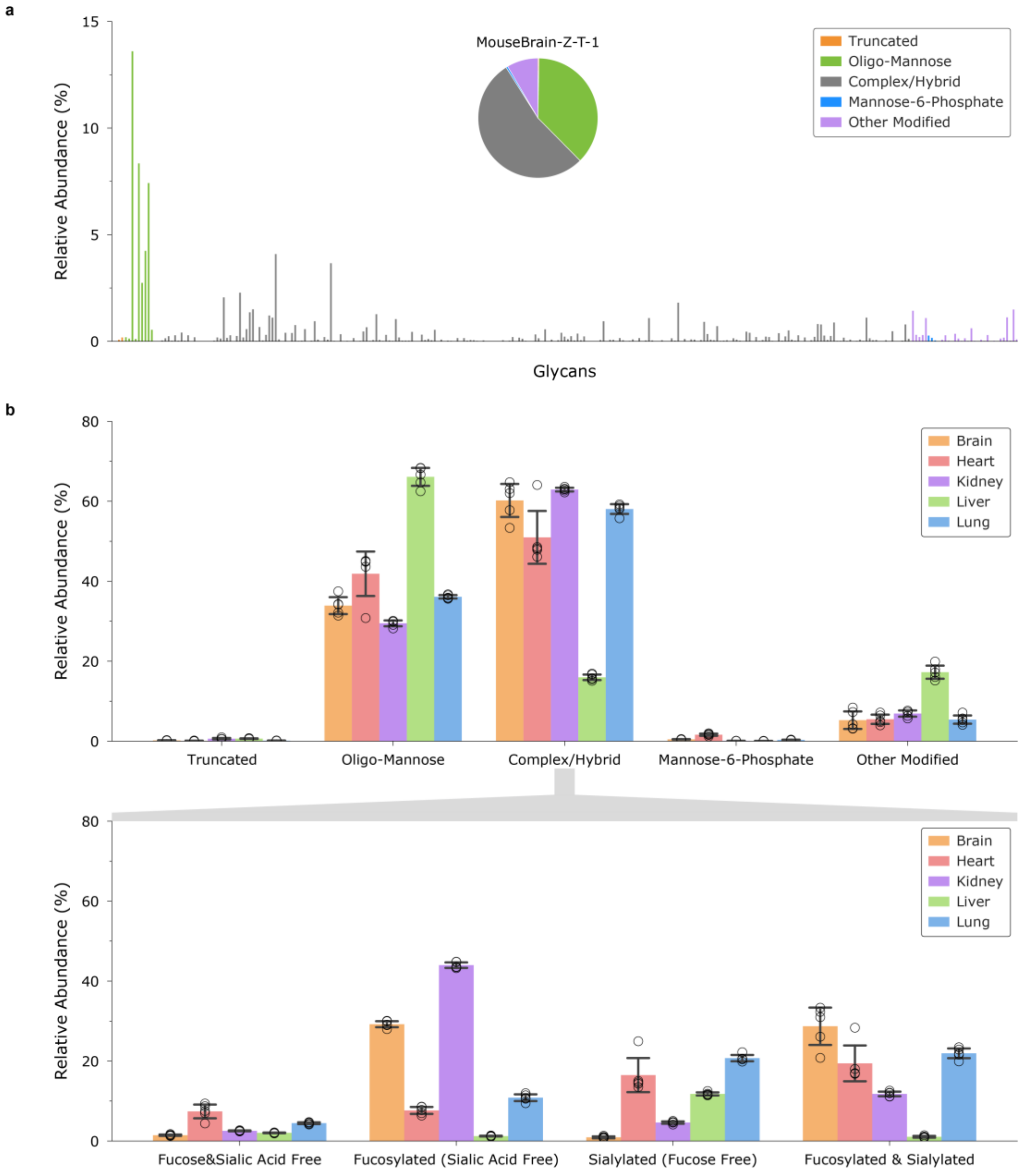

Supplementary Figure 36 Investigation of glycan abundance at sample level.

(a) Abundance distribution of each glycan identified in the file “MouseBrain-Z-T-1”.

(b) Relative abundance of different types of glycans in five mouse tissues. The specific values (dot), average values (bar) and standard deviations (error bar line) of five technical replicates for each tissue are indicated. Source data are provided as a Source Data file.

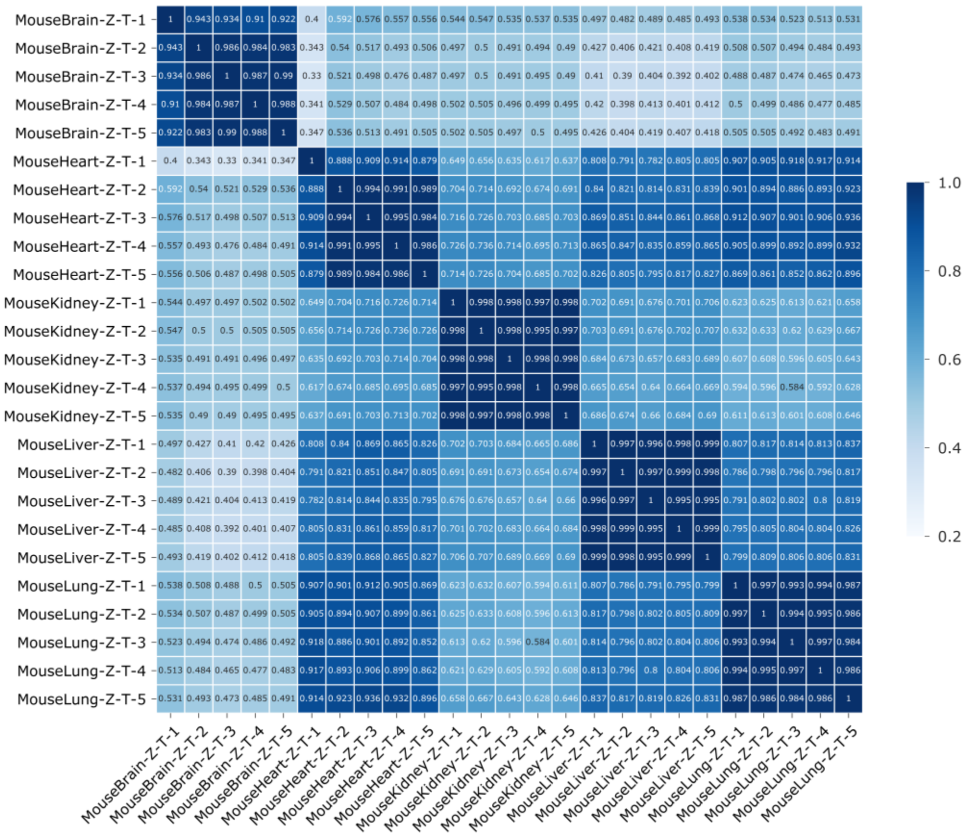

Supplementary Figure 37 Pearson correlation of glycan abundance between each LC-MS/MS run of five tissues.

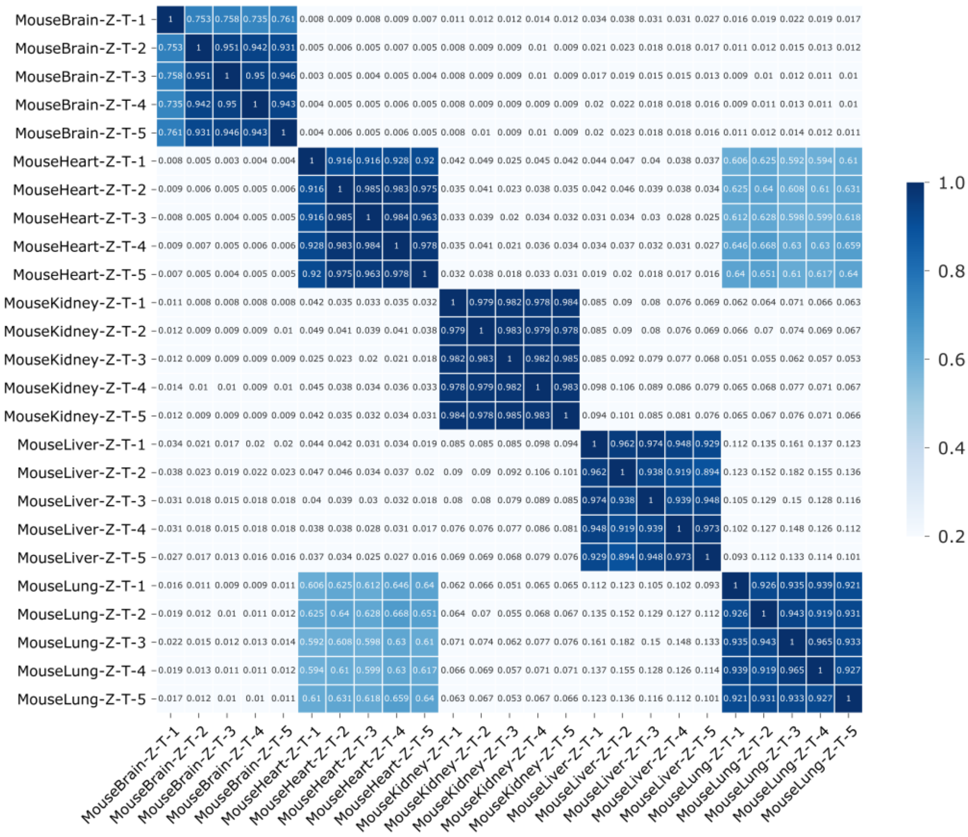

Supplementary Figure 38 Pearson correlation of abundance of site-specific glycosylation in five mouse tissues.

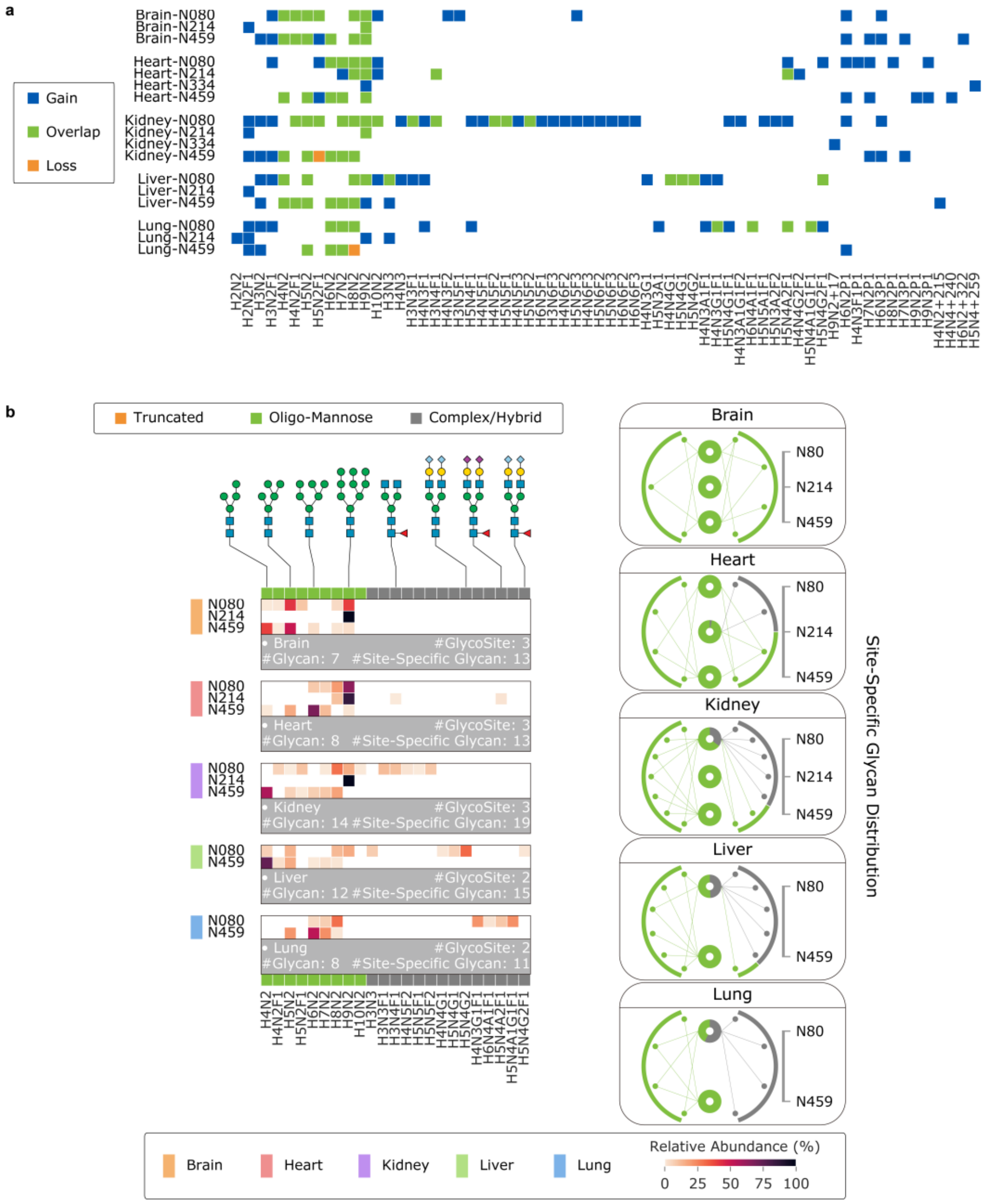

Supplementary Figure 39 Investigation of identification results and abundance distributions of site-specific glycans on prosaposin in mouse tissues provided by StrucGP.

(a) Comparison of site-specific glycans identified by Glyco-Decipher and StrucGP on prosaposin in five mouse tissues. Additional, overlap and lost identifications of Glyco-Decipher compared to StrucGP are indicated by blue, green and orange boxes,

respectively.

(b) Relative abundance distribution of glycans at each glycosite in prosaposin across five mouse tissues revealed by StrucGP (heat map). Compositions of the high-abundance glycans are annotated at the top. For a more intuitive demonstration, the abundance distributions of glycans at each glycosite are listed in the right radial diagrams. In each radial diagram, nodes around the circle denote glycans linked to prosaposin, and donuts in the center denote glycosites identified in prosaposin. Linkage between the node and the center donut indicates that the glycosite was modified by the corresponding glycan. The percentage value in each donut indicates the relative abundance for a certain type of glycan.

Monosaccharide abbreviation: H: Hex; N: HexNAc; A: NeuAc; G: NeuGc; F: Fuc.

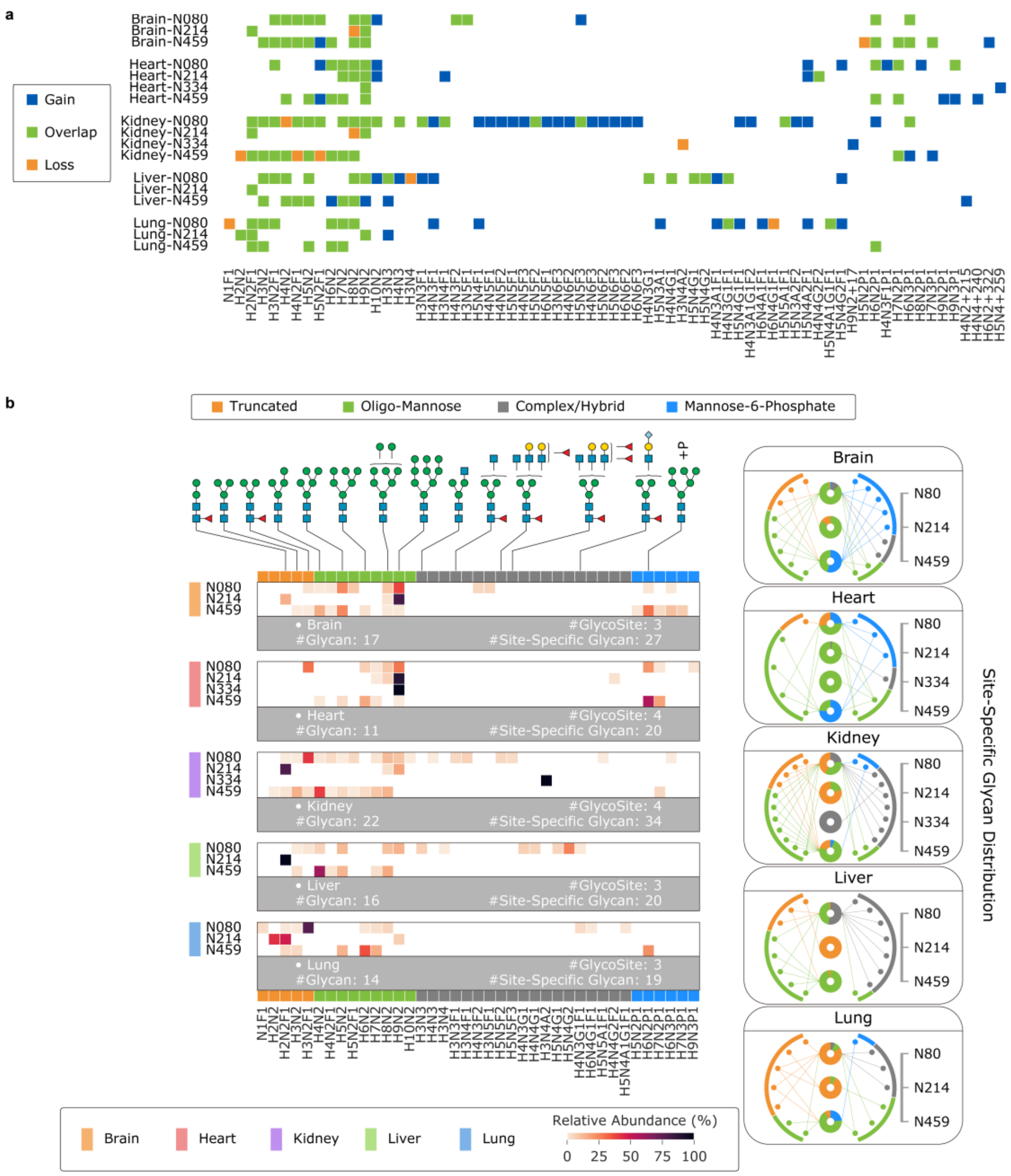

Supplementary Figure 40 Investigation of identification results and abundance distributions of site-specific glycans on prosaposin in mouse tissues provided by pGlyco 3.0.

(a) Comparison of site-specific glycans identified by Glyco-Decipher and pGlyco 3.0 on prosaposin in five mouse tissues. Additional, overlap and lost identifications of Glyco-Decipher compared to pGlyco 3.0 are indicated by blue, green and orange boxes,

respectively.

(b) Relative abundance distribution of glycans at each glycosite in prosaposin across five mouse tissues revealed by pGlyco 3.0 (heat map). Compositions of the high-abundance glycans are annotated at the top. For a more intuitive demonstration, the abundance distributions of glycans at each glycosite are listed in the right radial diagrams. In each radial diagram, nodes around the circle denote glycans linked to prosaposin, and donuts in the center denote glycosites identified in prosaposin. Linkage between the node and the center donut indicates that the glycosite was modified by the corresponding glycan. The percentage value in each donut indicates the relative abundance for a certain type of glycan.

Abbreviation: H: Hex; N: HexNAc; A: NeuAc; G: NeuGc; F: Fuc; P: Phosphorylation.

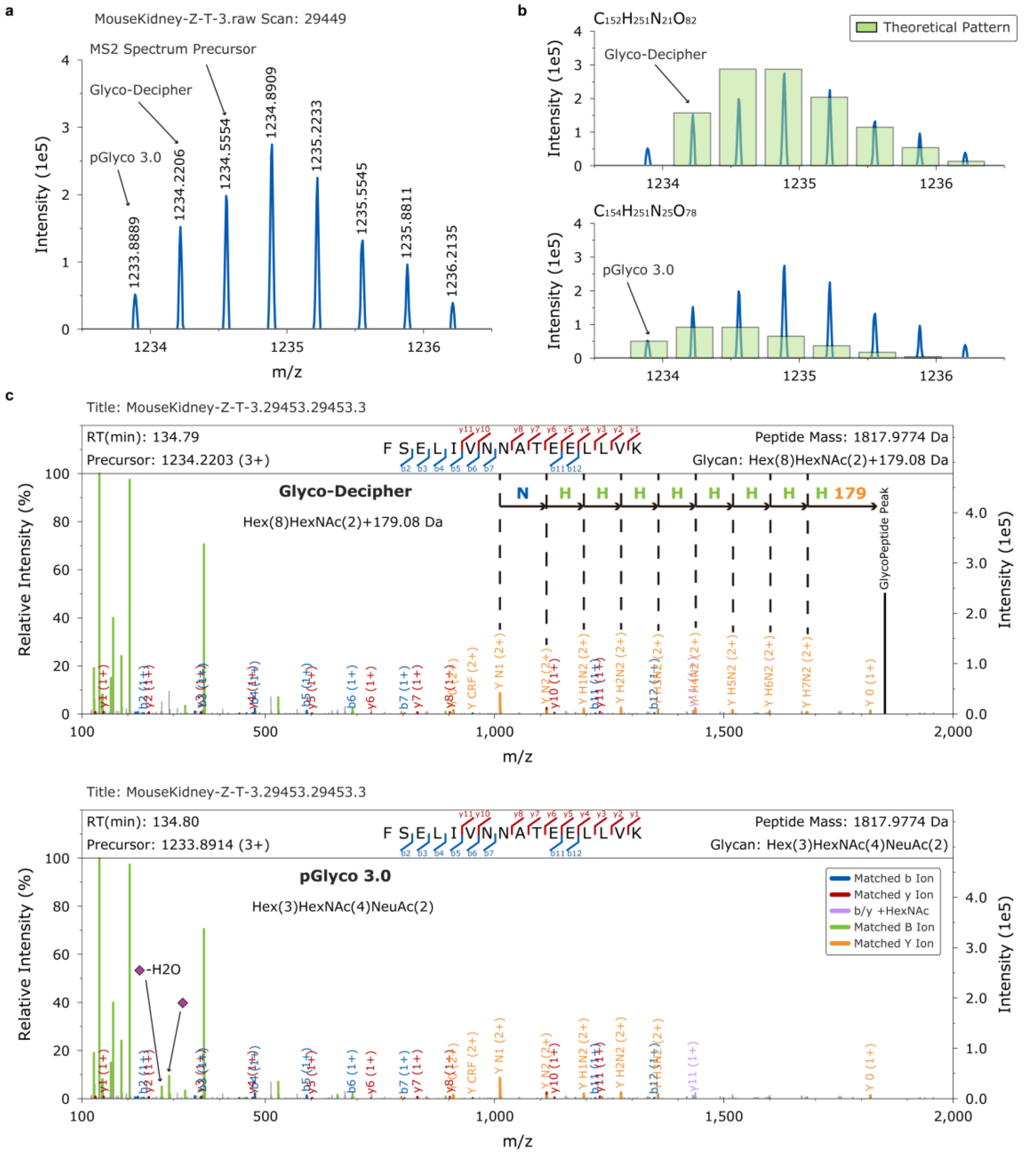

Supplementary Figure 41 Analysis of the chimeric spectrum that was matched to distinct glycans at N334 of prosaposin in mouse kidney.

(a) Experimental isotopic cluster of precursor of the glycopeptide spectrum "MouseKidney-Z-T-3.29453.29453.3". The *m/z* value of the original spectrum precursor and corrected precursor (Glyco-Decipher/pGlyco 3.0) are labeled in the figure. Source data are provided as a Source Data file.

(b) Comparison of the theoretical isotopic pattern derived from the elemental

composition of identified glycopeptides with the experimental cluster.

(c) Annotation of the glycopeptide spectra “MouseKidney-Z-T-3.29453.29453.3” based on the identification results of Glyco-Decipher (top) and pGlyco 3.0 (bottom). Only one glycopeptide spectra corresponding to the glycosite N334 of prosaposin in kidney was identified. The difference of identification in Glyco-Decipher and pGlyco 3.0 is at the glycan part: The glycan of Hex(8)HexNAc(2) +179 Da was identified in Glyco-Decipher and continuous Y ions with the gap of Hex were matched in MS2. The glycan of Hex(3)HexNAc(4)NeuAc(2) was identified in pGlyco 3.0 and the diagnostic oxonium ions of NeuAc were also matched in the glycopeptide spectrum. The mass difference of the two glycans is 1 Da which is corresponding to the difference of the corrected precursors of the two software. The precursor of Glyco-Decipher is the +1 value of the precursor of pGlyco 3.0 and accounted for higher isotopic similarity and ion intensity in the precursor isolation window than that of pGlyco 3.0 (as shown in (b)).

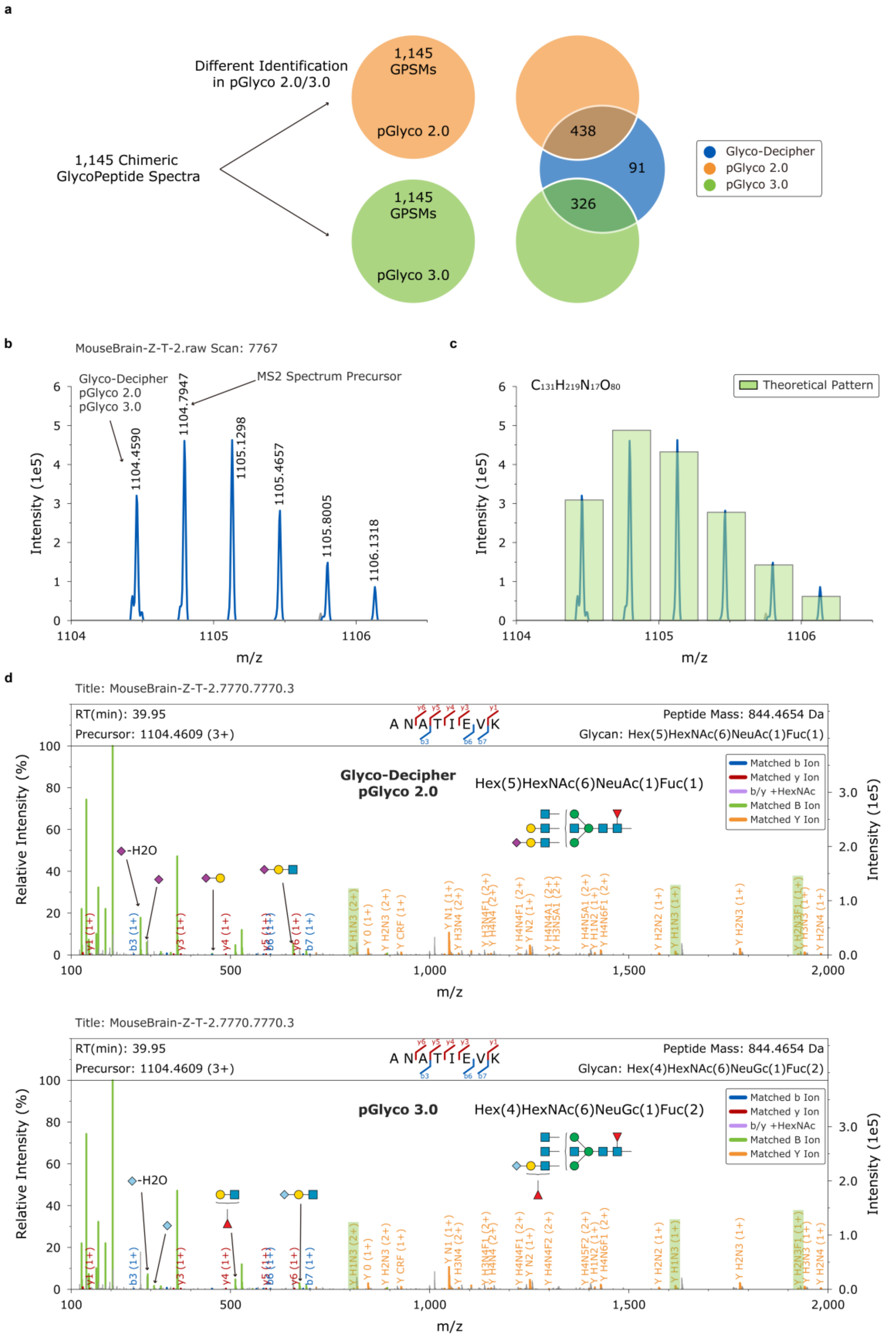

Supplementary Figure 42 Analysis of identification results of Glyco-Decipher and pGlyco 2.0/3.0 on chimeric glycopeptide spectra in the mouse dataset.

(a) Analysis of the identification results of 1,145 chimeric glycopeptide spectra that were matched to distinct glycopeptides in pGlyco 2.0 and pGlyco 3.0. Among the 1,145 glycopeptide spectra, 855 of them were also identified in Glyco-Decipher and the results of Glyco-Decipher were consistent to pGlyco results in 89.4% (764/855) of these spectra.

(b) Experimental isotopic cluster of the precursor of glycopeptide spectrum "MouseBrain-Z-T-2.7770.7770.3". The *m/z* values of original spectrum precursor and corrected precursors (Glyco-Decipher, pGlyco 2.0/3.0) were labeled in the figure. Source data are provided as a Source Data file.

(c) Comparison of the theoretical isotopic pattern with the experimental cluster. The theoretical isotopic pattern was derived from the composition of identified glycopeptide.

(d) Annotation of the glycopeptide spectra "MouseBrain-Z-T-2.7770.7770.3" based on the identification results of Glyco-Decipher/pGlyco 2.0 (top) and pGlyco 3.0 (bottom). Diagnostic oxonium ion and Y ions for glycan identification and structure discrimination (core fucosylation and bisected HexNAc) were labeled in the figure. From this glycopeptide spectrum, peptide backbone of "ANATIEVK" was matched in all software. Hex(5)HexNAc(6)NeuAc(1)Fuc(1) were identified as the glycan part in both Glyco-Decipher and pGlyco 2.0 and Hex(4)HexNAc(6)NeuGc(1)Fuc(2) was reported in pGlyco 3.0. Although identical mass values are shared by the two glycans, distinct diagnostic oxonium ions were generated by them and were both detected in this chimeric glycopeptide spectra.

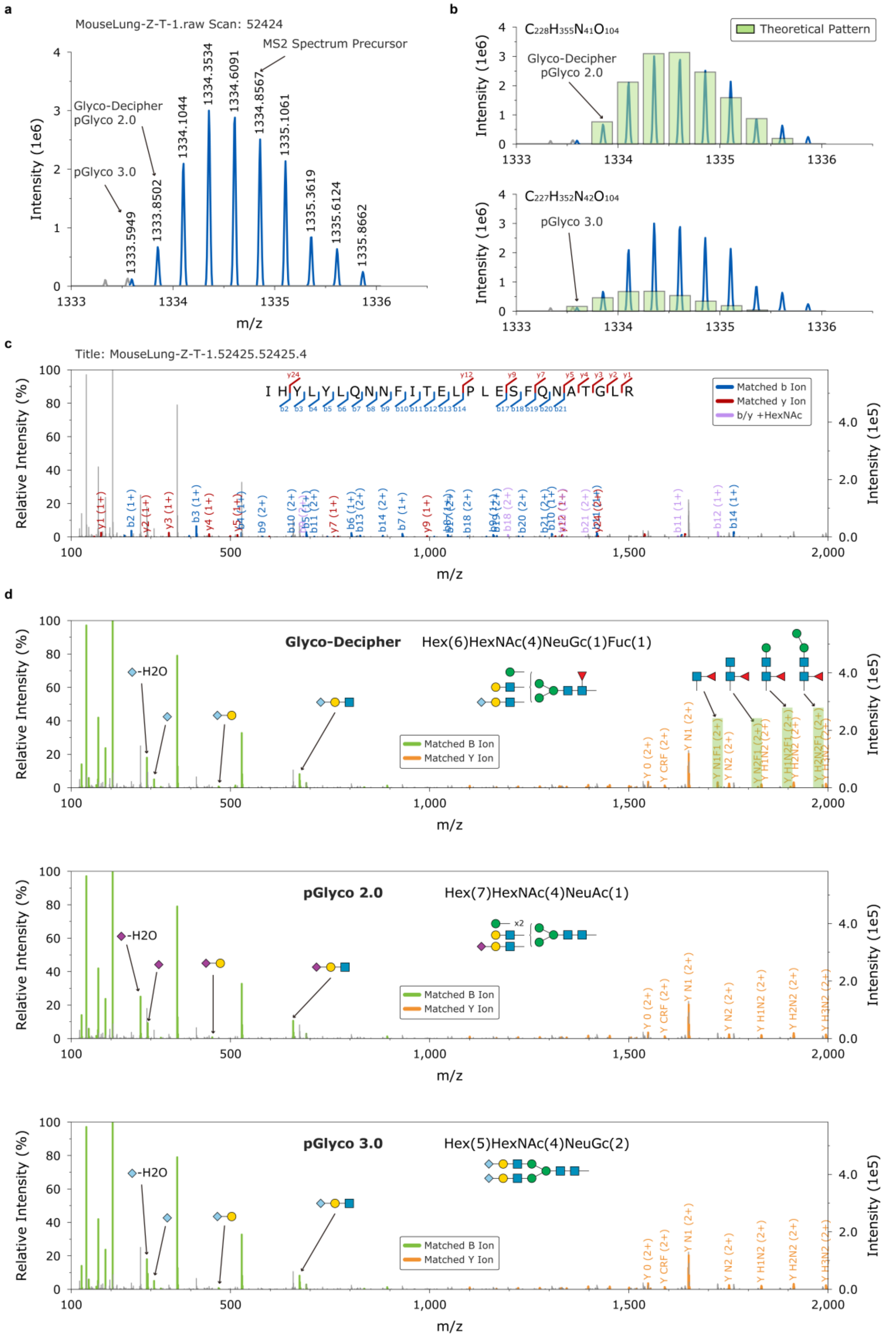

Supplementary Figure 43 Analysis of the chimeric spectrum that was identified to distinct glycopeptides in Glyco-Decipher and pGlyco 2.0/3.0.

(a) Experimental isotopic cluster of the precursor of glycopeptide spectrum "MouseLung-Z-T-1.52425.52425.4". The *m/z* values of original spectrum precursor and corrected precursor (Glyco-Decipher, pGlyco 2.0/3.0) were labeled in the figure. Source data are provided as a Source Data file.

(b) Comparison of the theoretical isotopic pattern with the experimental cluster. The theoretical isotopic pattern was derived from the composition of identified glycopeptide.

(c) Annotation of the glycopeptide spectrum "MouseLung-Z-T-1.52425.52425.4" with the peptide backbone "IHYLYLQNNFITELPLESFQNATGLR" which was commonly identified in Glyco-Decipher and pGlyco 2.0/3.0.

(d) Annotation of the glycopeptide spectra "MouseLung-Z-T-1.52425.52425.4" based on the glycan identification results of Glyco-Decipher (top), pGlyco 2.0 (middle) and pGlyco 3.0 (bottom).

In this glycopeptide spectrum, identical peptide backbone was matched in all the three software. Sufficient peptide fragment ions were matched in the spectrum to support high confidence identification of the peptide backbone, yet only core structure glycan ions were matched for the identification of glycan part in the three software tools. The identification differences between the three tools are at the precursor detection and glycan assignment: In terms of precursor detection, the isotopic pattern and ion intensity in the isolation window are key factors of precursor correction in Glyco-Decipher. In addition, Glyco-Decipher also scores glycan candidates of different precursors by matching the corresponding glycan ions in MS2, and the glycan scoring also helps to

determine the precursor. As a result, the precursor with higher pattern similarity/ion intensity and the glycan (Hex(6)HexNAc(4)NeuGc(1)Fuc(1)) with more matched glycan ions were identified in Glyco-Decipher (top). The diagnostic oxonium ions of NeuGc and Y ions of core fucosylation were observed in the spectrum.

Different precursors and glycans were identified in pGlyco 2.0/3.0: Although the identical precursor for this spectrum was detected in pGlyco 2.0, another NeuAc-containing glycan (Hex(7)HexNAc(4)NeuAc(1)) was matched in pGlyco 2.0. Since the tuned algorithm in precursor detection, a different precursor was detected in pGlyco 3.0. As described in the original publication[8], pGlyco 3.0 tend to correct the precursor to the one with less *m/z* value: "*For potential chimeric spectra, pGlyco3 removes unreliable mixed glycopeptides by determining whether one's precursor is another's isotope. For example, if NeuAc(1) and Fuc(2) are simultaneously identified in the same MS2 scan but with different precursors, the Fuc(2)-glycopeptide will be removed because 'NeuAc(1) + 1 Da = Fuc(2)*". And the preference was also observed in this spectrum example: Hex(5)HexNAc(4)NeuGc(2) was identified in pGlyco 3.0 and it corresponds to the precursor with lowest *m/z* value but less isotopic similarity and ion intensity in the isolation window (as shown in (b)). In this chimeric spectrum example, distinct glycans were matched in Glyco-Decipher and pGlyco 2.0/3.0. And precursor and fragment ion evidences are found to support all these glycan identifications. Thus improvements for precursor detection and glycan assignment are needed for the glycoproteomics tools for more comprehensive identification of glycopeptides.

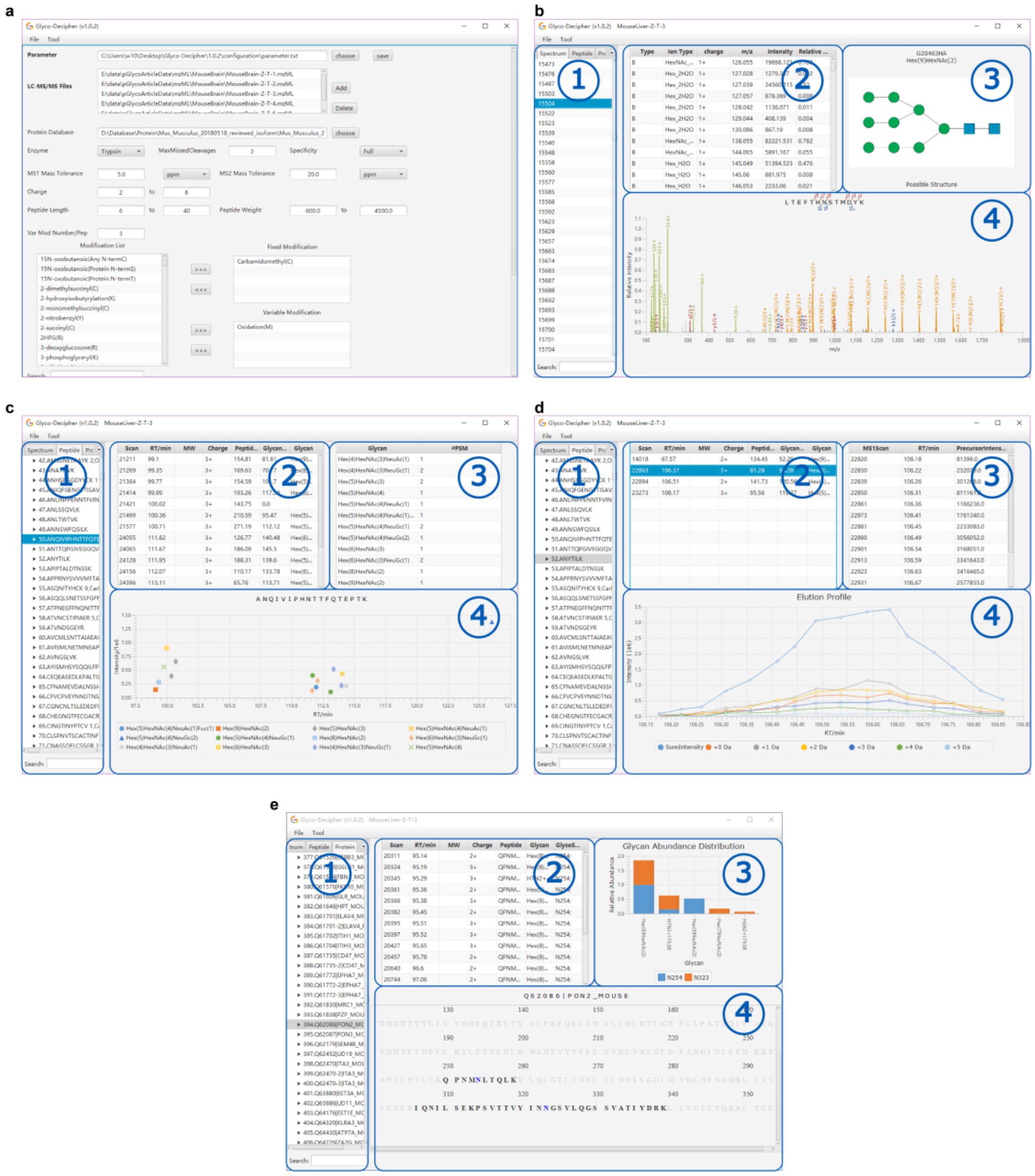

Supplementary Figure 44 Graphical User Interface of Glyco-Decipher.

(a) Intact glycopeptide search task pane.

(b) MS2 Spectrum exhibition pane: 1). List of all identified MS2 spectrum in intact glycopeptide data; 2). Information of matched b/y, B/Y fragment ions; 3). Glycan composition and possible structure provided by database; 4). MS2 spectrum with labeled fragment ion information.

(c) Peptide exhibition pane: 1) List of all identified peptide in LC-MS/MS data; 2)

GPSM list corresponding to peptide item selected in (1); 3) Spectrum number distribution of each glycopeptide; 4) Retention time distribution of each MS2 spectrum in which the selected peptide was identified (x axis: retention time, y axis: intensity of precursor)

(d) Glycopeptide quantification pane: 1) List of all identified peptide in LC-MS/MS data; 2) GPSM list corresponding to peptide item selected in (1); 3) Isotopic peak intensity extracted in each MS1 spectrum; 4) Elution profile of each isotopic peak and intensity summary.

(e) Protein exhibition pane: 1) List of all identified glycoprotein in LC-MS/MS data; 2) GPSM list corresponding to protein item selected in (1); 3) Distribution of glycan abundance at each site; 4) Protein sequence and glycosite information.

Supplementary Note 1

In silico deglycosylation enables sensitive identification of peptide backbones of intact glycopeptides.

A dataset containing 25 raw files acquired by LC-MS/MS (Liquid chromatography-tandem mass spectrometry) analysis of intact glycopeptides from five mouse tissues (brain, heart, kidney, liver and lung) digest[2] was used to evaluate the performance of peptide backbone identification of Glyco-Decipher. Among 1,386,844 acquired MS2 spectra, 1,282,263 spectra were determined to be glycopeptide spectra contained more than 2 oxonium ions. These spectra were subjected to penta-saccharide core structure matching, and 556,363 spectra were matched with at least 3 of the core structure peaks in MS2. We first investigated the number of generated spectra when different numbers of matched core structures considered in the generation of in silico deglycosylation spectrum (Supplementary Fig. 45). It was found that near 2 times of deglycosylated spectra were generated when considering up to top 5 candidate core structures. In terms of peptide backbone identification, majority results (>90%) were obtained from the deglycosylated spectra corresponding to the top-scored core structure with highest Y1 (Y-HexNAc) intensity (Supplementary Fig. 45b). As other core structures yielded few peptide identifications, only the spectra generated by the top scored core structure with highest Y1 intensity was retained for peptide backbone identification in Glyco-Decipher if not otherwise stated.

MAGIC[9], which also adopts the in silico deglycosylation strategy, was employed for

comparison. From the 1,282,263 oxonium ion-containing glycopeptide spectra, similar number of spectra (Glyco-Decipher: 556,363, MAGIC: 509,117) were matched for subsequent deglycosylation in this two software (Supplementary Fig. 46a). And from peptide identification of deglycosylated results, 55.3% more peptide-spectrum matches (PSMs) (Glyco-Decipher: 132,534, MAGIC: 85,319) were obtained by Glyco-Decipher compared to MAGIC (1% PSM FDR, Supplementary Fig. 46b). To account for this improvement, we then compared the identification results for the 409,835 overlapping glycopeptide spectra from which both software matched core structure peaks. It was found that 113,756 PSMs were identified in Glyco-Decipher while only 82,834 PSMs were obtained in MAGIC (Supplementary Fig. 46c), indicating better performance of Glyco-Decipher in peptide mass deviation and glycopeak removal.

Supplementary Fig. 46d presents an example to illustrate the different performance of glycopeak removal between Glyco-Decipher and MAGIC. Compared to the original intact glycopeptide spectrum illustrated in Fig. 2a, B/Y fragment ions were efficiently removed by Glyco-Decipher, while Y fragment ions at high charge state (3+) and fragments with water loss were not fully removed in MAGIC, which resulted in a PSM with different confidence levels. The inspection of percentage of peptide fragments in in silico deglycosylation results also suggests better glycopeak removal performance of Glyco-Decipher (Supplementary Fig. 47). In addition to normal b/y ions, peptide fragments linked with an HexNAc residue were also commonly observed in higher-energy collisional dissociation (HCD) spectra of glycopeptide (Supplementary Fig. 48)

and provided more fragments information in peptide identification (Supplementary Fig. 46c). Benefit from the accurate peptide mass determination and the efficient glycopeak removal, Glyco-Decipher enables sensitive identification of peptides from glycopeptide spectra without using of glycan databases.

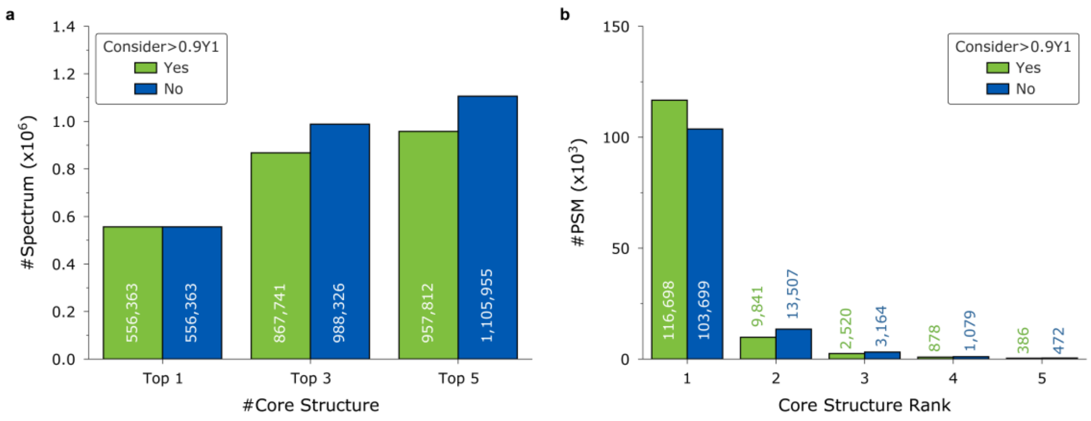

Supplementary Figure 45 The numbers of in silico deglycosylated spectrum and the numbers of PSMs yielded when different number of matched core structures were considered.

(a) Numbers of in silico deglycosylated spectra generated when top 1/3/5 matched core structure(s) were considered in spectrum generation.

(b) Rank distribution of matched core structure in identification results from in silico deglycosylated spectra. Effect of Y1 intensity was also considered in spectrum generation: consider >0.9Y1 means only one core structure retained when over 0.9 relative intensity Y1 peak matched in core structure results, the retained value corresponding to the one with highest Y1 peak intensity.

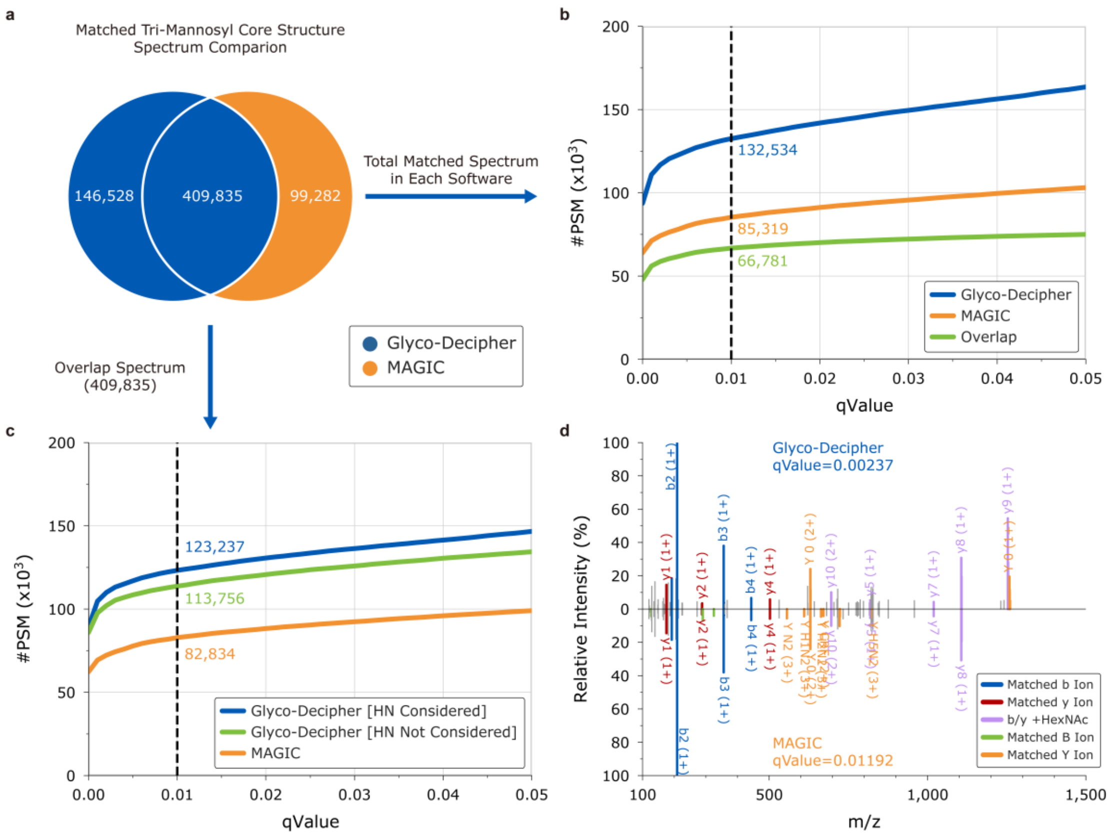

Supplementary Figure 46 Higher sensitivity of Glyco-Decipher in peptide identification compared with MAGIC.

In this comparison, MS2 mass tolerance was set to 0.02 Da in Glyco-Decipher and MAGIC, because ppm was not supported in MAGIC. And 20 ppm in MS2 was used across the whole workflow in main text. Source data are provided as a Source Data file.

(a) Comparison of spectra matched with core structure peaks by Glyco-Decipher and MAGIC. In the overlapping part, spectra matched core structure peaks in both Glyco-Decipher and MAGIC.

(b) PSM identification results achieved by Glyco-Decipher and MAGIC at different confidence levels. The dashed line indicates the identification results with qValue <0.01.

(c) Numbers of PSMs achieved by Glyco-Decipher and MAGIC from the spectra with

penta-saccharide core structures matched by both Glyco-Decipher and MAGIC.

[HN considered] means the peaks of b/y ions linked with HexNAc moiety are also took into consideration in peptide identification in Glyco-Decipher. The dashed line indicates the identification results with qValue <0.01.

(d) The peak matching for the in silico deglycosylated spectra generated by Glyco-Decipher (top) and MAGIC (bottom) for the intact glycopeptide spectrum "MouseLiver-Z-T-1.25466.25466.3" in which the peptide backbone "AHFSSLNLTIR" was matched. The smaller qValue indicates higher confidence of the peptide spectrum matching.

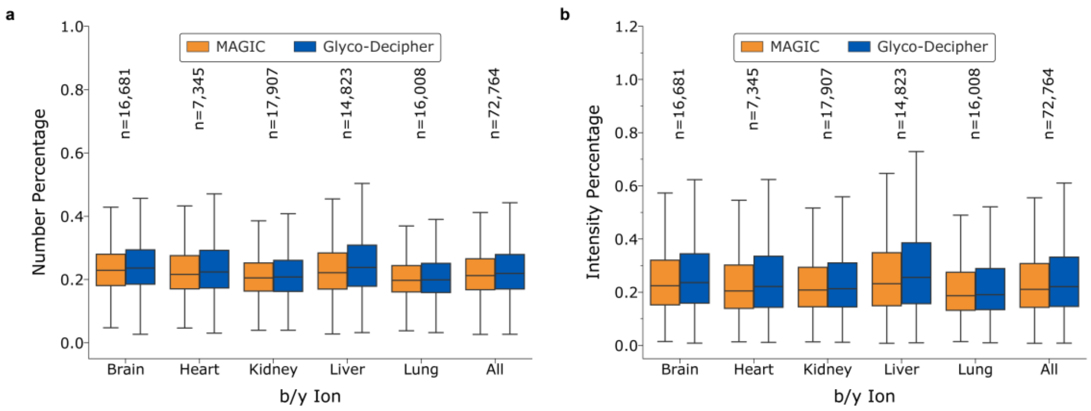

Supplementary Figure 47 Statistics of peptide fragment ions in the in silico degycosylated spectra.

The percentage of (a) number and (b) intensity of matched b/y ions against total signals in generated spectra from MAGIC and Glyco-Decipher were compared, where 72,764 PSMs in which the same peptide matched by both software were used. The boxes show interquartile ranges (IQR), including median (middle line) and $25^{th}/75^{th}$ percentile (box), and whiskers indicate 1.5 × IQR values; no outliers are shown and the spectrum numbers are labeled in the plot. Source data are provided as a Source Data file.

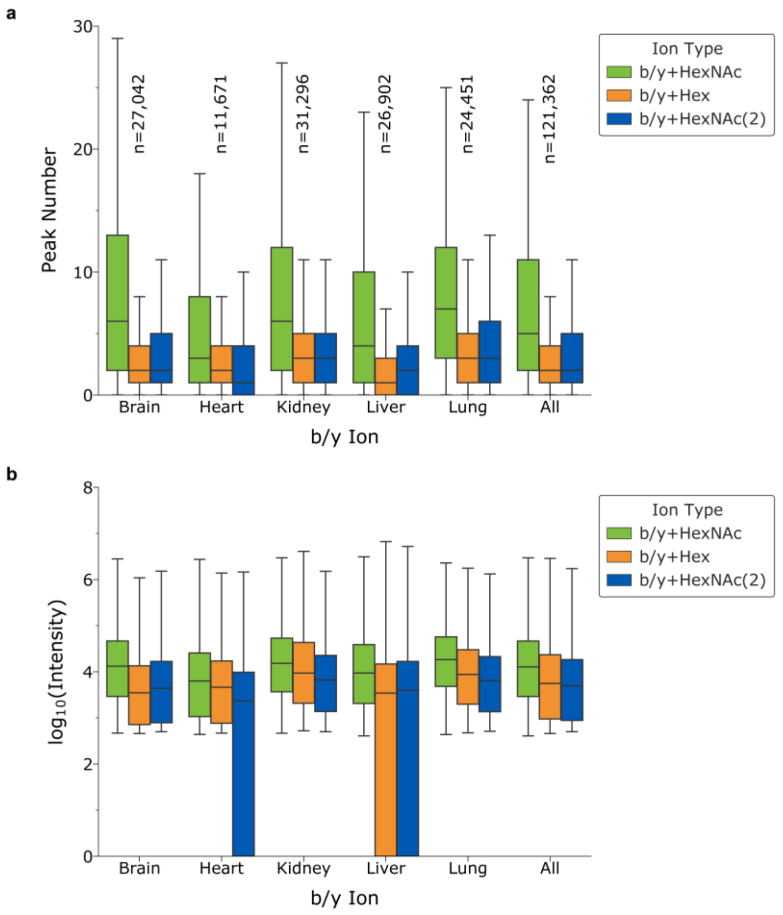

Supplementary Figure 48 Statistics of b/y +HexNAc ions in glycopeptide spectra.

Comparison of (a) number and (b) intensity of matched b/y+HexNAc ions, b/y+Hex and b/y+2HexNAc in 121,362 PSMs from Glyco-Decipher in silico deglycosylation result. The b/y +Hex ions which are impossible for glycopeptide spectra were matched as random match control. As predicted from the core structure of N-glycan, b/y ions linked with two HexNAc could theoretically be generated and were also investigated. The result indicated that they were barely observed in the spectra. The boxes show interquartile ranges (IQR), including median (middle line) and $25^{th}/75^{th}$ percentile (box), and whiskers indicate 1.5 × IQR values; no outliers are shown and the spectrum numbers are labeled in the plot. Source data are provided as a Source Data file.

Supplementary Note 2

Micro-heterogeneity of glycosylation in mouse tissues revealed by Glyco-Decipher.

The datasets of five mouse tissues reported in Liu et al[2] was adopted to investigate the identification performance of Glyco-Decipher. From 1,282,263 glycopeptide MS2 spectra, 215,010 (spectrum identification rate=16.8%) intact glycopeptide-spectrum matches (GPSMs) were identified result in 3,773 unique peptide sequences. From glycan database-independent spectrum identification result, 1,922 glycoproteins were identified and about 2/3 of them were also annotated in UniProt as proteins with glycosylation on them (Supplementary Fig. 49a). In terms of glycosite, 3,884 glycosites were identified. 2,320 of them were also annotated in UniProt and most of them were evidenced by sequence analysis (Supplementary Fig. 49b).

In the identified 215,010 glycopeptide spectra, 75.2% (161,690) of these spectra were annotated with definite glycan compositions (database glycans or modified glycans) which resulted in the identification of 22,809 unique intact glycopeptides. Among these GPSMs, 0.573 ppm deviation of precursor in MS1 was observed on average (Supplementary Fig. 50a) and narrow distribution of precursor mass deviation implies high confidence of Glyco-Decipher identification. Most of precursors are at triple charge state (Supplementary Fig. 50b) which is different from the charge distribution of normal peptides (+2 charge state mostly).

Proteins with one glycosite in it are most in identified glycoproteins and 2.1 glycosites get characterized in each glycoprotein on average (Supplementary Fig. 51a). Low-

density lipoprotein receptor-related protein 2 (UniProt: A2ARV4), which contains 42 glycosites in it, is the protein with most glycosites. At site level, the number of glycan types linked at each site is widely distributed. 30.0% of 2,811 glycan-annotated glycosites have only single glycan composition linked on them and N158 in protein sodium/potassium-transporting ATPase subunit beta-1 (UniProt: P14094) shows most significant glycosylation heterogeneity with 270 different glycans occupied on it (Supplementary Fig. 51b). Glycans in mouse brain have broader size distribution compared with items in four other tissues (Supplementary Fig. 51c). Beside heterogeneity of glycosylation, we also investigated effect of sialic acid on retention time of intact glycopeptides. We classified the glycans in glycopeptides into six categories: 1-3 sialic acids with or without fucose. Retention time (RT) of sialic acid containing glycopeptides were compared with RT of glycans without sialic acid. Supplementary Fig. 51d illustrates the distribution of RT differences between 32,302 sialic acid free GPSMs and corresponding 22,538 sialic acid containing GPSMs with the same peptide backbone. It was found that sialic acid on glycan has retention time delay effect on peptides while influence of fucose was little. This observation is consistent with previous study[10]. The effect of sialic acid on retention time indicates the identified glycans are not random matches, suggests the high confidence of glycan identification in Glyco-Decipher in another aspect.

To get deep insight into glycan occupancy, we depicted co-occurrence of glycan items at site level (Supplementary Fig. 52a). After removing low co-occurrence glycans (less

than 10 times with any other glycans), 281 glycans that tend to appear with others were retained (Supplementary Fig. 52b). Oligo-mannose glycans and their ammonium adducted counterpart tend to appear with other glycans on the same site while many complex/hybrid glycans rarely occupy the same site with others.

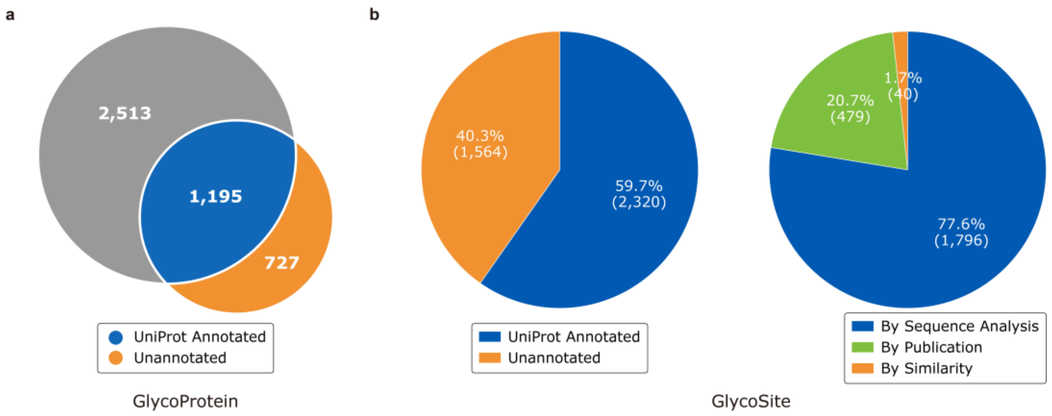

Supplementary Figure 49 Comparison of glycoproteins and glycosites identified by Glyco-Decipher with UniProt annotation.

(a) Glycoproteins identified by Glyco-Decipher and comparison with those reported in UniProt.

(b) Identified glycosites were compared with UniProt database and evidence of glycosites annotated in UniProt were also investigated.

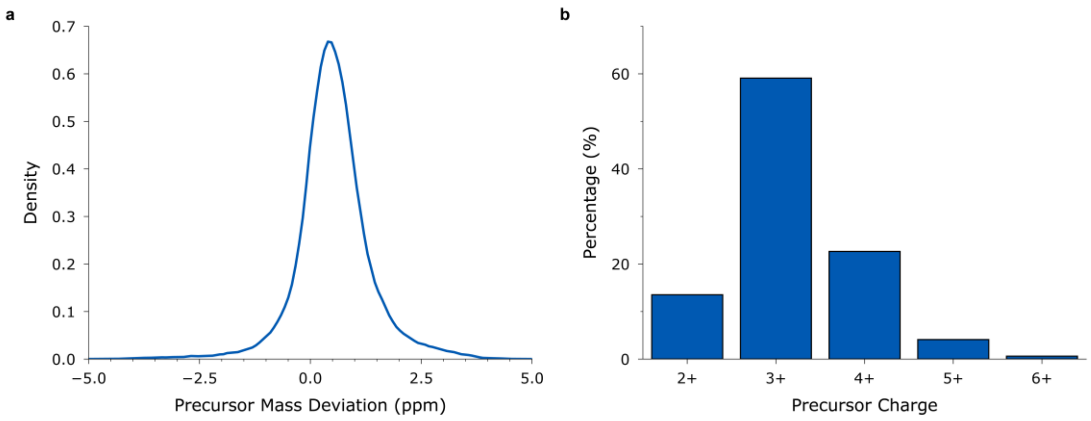

Supplementary Figure 50 Statistics of GPSM identification results from five mouse tissue datasets.

(a) Distribution of precursor mass deviation. Source data are provided as a Source Data file.

(b) Distribution of precursor charge states.

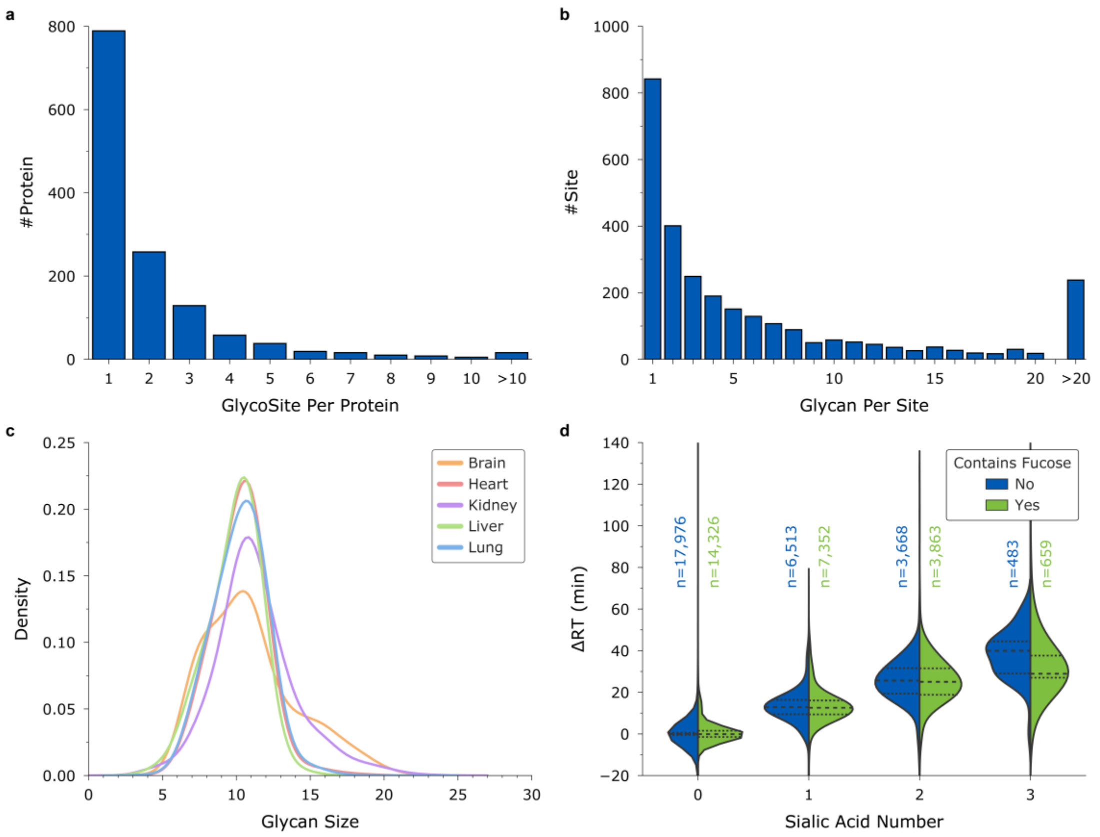

Supplementary Figure 51 Glycosylation heterogeneity revealed by Glyco-Decipher.

(a) Distribution of glycosite number in identified glycoproteins.

(b) Distribution of linked glycan number on each glycosite.

(c) Distribution of glycan size (monosaccharide number) in five mouse tissues. Source data are provided as a Source Data file.

(d) Distribution of retention time differences between 32,302 sialic acid free GPSMs and corresponding 22,538 sialic acid containing GPSMs with same peptide backbone. The quartiles of the distributions are indicated by dashed lines and the spectrum numbers are labeled in the plot. Source data are provided as a Source Data file.

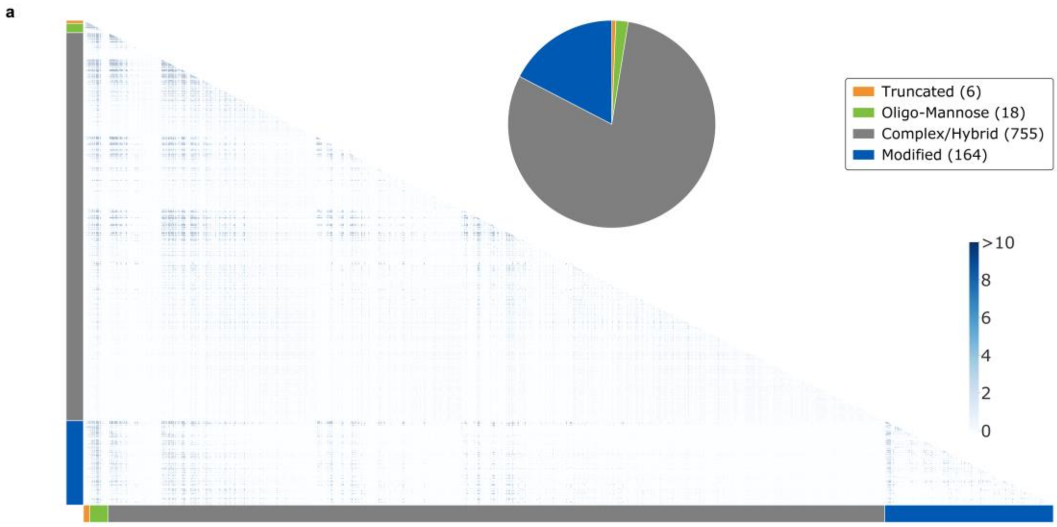

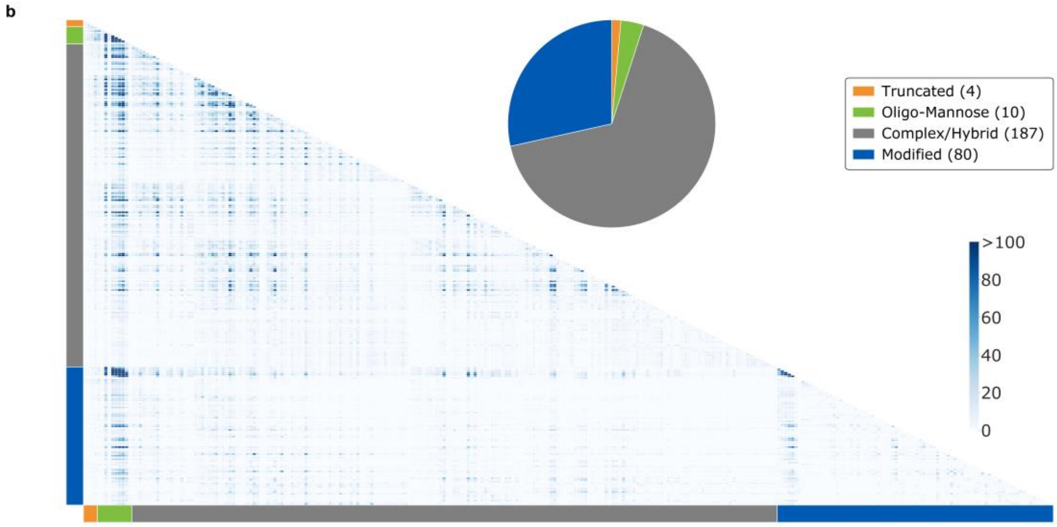

Supplementary Figure 52 Co-occurrence time distribution between glycans. The co-occurrence times between two glycans at site level were investigated.

(a) All 943 glycan items were considered in co-occurrence investigation.

(b) 281 high co-occurrence glycans were investigated after removing items which occur less than 10 times with any other glycans.

Supplementary References

1. Zhu, Z. & Desaire, H. Carbohydrates on Proteins: Site-Specific Glycosylation Analysis by Mass Spectrometry. *Annual Review of Analytical Chemistry* **8**, 463-483 (2015).
2. Liu, M.-Q. et al. pGlyco 2.0 enables precision N-glycoproteomics with comprehensive quality control and one-step mass spectrometry for intact glycopeptide identification. *Nature Communications* **8**, 438 (2017).
3. Fang, P. et al. A streamlined pipeline for multiplexed quantitative site-specific N-glycoproteomics. *Nature Communications* **11**, 5268 (2020).
4. Liu, C. et al. pQuant Improves Quantitation by Keeping out Interfering Signals and Evaluating the Accuracy of Calculated Ratios. *Analytical Chemistry* **86**, 5286-5294 (2014).
5. Zhao, P. et al. Virus-Receptor Interactions of Glycosylated SARS-CoV-2 Spike and Human ACE2 Receptor. *Cell Host & Microbe* **28**, 586-601.e586 (2020).
6. Narimatsu, Y. et al. An Atlas of Human Glycosylation Pathways Enables Display of the Human Glycome by Gene Engineered Cells. *Molecular Cell* **75**, 394-407.e395 (2019).
7. Kuo, C.-W. et al. Distinct shifts in site-specific glycosylation pattern of SARS-CoV-2 spike proteins associated with arising mutations in the D614G and Alpha variants. *Glycobiology*, cwab102 (2021).
8. Zeng, W.-F., Cao, W.-Q., Liu, M.-Q., He, S.-M. & Yang, P.-Y. Precise, fast and comprehensive analysis of intact glycopeptides and modified glycans with pGlyco3. *Nature Methods* **18**, 1515-1523 (2021).
9. Lynn, K.-S. et al. MAGIC: An Automated N-Linked Glycoprotein Identification Tool Using a Y1-Ion Pattern Matching Algorithm and in Silico MS2 Approach. *Analytical Chemistry* **87**, 2466-2473 (2015).
10. Cheng, K. et al. Large-scale characterization of intact N-glycopeptides using an automated glycoproteomic method. *Journal of proteomics* **110**, 145-154 (2014).
