# Supplementary material for: Glyco-Decipher enables glycan database-independent peptide matching and in-depth characterization of site-specific N-glycosylation
Source: Nat Commun. 2022 Apr 7;13:1900. doi: 10.1038/s41467-022-29530-y (PMC8990002; doi:10.1038/s41467-022-29530-y)
Supplement: Supplementary file 3 — Description of Additional Supplementary Files [file 41467_2022_29530_MOESM3_ESM.pdf]

**Description of Additional Supplementary Files**

**File Name:** Supplementary Data 1

**Description:** Glycans in the GlyTouCan database (https://glytoucan.org/).

**File Name:** Supplementary Data 2

**Description:** List of search results for glycopeptide mass spectra from the dataset of mouse tissues (brain, heart, kidney, liver and lung) using Glyco-Decipher.

**File Name:** Supplementary Data 3

**Description:** List of modified glycans and modification moieties discovered from the dataset of mouse tissues (brain, heart, kidney, liver and lung) using Glyco-Decipher.

**File Name:** Supplementary Data 4

**Description:** List of search results for glycopeptide mass spectra from the dataset of $^{13}C/^{15}N$ metabolically labeled yeast using Glyco-Decipher as well as other software tools (StrucGP, pGlyco 3.0, Byonic and MSFragger-Glyco) and analysis results of isotope-based false discovery rate.

**File Name:** Supplementary Data 5

**Description:** List of search results for glycopeptide mass spectra from the dataset of human serum using Glyco-Decipher.

**File Name:** Supplementary Data 6

**Description:** List of search results for glycopeptide mass spectra from the datasets of SARS-CoV-2 Spike and ACE2 using Glyco-Decipher.

**File Name:** Supplementary Data 7

**Description:** Quantification results of the abundance distribution of N-glycosylation on Prosaposin (UniProt: Q61207) in mouse tissues (brain, heart, kidney, liver and lung) using Glyco-Decipher.
