# Supplementary material for: Glyco-Decipher enables glycan database-independent peptide matching and in-depth characterization of site-specific N-glycosylation
Source: Nat Commun. 2022 Apr 7;13:1900. doi: 10.1038/s41467-022-29530-y (PMC8990002; doi:10.1038/s41467-022-29530-y)
Supplement: Supplementary file 11 — Reporting Summary [file 41467_2022_29530_MOESM11_ESM.pdf]

nature research

Corresponding author(s): Mingming Dong; Mingliang Ye

Last updated by author(s): Mar 6, 2022

Reporting Summary

Nature Research wishes to improve the reproducibility of the work that we publish. This form provides structure for consistency and transparency in reporting. For further information on Nature Research policies, see our Editorial Policies and the Editorial Policy Checklist.

Statistics

For all statistical analyses, confirm that the following items are present in the figure legend, table legend, main text, or Methods section.

n/a	Confirmed	
☐	☒	The exact sample size (*n*) for each experimental group/condition, given as a discrete number and unit of measurement
☐	☒	A statement on whether measurements were taken from distinct samples or whether the same sample was measured repeatedly
☒	☐	The statistical test(s) used AND whether they are one- or two-sided<br>*Only common tests should be described solely by name; describe more complex techniques in the Methods section.*
☒	☐	A description of all covariates tested
☒	☐	A description of any assumptions or corrections, such as tests of normality and adjustment for multiple comparisons
☒	☐	A full description of the statistical parameters including central tendency (e.g. means) or other basic estimates (e.g. regression coefficient) AND variation (e.g. standard deviation) or associated estimates of uncertainty (e.g. confidence intervals)
☒	☐	For null hypothesis testing, the test statistic (e.g. *F*, *t*, *r*) with confidence intervals, effect sizes, degrees of freedom and *P* value noted<br>*Give P values as exact values whenever suitable.*
☒	☐	For Bayesian analysis, information on the choice of priors and Markov chain Monte Carlo settings
☒	☐	For hierarchical and complex designs, identification of the appropriate level for tests and full reporting of outcomes
☐	☒	Estimates of effect sizes (e.g. Cohen's *d*, Pearson's *r*), indicating how they were calculated

Our web collection on statistics for biologists contains articles on many of the points above.

Software and code

Policy information about availability of computer code

Data collection	Intact glycopeptides were seperated by an EASY-nLC™ 1200 system (Thermo Scientific, USA), followed by mass spectrometry analysis on an Orbitrap Exploris 480 mass spectrometer (Thermo Scientific, USA). Orbitrap Exploris 480 Tune Application (v3.1.279.9) and Thermo Scientific Xcalibur (v4.4.16.14) were used for the control of mass spectrometer and data collection.
Data analysis	Publicly available N-glycoproteomics datasets were downloaded from the PRIDE data repository, with the accession numbers PXD005411, PXD005413, PXD005412, PXD005553, PXD005555 (mouse tissues, including brain, heart, kidney, liver and lung), PXD005565 (13C/15N metabolically labeled yeast) and PXD019937 (SARS-CoV-2 spike and ACE2).<br>All raw files were converted to the open source format mzML by using ProteoWizard (version 3.0.21105) with 32-bit precision and the “1-” peak picking option.<br>Glyco-Decipher (version 1.0.0, our custom software presented in this paper) was used to search all spectra. Glyco-Decipher can be accessed at https://github.com/DICP-1809/Glyco-Decipher/releases. Glyco-Decipher was implemented in Java (version 11.0.11, https://www.oracle.com/java/).<br>Post-analysis statistics was conducted using Java (version 11.0.11) and Python (version 3.8.3, Anaconda distribution version 4.10.3, https://www.anaconda.com/). The python package “matplotlib” (version 3.4.2), “seaborn” (version 0.11.2) and the JavaScript (version ES2018, https://www.javascript.com/) package “d3” (version 4.13.0) were used for data visualization.

For manuscripts utilizing custom algorithms or software that are central to the research but not yet described in published literature, software must be made available to editors and reviewers. We strongly encourage code deposition in a community repository (e.g. GitHub). See the Nature Research guidelines for submitting code & software for further information.

Data

Policy information about availability of data

All manuscripts must include a data availability statement. This statement should provide the following information, where applicable:

- Accession codes, unique identifiers, or web links for publicly available datasets
- A list of figures that have associated raw data
- A description of any restrictions on data availability

Glycoproteomics raw data were downloaded from the PRIDE Archive with accession number: PXD005411 [https://www.ebi.ac.uk/pride/archive/projects/PXD005411], PXD005413 [https://www.ebi.ac.uk/pride/archive/projects/PXD005413], PXD005412 [https://www.ebi.ac.uk/pride/archive/projects/PXD005412], PXD005553 [https://www.ebi.ac.uk/pride/archive/projects/PXD005553], PXD005555 [https://www.ebi.ac.uk/pride/archive/projects/PXD005555] (Liu et al. mouse tissues, including brain, heart, kidney, liver and lung), PXD005565 [https://www.ebi.ac.uk/pride/archive/projects/PXD005565] (Liu et al. 13C/15N metabolically labeled yeast) and PXD019937 [https://www.ebi.ac.uk/pride/archive/projects/PXD019937] (Zhao et al. SARS-CoV-2 spike and ACE2).
Search results (raw data, output files of Glyco-Decipher, StrucGP, pGlyco 3.0, MSFragger-Glyco and Byonic) that support the findings of this study are available in PRIDE with accession number PXD031032 [https://www.ebi.ac.uk/pride/archive/projects/PXD031032]. The mass spectrometry data of human serum have been deposited to the PRIDE repository with accession number PXD031025 [https://www.ebi.ac.uk/pride/archive/projects/PXD031025]. Swiss-Prot protein databases used in this study have also been deposited to the PRIDE Archive and the GlyTouCan database used in this study is provided in Supplementary Data 1. Source data are provided with this paper.

Field-specific reporting

Please select the one below that is the best fit for your research. If you are not sure, read the appropriate sections before making your selection.

☒ Life sciences ☐ Behavioural & social sciences ☐ Ecological, evolutionary & environmental sciences

For a reference copy of the document with all sections, see nature.com/documents/nr-reporting-summary-flat.pdf

Life sciences study design

All studies must disclose on these points even when the disclosure is negative.

Sample size	The human serum sample was a pooled serum mixture collected from 48 gastric cancer patients and was used to demonstrate the ability of Glyco-Decipher in N-glycopeptide identification. The collected samples were mainly used to generate the mass spectrometry data for the evaluation of software performance. The sample sizes are sufficient to demonstrate the ability of Glyco-Decipher in the glycosylation analysis of complex samples and the identification of glycopeptides with complex glycan compositions.
Data exclusions	No data were excluded in analysis.
Replication	Each intact glycopeptide sample was analyzed twice by LC-MS/MS successfully and the two raw files were combined for data analysis.
Randomization	This is not relevant to this study. The pooled serum samples were used to generate the mass spectrometry data for the evaluation of software performance. No biological conclusions were drawn. No randomization was carried out.
Blinding	Not applicable. The results present in this paper are the output of a software tool and do not require blinding.

Reporting for specific materials, systems and methods

We require information from authors about some types of materials, experimental systems and methods used in many studies. Here, indicate whether each material, system or method listed is relevant to your study. If you are not sure if a list item applies to your research, read the appropriate section before selecting a response.

Materials & experimental systems

n/a	Involved in the study	
☒	☐	Antibodies
☒	☐	Eukaryotic cell lines
☒	☐	Palaeontology and archaeology
☒	☐	Animals and other organisms
☐	☒	Human research participants
☒	☐	Clinical data
☒	☐	Dual use research of concern

Methods

n/a	Involved in the study	
☒	☐	ChIP-seq
☒	☐	Flow cytometry
☒	☐	MRI-based neuroimaging

Human research participants

Policy information about studies involving human research participants

Population characteristics	The human serum samples were collected from forty eight gastric cancer patients. The donors included 5 female participants with an average age of 58.0 years (ages 35-80, standard deviation=14.5) and 43 male participants with an average age of 61.6 years (ages 41-82, standard deviation=9.2).
Recruitment	All human serum samples and clinical information were obtained from Xijing Hospital, Fourth Military Medical University, Xi'an, China. Forty eight gastric cancer patients were recruited randomly from the physical examination and medical therapy population without compensation. There is no potential self-selection bias or other biases that may be present and impact results.
Ethics oversight	All patient samples were collected with informed consent. The study protocol was conducted in accordance to the criteria set by the Declaration of Helsinki and was approved by the Ethical Committee of Xijing Hospital, Fourth Military Medical University, Xi'an, China (Approved NO. of ethic committee: KY20192088-F-1).

Note that full information on the approval of the study protocol must also be provided in the manuscript.
